# Redox-promoted associative assembly of metal–organic materials†
†Electronic supplementary information (ESI) available: Experimental procedures, compound characterization data, and X-ray crystallographic data for compounds **3–7** and **9–11**. CCDC 1405641–1405645, 1429643, 1405647 and 1405648. For ESI and crystallographic data in CIF or other electronic format see DOI: 10.1039/c5sc02214b


**DOI:** 10.1039/c5sc02214b

**Published:** 2015-10-09

**Authors:** Martin Glavinović, Feng Qi, Athanassios D. Katsenis, Tomislav Friščić, Jean-Philip Lumb

**Affiliations:** a Department of Chemistry , McGill University , 801 Sherbrooke St. West Montreal , Quebec H3A 0B8 , Canada . Email: tomislav.friscic@mcgill.ca ; Email: jean-philip.lumb@mcgill.ca ; Fax: +514-398-3797 ; Tel: +514-398-4889

## Abstract

We develop an associative synthesis of metal–organic materials that combines solid-state metal oxidation and coordination-driven self-assembly into a one-step, waste-free transformation. The methodology hinges on the unique reactivity of *ortho*-quinones, which we introduce as versatile oxidants for mechanochemical synthesis. Our strategy opens a previously unexplored route to paramagnetic metal–organic materials from elementary metals.

## Abstract 2

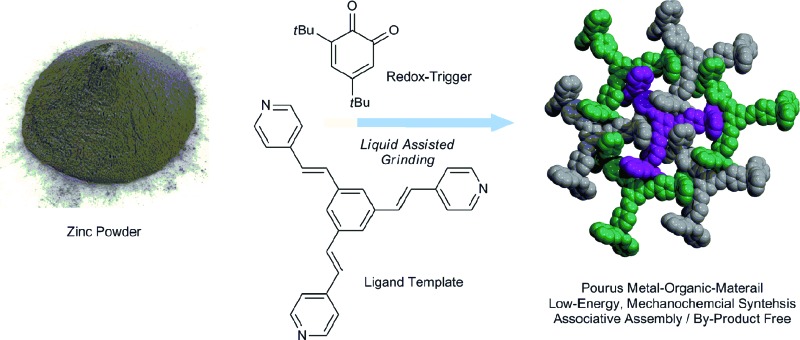
We develop an associative synthesis of metal–organic materials that combines solid-state metal oxidation and coordination-driven self-assembly into a one-step, waste-free transformation.

## Introduction

**Scheme 1:**
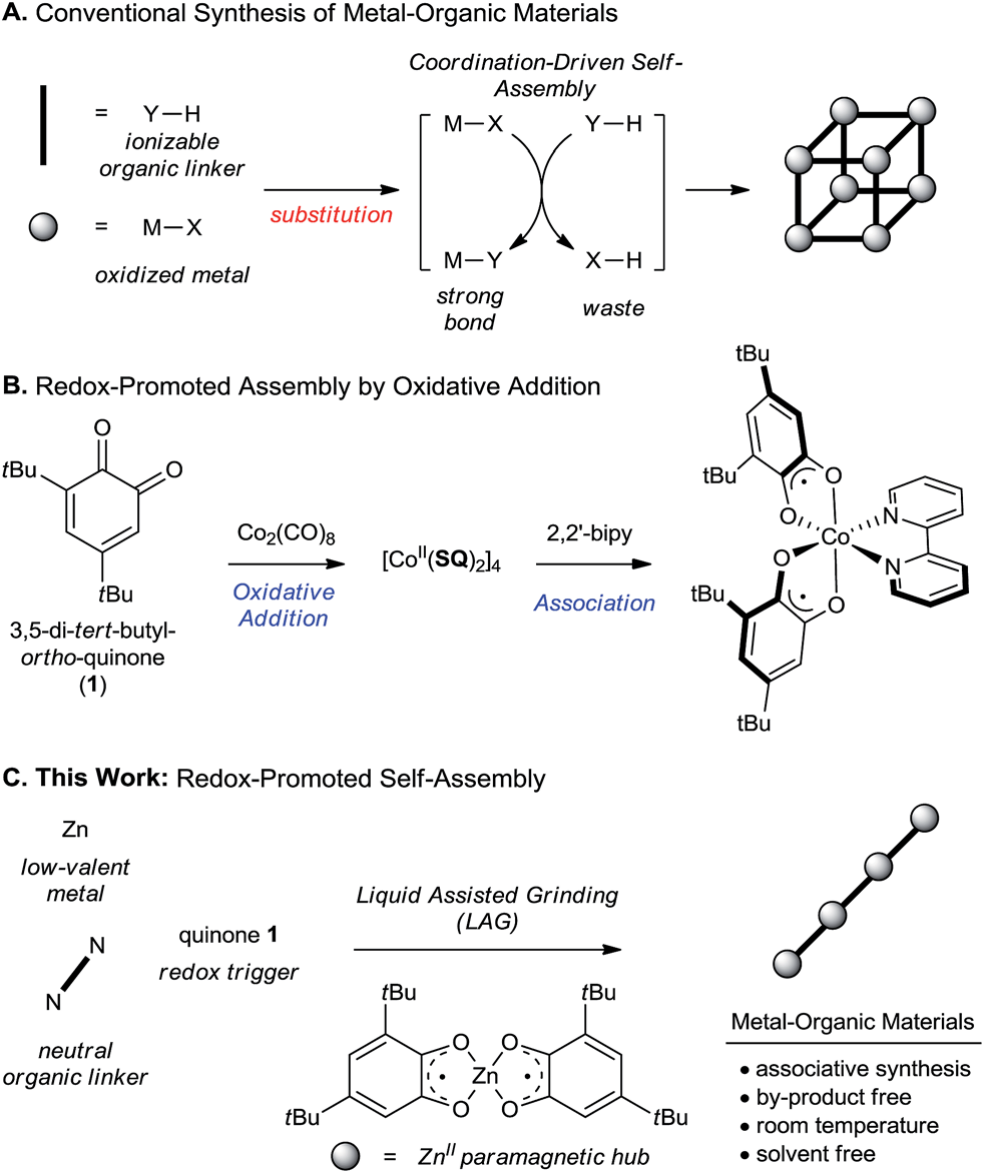
(A) Conventional strategies for the synthesis of metal organic materials by ligand exchange. (B) Oxidative addition of low-valent metals promoted by *ortho*-quinones. (C) Combining metal-oxidation and coordination-driven self-assembly into a single step.

Strategies^1–5^ for the synthesis of metal–organic materials that control pore size, surface area^6^ and the inclusion of functional groups^7^ provide a powerful means of optimizing material properties for energy conversion,^8–10^ gas storage and sensing^11–13^ and catalysis.^14^ While there are now numerous organic linkers, metal-based nodes and a rationale for their assembly,^1–5,15–17^ there has been little development in diversifying the fundamental metal–organic transformations that enable their assembly into solid materials. With few exceptions, coordination bonds used to rigidify and stabilize metal–organic materials are created by ligand exchange at a cationic metal centre.^1–5,15–18^ This is an isohypsic process^19–21^ that requires oxidation of the metal prior to framework assembly. It also creates an unavoidable by-product, which is frequently an acid that must be neutralized (Scheme 1a).^22,23^ These drawbacks negatively impact step- and atom-efficiency, which are increasingly important metrics as metal–organic materials gain traction in commercial applications and industrial manufacturing.^24–27^ A more efficient synthesis would combine metal oxidation and coordination-driven self-assembly into a single, associative transformation. However, conditions used to oxidize bulk metals to their more common binary salts normally require high temperatures and corrosive oxidants (*e.g.* Cl_2_, HCl, HNO_3_)^28,29^ that are incompatible with the mild conditions preferred for the synthesis of metal–organic materials.

In considering this challenge, we were drawn to the chemistry of *ortho*-quinones,^30–34^ which undergo oxidative addition with a range of low-valent organometallic species (Scheme 1b).^35,36^ Ligand ‘non-innocence’ in the resulting semi-quinonate (**SQ**) or catecholate (**Cat**) complexes has been extensively investigated for applications in molecular switching and magnetism.^37,38^ We were attracted to the unique mechanism of metal oxidation, and reasoned that it could be interfaced with coordination-driven self-assembly to provide a one-pot, associative transformation for the synthesis of metal–organic materials based on paramagnetic ‘hub’ metal complexes (Scheme 1c). While attractive, the oxidation of low-valent metals with *ortho*-quinones is relatively unexplored for the synthesis of extended metal–organic materials,^39–41^ and little is known about the reactivity of what would be a paramagnetic hub as defined by our scheme (Scheme 1c).

Our general interest in clean, low-energy synthesis^42–44^ led us to evaluate the reactivity of 3,5-di-*tert*-butyl *ortho*-quinone (**1**) and zinc metal using mechanochemical neat or liquid-assisted grinding (LAG).^45,46^ When compared to conventional solution-based or solvothermal synthesis, mechanochemistry^47–53^ offers a number of advantages, including the absence of a bulk solvent and the ability to activate poorly soluble, inert solids under mild conditions.^54,55^ In this communication, we demonstrate that **1** is a mild and selective metal oxidant that can be interfaced with coordination-driven self-assembly in the solid state. This affords an efficient synthesis of paramagnetic metal–organic materials^56–59^ that proceeds in one step from elementary bulk metals, in a by-product-free process.^60–62^

## Results and discussion

### Synthesis and structures

**Scheme 2:**
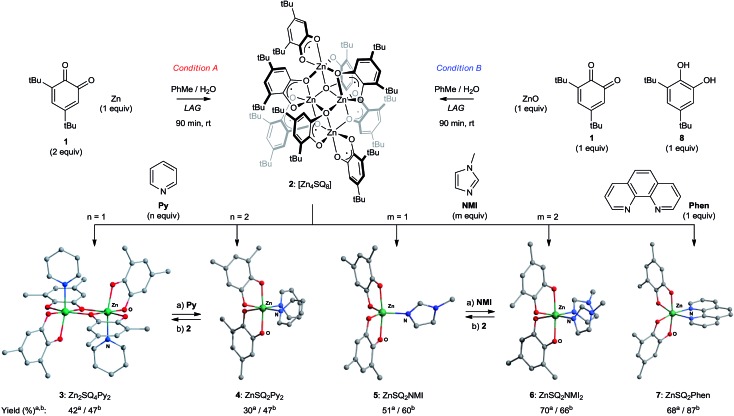
Synthesis and reactivity of a paramagnetic node. ^a^Condition A: Zn (20 mg, 1 equiv.), **1** (2 equiv.), ligand (0–2 equiv.), LAG: 60 μL total volume, 1 : 1 PhMe : H_2_O. ^b^Condition B: ZnO (25 mg, 1 equiv.), **1** (1 equiv.), catechol **8** (1 equiv.), ligand (0–2 equiv.), LAG: 60 μL total volume, 1 : 1 PhMe : H_2_O. Interconversion of **3** and **4**: LAG with (a) **Py** (2 equiv.); (b) **2** (0.25 equiv.). Interconversion of **5** and **6**: LAG with (a) **NMI** (1 equiv.); (b) **2** (0.25 equiv.). By PXRD, crude milled products show quantitative conversion. Reported yields are isolated for recrystallized materials. *tert*-Butyl groups and hydrogen atoms omitted for clarity.

To avoid complications of redox tautomerization and product characterization in the development of this methodology, we chose zinc metal as a point of departure for reaction optimization. Reaction analysis was performed primarily by comparing powder X-ray diffraction (PXRD) patterns of the reaction mixtures to those simulated for crystal structures found in the Cambridge Structural Database (CSD) or determined herein from single crystals obtained by recrystallization of mechanochemical reaction products. Whereas grinding Zn powder (1 equiv.) and **1** (2 equiv.) in the absence of a solvent gave poor conversions and poorly crystalline products, LAG in the presence of a 4 : 1 mixture of toluene (PhMe) and water (H_2_O) (60 μL for 200 mg reaction mixture, corresponding to the *η* value of 0.3 μL mg^–1^)^63^ affords a green crystalline material **2** (condition A, Scheme 1). Analysis by PXRD indicates that **2** is isostructural to the known tetranuclear complex of cobalt(ii): Co_4_ **SQ**_8_ (Scheme 2a).^64^ Formation of **2** is consistent with an overall 2-electron oxidation of Zn^0^ to Zn^II^ by 1-electron reductions of two molecules of **1**. Complex **2** behaves as a kinetically competent paramagnetic hub that is selectively converted into mono- or di-nuclear complexes when ground with a suitable amine. As established by a combination of PXRD and single crystal X-ray diffraction, LAG of **2** in the presence of pyridine (**Py**, 1 equiv. per Zn atom) affords μ-oxo bridged dinuclear complex **3** of composition Zn_2_ **SQ**_4_ **Py**_2_, reflecting the 1 : 1 stoichiometry of Zn to **Py**. When this ratio is 1 : 2, the mononuclear octahedral complex **4** of composition Zn**SQ**_2_ **Py**_2_ is formed selectively, as observed by PXRD. These complexes can be interconverted by adjusting the ratio of Zn to **Py**, so that LAG of **3** in the presence of **Py** (1 equiv.) affords **4** selectively, while LAG of **4** and **2** (0.25 equiv.) affords **3** selectively. Similar behaviour is observed when *N*-methylimidazole (**NMI**) is used in place of **Py**. However, speciation is now between a mononuclear penta-coordinate complex **5** of composition Zn**SQ**_2_ **NMI** and an octahedral complex **6** of composition Zn**SQ**_2_ **NMI**_2_. The expected mononuclear octahedral complex **7** is obtained from LAG of **2** and 1,10-phenanthroline (**Phen**) or, alternatively, by LAG of compounds **3–6** with **Phen** (1 equiv.). As expected from the general stability of complexes with chelating ligands such as **Phen**, ligand exchange was not observed upon grinding **7** with excess **Py**.

Crystal structure analysis of complexes **3–7** reveal carbon–oxygen bond lengths in the range of 1.983–2.137 Å. This is consistent with previously reported structures of metal–semiquinonates,^36^ and supports our assignment of a divalent metal cation coordinated by two semiquinonate radical ligands. Additional support is provided by electron paramagnetic resonance (EPR) measurements for **2–7**, which are consistent with a pair of uncoupled electrons localized on each of the organic ligands (see ESI†).

While the synthesis of **3–7** from **2** demonstrates the feasibility of a paramagnetic hub, there are two important variations of our conditions that can be used to improve efficiency and versatility.(‡
‡Representative procedure: synthesis of **3** [Zn_2_(**SQ**)_4_(**Py**)_2_]: a 10 mL stainless jar was charged with condition A: zinc powder (22.3 mg, 1 equiv.), **1** (150.6 mg, 2 equiv.) and **Py** (47 μL, 1 equiv.). Condition B: zinc oxide powder (23.9 mg, 1 equiv.), **1** (64.6 mg, 1 equiv.), **8** (65.2 mg, 1 equiv.) and **Py** (46 μL, 1 equiv.). With both conditions, 60 μL of a 1 : 1 mixture of PhMe and H_2_O was added by micropipette along with two stainless steel balls of 7 mm diameter (weight 1.3 grams). The mixture was then milled for 90 min with a Retsch MM200 mill at a frequency of 25 Hz. The crude product was collected and analysed by PXRD, FT-IR, EPR and TGA. Details of all mechanochemical experiments; single crystal recrystallization conditions; crystal structure parameters; experimental PXRD patterns, TGA thermograms, FTIR-ATR and EPR spectra of the metal–organic complexes; as well as organic ligand synthesis is described in the ESI.†
) The first involves the direct synthesis of complexes **3–7** from Zn powder, **1** and the appropriate nitrogen ligand in a 1-pot process that is completely selective for the intended product. This improves synthetic efficiency by obviating the need to isolate **2** before ligand association. Alternatively, the synthesis of **2–7** can be performed from zinc oxide (ZnO), which is an isohypsic process for the metal (condition B, Scheme 2). To compensate for the +2 oxidation state of Zn, a 1 : 1 mixture of quinone **1** and its corresponding catechol **8** are ground with ZnO. To our knowledge, this is first time that a metal–**SQ** complex has been assembled by conproportionation of a catechol and a quinone, highlighting the compatibility of our conditions with either ligand–metal or ligand–ligand redox processes.^65,66^

**Scheme 3:**
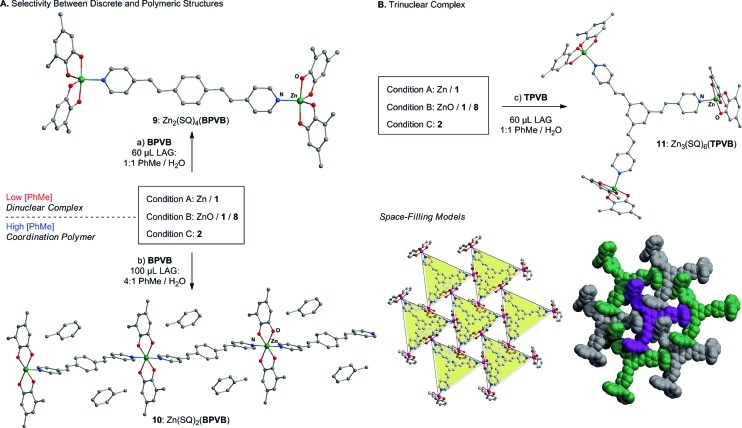
Synthesis and reactivity of a dinuclear complex/coordination polymer and a trinuclear complex. Condition A: Zn (20 mg, 1 equiv.), **1** (2 equiv.), condition B: ZnO (25 mg, 1 equiv.), **1** (1 equiv.), **8** (1 equiv.); condition C: **2** (150 mg, 0.25 equiv.); (a) **BPVB** (0.5 equiv.), LAG: 60 μL, 1 : 1 PhMe : H_2_O; (b) **BPVB** (1 equiv.), LAG: 100 μL, 4 : 1 PhMe : H_2_O; (c) **TPVB** (1 equiv.), LAG: 60 μL, 1 : 1 PhMe : H_2_O. *tert*-Butyl groups and hydrogen atoms are omitted for clarity.

Selectivity between **3** and **4**, or between **5** and **6** is not observed under conventional solvothermal conditions, which afforded complex mixtures, as determined by PXRD, across a range of ligand to Zn ratios (see ESI†). This highlights the remarkable ability of mechanochemistry to direct the outcome of a reaction by precise reagent stoichiometry.^67–69^ A more subtle structure-directing effect of the liquid additive is observed during the synthesis of extended metal–organic materials based on the ditopic ligand 1,4-bis(2-(pyridin-4-yl)vinyl)benzene (**BPVB**) (Scheme 3). LAG in the presence of a 1 : 1 mixture of PhMe and H_2_O affords the discrete binuclear complex [Zn(**SQ**)_2_]_2_(**BPVB**) **9**, whereas a 4 : 1 ratio of PhMe to H_2_O returns the linear coordination polymer **10** in the form of a PhMe solvate.^70,71^ This represents the first example where a coordination polymer is assembled directly from an elementary metal by an entirely associative, waste-free process.^39–41^ The ability to select between dinuclear complex **9** and polymeric **10** by switching the composition of the milling liquid demonstrates the potential for LAG to reveal and screen for structure- and composition-directing effects of simple organic solvent molecules. Such subtle effects have been observed and systematically utilized during the mechanochemical assembly of hydrogen-bonded frameworks,^72,73^ but are otherwise unexplored for controlling selectivity during coordination-driven self-assembly in the solid state.

The synthesis of a more complex solid, capable of guest inclusion, results from LAG of Zn metal and **1** with the tripodal ligand 1,3,5-tris(2-(pyridin-4-yl)vinyl)benzene (**TPVB**). Milling in the presence of a 1 : 1 mixture of PhMe and H_2_O affords the trinuclear complex **11**, composed of three pentacoordinate Zn(**SQ**)_2_ units surrounding a central **TPVB** ligand.(§
§Crystallographic data: compound **3**: Zn_2_ **SQ**_4_ **Py**_2_, (CCDC 1405641), orthorhombic, *Pbca*, *a* = 10.8810(16) Å, *b* = 19.5543(28) Å, *c* = 29.4419(42) Å, *Z* = 4, *R*_1_ = 0.083, w*R*_2_ = 0.136 (for 4206 reflections with *I* ≥ 2*σ*_I_), *R*_1_ = 0.177, w*R*_2_ = 0.163 (for all reflections), *S* = 1.056; compound **4**: Zn**SQ**_2_ **Py**_2_, (CCDC 1405642), monoclinic, *P*2_1_/*c*, *a* = 10.671(5) Å; *b* = 19.665(9) Å; *c* = 17.723(8) Å; *β* = 97.137(7)°; *Z* = 4, *R*_1_ = 0.108, w*R*_2_ = 0.137 (for 3107 reflections with *I* ≥ 2*σ*_I_), *R*_1_ = 0.108, w*R*_2_ = 0.193 (for all reflections), *S* = 1.015; compound **5**: Zn**SQ**_2_ **NMI**, (CCDC 1405643), orthorhombic, *Pbca*, *a* = 12.2437(8) Å, *b* = 18.3841(11) Å, *c* = 30.3846(19) Å, *Z* = 8, *R*_1_ = 0.049, w*R*_2_ = 0.093 (for 3668 reflections with *I* ≥ 2*σ*_I_), *R*_1_ = 0.120, w*R*_2_ = 0.126 (for all reflections), *S* = 0.992; compound **6**: Zn**SQ**_2_ **NMI**_2_, (CCDC 1405644), monoclinic, *P*2_1_, *a* = 10.6541(9) Å, *b* = 19.7432(17) Å, *c* = 17.8899(15) Å, *β* = 99.193(1)°, *Z* = 4, *R*_1_ = 0.058, w*R*_2_ = 0.137 (for 4265 reflections with *I* ≥ 2*σ*_I_), *R*_1_ = 0.108, w*R*_2_ = 0.163 (for all reflections), *S* = 1.016; compound **7**: Zn**SQ**_2_ **Phen**, (CCDC 1405645) monoclinic, *P*2_1_/*c*, *a* = 10.404(3) Å, *b* = 32.256(8) Å, *c* = 13.672(3) Å, *β* = 111.664(4)°, *Z* = 4, *R*_1_ = 0.081, w*R*_2_ = 0.175 (for 2973 reflections with *I* ≥ 2*σ*_I_), *R*_1_ = 0.289, w*R*_2_ = 0.297 (for all reflections), *S* = 1.066; compound **9**: [Zn(**SQ**)_2_]_2_(**BVPB**), (CCDC 1429643), monoclinic, *P*21/*n*, *a* = 12.6164(1) Å, *b* = 18.4537(2) Å, *c* = 15.3934(2) Å, *β* = 103.538(1)°, *Z* = 4, *R*_1_ = 0.0334, w*R*_2_ = 0.1042 (for 5329 reflections with *I* ≥ 2*σ*_I_), *R*_1_ = 0.0428, w*R*_2_ = 0.1152 (for all reflections), *S* = 0.854; compound **10**: [Zn(**SQ**)_2_](**BVPB**)·toluene, (CCDC 1405647), monoclinic, *P*2_1_/*n*, *a* = 7.6947(9) Å, *b* = 13.1945(15) Å, *c* = 27.271(3) Å, *β* = 95.507(2)°, *Z* = 2, *R*_1_ = 0.058, w*R*_2_ = 0.117 (for 3753 reflections with *I* ≥ 2*σ*_I_), *R*_1_ = 0.132, w*R*_2_ = 0.143 (for all reflections), *S* = 1.001; compound **11**: [Zn(**SQ**)_2_]_3_(**TVPB**)·toluene solvate, (CCDC 1405648) triclinic, *P*1, *a* = 15.4724(16) Å, *b* = 15.4781(16) Å, *c* = 28.908(3) Å, *α* = 78.730(4)°, *β* = 88.358(4)°, *γ* = 60.122(3)°, *Z* = 2, *R*_1_ = 0.1496, w*R*_2_ = 0.3209 (for 15 246 reflections with *I* ≥ 2*σ*_I_), *R*_1_ = 0.1811, w*R*_2_ = 0.3344 (for all reflections), *S* = 1.216.) Crystal structure analysis reveals that the trigonal complexes arrange into a loosely packed structure^74–76^ with highly disordered solvent molecules occupying otherwise vacant cavities. According to thermogravimetric analysis, the included solvent is removed by heating at 50 °C under reduced pressure. Importantly, the PXRD patterns taken before and after desolvation reveal minimal changes to the crystal structure of the material. Consequently, **11** represents a unique example where a guest molecule is removed from a paramagnetic lattice host structure without disruption of crystallinity.^77,78^

**Fig. 1:**
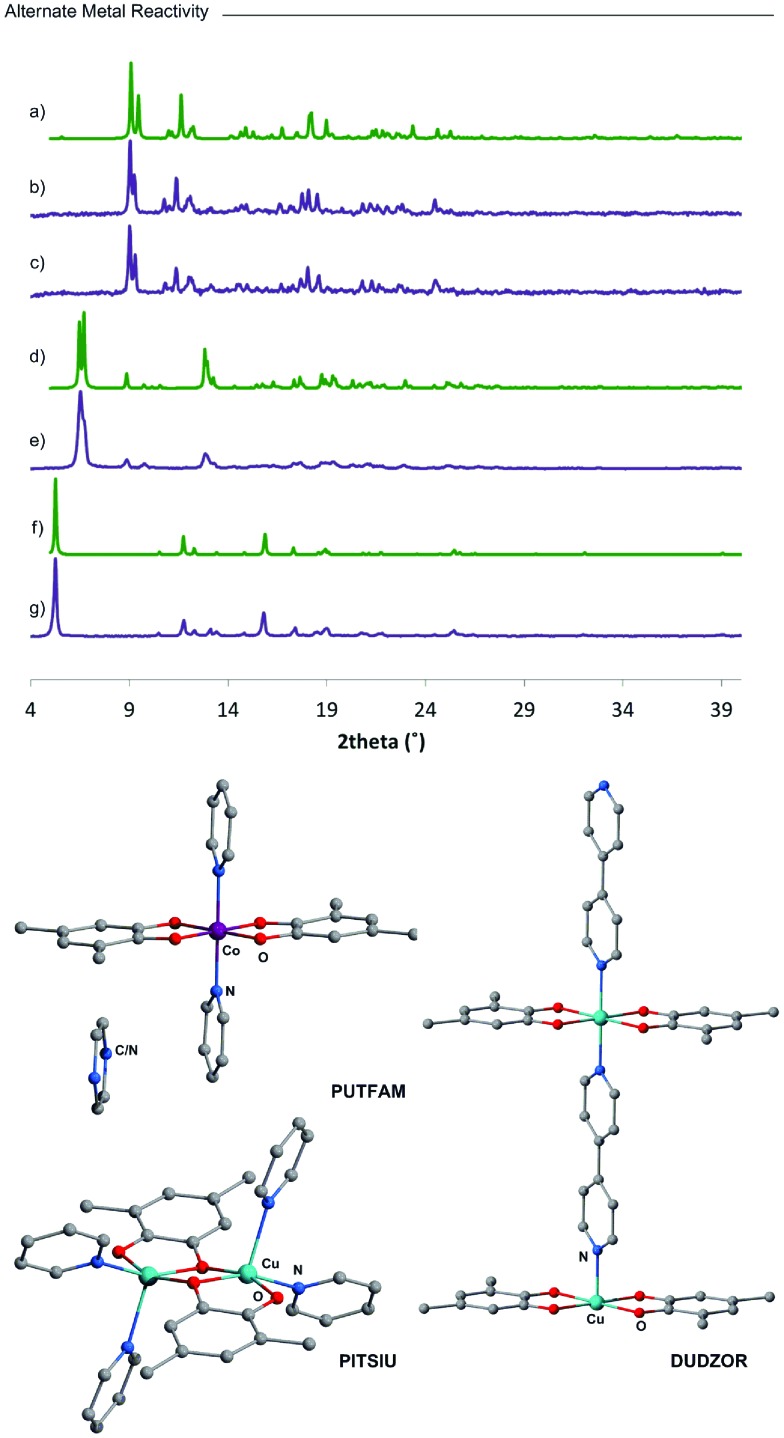
LAG reactivity with Co, Mn and Cu. Conditions: 200 mg total, LAG: PhMe : H_2_O (1 : 1, 60 μL). (a) Simulated PXRD pattern of Co(iii)(**SQ**)(**Cat**)(**Py**)_2_-(**Py**)_0.5_ CCDC code PUTFAM; (b) crude milled product of Co (1 equiv.), 1 (2 equiv.) and **Py** (2.5 equiv.) LAG 1 : 1 PhMe/H_2_O (product 12); (c) crude milled product of Mn (1 equiv.), 1 (2 equiv.) and **Py** (3 equiv.) LAG 1 : 1 PhMe/H_2_O (Product 13); (d) simulated PXRD pattern of Cu^II^_2_(**Cat**)_2_(**Py**)_4_-(**Py**)_0.5_ CCDC code PITSIU; (e) crude milled product of Cu (2 equiv.), 1 (2 equiv.), **Py** (4.5 equiv.), neat (product 14); (f) simulated PXRD pattern of Cu^II^(**SQ**)_2_(4,4′-bipyridine) CCDC code DUDZOR; (g) crude milled product of Cu (1 equiv.), 1 (2 equiv.), 4,4′-bipyridine (1 equiv.) LAG 1 : 1 PhMe/H_2_O (product 15). *tert*-Butyl groups and hydrogen atoms are omitted for clarity.

Our solid-state, redox-promoted self-assembly is not restricted to zinc metal, and our mechanochemical reaction conditions are readily extended to the synthesis of discrete or extended structures of cobalt, copper and manganese metals (Fig. 1). The semi-quinonate and catecholate complexes of these metals have been extensively investigated for valence tautomerism.^37^ We find that milling the Co, Mn or Cu metal powder with **1** and **Py** affords mononuclear octahedral Co^II/III^ complex **12**, an isostructural and previously not reported Mn complex **13**, and a μ-oxo bridged dinuclear Cu^II^ complex **14**. The structures of **12**, **13** and **14** were confirmed by comparing the PXRD patterns of the milled products to simulated patterns generated from the known crystal structures (CCDC codes: PUTFAM for isostructural **12** and **13**, and PITSIU for **14**).^79,80^ In addition to their complementary nuclearity, these complexes result from distinct 2- or 3-electron oxidations of Cu, Co and Mn, respectively. A similar 2-electron oxidation of metallic Cu is also possible by LAG with the ditopic ligand 4,4′-dipyridyl (**BIPY**), which provides the known linear coordination polymer **15**, possessing a Cu^II^–**SQ**_2_ repeat unit (CCDC code DUDZOR).^40^ This demonstrates the viability of our methodology to the low-energy synthesis of redox-labile metal complexes that have been extensively investigated for applications in molecular switching and magnetism.^35–38^

## Conclusions

In summary, we have demonstrated that metal-oxidation and coordination-driven self-assembly can be interfaced into a one-pot and multi-component strategy to generate discrete or extended metal–organic architectures. This work highlights the unique oxidation of elementary metals mediated by *ortho*-quinone **1**, which is a new reagent for mechanochemical synthesis of metal–organic materials. It also explores the fundamental reactivity of semi-quinonate–metal complexes, and demonstrates their viability as kinetically competent, paramagnetic hubs. This sets the stage for an entirely associative and waste-free synthesis of well-defined metal–organic frameworks that should interface with our existing knowledge of coordination-driven self-assembly. Our work demonstrates how unconventional strategies for synthesis can dramatically improve its overall efficiency. In this case, efficiency is achieved by combining the fundamental steps of metal oxidation and ligand association into a single transformation.
