# Supplementary material for: Redox-promoted associative assembly of metal–organic materials
Source: Chem Sci. 2015 Oct 9;7(1):707–12. doi: 10.1039/c5sc02214b (PMC5530005; doi:10.1039/c5sc02214b)
Supplement: Supplementary file 1 [file SC-007-C5SC02214B-s001.pdf]

Electronic Supplementary Material (ESI) for Chemical Science.
This journal is © The Royal Society of Chemistry 2015

Electronic Supplementary Information

Controlled assembly of complex metal-organic architectures from metals by redox-promoted mechanochemical self-assembly

Martin Glavinovic, Feng Qi, Athanassios D. Katsenis,
Tomislav Friščić and Jean-Philip Lumb**

Department of Chemistry and FRQNT Centre for Green Chemistry and Catalysis
McGill University, 801 Sherbrooke St. W. Room 300, Montreal, QC, H3A 0B8, Canada
tomislavfriscic@gmail.com; jean-philip.lumb@mcgill.ca

Table of Contents

1. **Experimental Details**
   - **1.1. General Experimental**
   - **1.2. Instrumentation**
     - *1.2.1. Single Crystal X-Ray Diffraction (XRD)*
     - *1.2.2. Powder X-Ray Diffraction (PXRD)*
     - *1.2.3. Fourier-Transform infrared attenuated total reflectance (FTIR-ATR)*
     - *1.2.4. Thermogravimetric Analysis (TGA)*
     - *1.2.5. Nuclear Magnetic Resonance Spectroscopy (NMR)*
     - *1.2.6. Electron Paramagnetic Resonance Spectroscopy (EPR)*
   - **1.3. Mechanochemical Experiments with respective PXRD patterns**
     - *1.3.1. Synthesis of* $[Zn(3,5\text{-}dtbsq)_2]_4$ *tetramer* (**2**)
       - *1.3.1.1. From Zinc powder*
       - *1.3.1.2. From Zinc Oxide*
     - *1.3.2. Synthesis of* $Zn_2(3,5\text{-}dtbsq)_4(pyridine)_2$ (**3**) *and* $Zn(3,5\text{-}dtbsq)_2(pyridine)_2$ (**4**)
       - *1.3.2.1. From Zinc powder*
       - *1.3.2.2. From Zinc Oxide*
       - *1.3.2.3. From complex* **2**
     - *1.3.3. Synthesis of* $Zn(3,5\text{-}dtbsq)_2(N\text{-}Methyl\ Imidazole)_1$ (**5**) *and* $Zn(3,5\text{-}dtbsq)_2(N\text{-}Methyl\ Imidazole)_2$ (**6**)
       - *1.3.3.1. From Zinc powder*
       - *1.3.3.2. From Zinc Oxide*
       - *1.3.3.3. From complex* **2**
     - *1.3.4. Synthesis of* $Zn(3,5\text{-}dtbsq)_2(1,10\text{-}phenanthroline)$ (**7**)
       - *1.3.4.1. From Zinc powder*
       - *1.3.4.2. From Zinc Oxide*
       - *1.3.4.3. From complex* **2**
     - *1.3.5. Synthesis of* $Zn_2(3,5\text{-}dtbsq)_4(1,4\text{-}bis(2\text{-}(pyridin\text{-}4\text{-}yl)vinyl)benzene)$ (**9**) and $Zn(3,5\text{-}dtbsq)_2(1,4\text{-}bis(2\text{-}(pyridin\text{-}4\text{-}yl)vinyl)benzene)$ (**10**)
       - *1.3.5.1. From Zinc powder*
       - *1.3.5.2. From Zinc Oxide*
       - *1.3.5.3. From complex* **2**
     - *1.3.6. Synthesis of* $Zn_3(3,5\text{-}dtbsq)_6(1,3,5\text{-}tris(2\text{-}(pyridin\text{-}4\text{-}yl)vinyl)benzene)$ (**11**)
       - *1.3.6.1. From Zinc powder*
       - *1.3.6.2. From Zinc Oxide*
       - *1.3.6.3. From complex* ***2***
     - *1.3.7. Ligand Interconversion*
       - *1.3.7.1. Interconversion of pyridine complexes* **3** *and* **4**
       - *1.3.7.2. Interconversion of N-Methyl Imidazole complexes* **5** *and* **6**
       - *1.3.7.3. Interconversion of pyridine and N-Methyl Imidazole complexes* ***3, 4, 5, 6*** *to 1,10-phenanthroline complex* ***7***;
     - *1.3.8. Alternate Metal Reactivity*
   - **1.4. Solvothermal Synthesis with respective PXRD patterns**
     - *1.4.1. Solvothermal Synthesis of complex* **2** *from Zinc Powder*

1.4.2. Solvothermal Synthesis of complex **2** *from Zinc Oxide*
1.4.3. Solvothermal Synthesis of complexes **3** *and* **4**
1.4.4. Solvothermal Synthesis of complexes **5** *and* **6**
1.4.5. Solvothermal Synthesis of complex **7**
1.4.6. Solvothermal Synthesis of complexes **9** *and* **10**
1.4.7. Solvothermal Synthesis of complex **11**

1.5. Conditions for single crystal growth by recrystallization

*1.5.1. Zn_2(3,5-dtbsq)$_4$(pyridine)$_2$ (**3**)*
*1.5.2. Zn(3,5-dtbsq)$_2$(pyridine)$_2$ (**4**)*
*1.5.3. Zn(3,5-dtbsq)$_2$(N-Methyl Imidazole) (**5**)*
*1.5.4. Zn(3,5-dtbsq)$_2$(N-Methyl Imidazole)$_2$ (**6**)*
*1.5.5. Zn(3,5-dtbsq)$_2$(1,10-phenanthroline) (**7**)*
*1.5.6. Zn_2(3,5-dtbsq)$_4$(1,4-bis(2-(pyridin-4-yl)vinyl)benzene) (**9**)*
*1.5.7. Zn(3,5-dtbsq)$_2$(1,4-bis(2-(pyridin-4-yl)vinyl)benzene) (**10**)*
*1.5.8. Zn3(3,5-dtbsq)6(1,3,5-tris(2-(pyridin-4-yl)vinyl)benzene) (**11**)*

1.6. Solvent Evacuation of Zn_3(3,5-dtbsq)$_6$(TPVB)

1.6.1. PXRD patterns
1.6.2. TGA thermograms

1.7. Ligand Synthesis

1.7.1. 1,4-bis(2-(pyridin-4-yl)vinyl)benzene (**BPVB**)
1.7.2. 1,3,5-tris(2-(pyridin-4-yl)vinyl)benzene (**TPVB**)

2. **Rendered Crystal Structures of complexes 3-7; 9-11**
3. **FTIR-ATR Spectra**
4. **UV-Vis Spectra**
5. **EPR Spectra**
6. **TGA Thermograms**

1. Experimental Details

1.1 General Details

Reagents were purchased from Sigma Aldrich (SA) and used without purification. Zinc powder (Zn) purum 99% (SA Code: 14409) was used as received, and was not activated prior to milling. Zinc oxide (ZnO) powder puriss >99.0% (SA Code: 96479); Cobalt (Co) powder puriss >99.8% (SA Code: 60784); Manganese (Mn) powder 325 mesh (Alfa Aesar Code: 00531); Copper powder (Cu) 200 mesh (SA Code: 31284) were used as received. All solvents were dried and purified using an Innovative Technology PureSolv MD 7 or MBraun MB SPS 800, and water (H2O used for liquid assisted grinding (LAG) was distilled but not deionized.

1.2 Instrumentation

1.2.1 Single Crystal X-ray diffraction (XRD)

Single crystal X-ray diffraction data was collected on a Bruker APEX II diffractometer with MoKα (λ=0.71073 Å) source and CCD detector at 100 K, unless otherwise specified. The structures were determined by least squares refinement against F^2 using SHELX-2014 software.

1.2.2 Powder X-ray Diffraction (PXRD)

Powder X-ray diffraction patterns were collected using a Bruker D2 powder diffractometer equipped with a Cu-Kα (λ=1.54060 Å) source and Lynxeye detector set at a discriminant range of 0.110 V to 0.250 V. The patterns were collected in the range of 4° to 40°. Analysis of PXRD patterns was conducted using Panalytical X'Pert Highscore Plus software. Experimental patterns were compared to simulated patterns calculated from published crystal structures using Mercury crystal structure viewing software. Crystallographic Information Files containing published crystal structures were obtained from the Cambridge Structural Database (CSD) and Crystallography Open Database (COD).

1.2.3 Fourier-transform infrared attenuated total reflection (FTIR-ATR)

FTIR-ATR spectra were collected using a Bruker Vertex 70 FTIR-ATR spectrometer in the range 400 cm-1 to 4000 cm-1. FTIR spectra were analyzed using Bruker OPUS software.

1.2.4 Ultraviolet-Visible Absorption Spectra

UV-Vis spectra were collected on a Jasco V-670 Spectrophotometer in the range 400-1100 cm^{-1}. Samples were dissolved in anhydrous dichloromethane (10^{-3}M) and measured in quartz cuvettes. Due to their low solubility, complexes **9-11** were not measured as heterogeneous products were obtained.

1.2.5 Thermogravimetric analysis (TGA)

Thermograms of crude products were collected using a TA Instruments TGA Q500 thermogravimetric analyser, at a heating rate of 10°C/min from 45°C to 600°C under an air

atmosphere. The flow rates of the purge gas (N_2) and sample gas (air) were set at 50 mL/min and 50 mL/min respectively. TGA curves were analyzed with TA Universal Analysis software.

1.2.6 Nuclear Magnetic Resonance Spectroscopy (NMR)

^{1}H-NMR spectra were obtained on either a Bruker 500 MHz AV500 spectrometer or an Agilent (Varian) 400 MHz spectrometer.

1.2.7 Electron Paramagnetic Resonance Spectroscopy (EPR)

Crude milled products were dissolved in anhydrous toluene (0.001 M), and 0.1 mL of solution were syringed into 4 mm EPR tubes purchased from Wilmad-Lab glass and degassed by freeze pump thaw in liquid nitrogen. The resulting spectra were collected on a Bruker Electron Paramagnetic resonance spectrometer at 77 K. X-Band 9.8 GHz with a spectral range of 400-6400 Gauss.

1.3 Mechanochemical Experiments with respective PXRD patterns

Standard reaction conditions: 200 mg of reactants (total mass of all components) were added in appropriate stoichiometric ratios to a 10 mL stainless steel grinding jar along with two 0.6 cm diameter stainless steel balls. The mixture was milled for 90 mins with a Retsch MM200 mill at a frequency of 25 Hz. Milling by LAG: For a 200 mg scale reaction, 60 μL of a 1:1 by volume mixture of PhMe and H_2O was added to the solid reactants by micropipette.

Following completion of the reaction, crude products were collected from the stainless steel jar and analyzed by PXRD, FTIR, EPR and TGA. Single crystal recrystallization conditions were specific to the complex and can be found in section 1.5.

1.3.1 *Synthesis of [Zn(3,5-dtbsq)$_2$]$_4$ tetramer* **(2)**

1.3.1.1 *From Zinc powder*

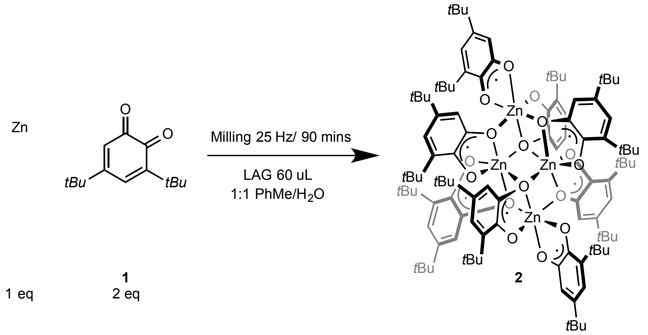

Scheme S1: Zn (25.8 mg, 0.395 mmol, 1 equiv), **1** (174.2 mg, 0.791 mmol, 2 equiv).

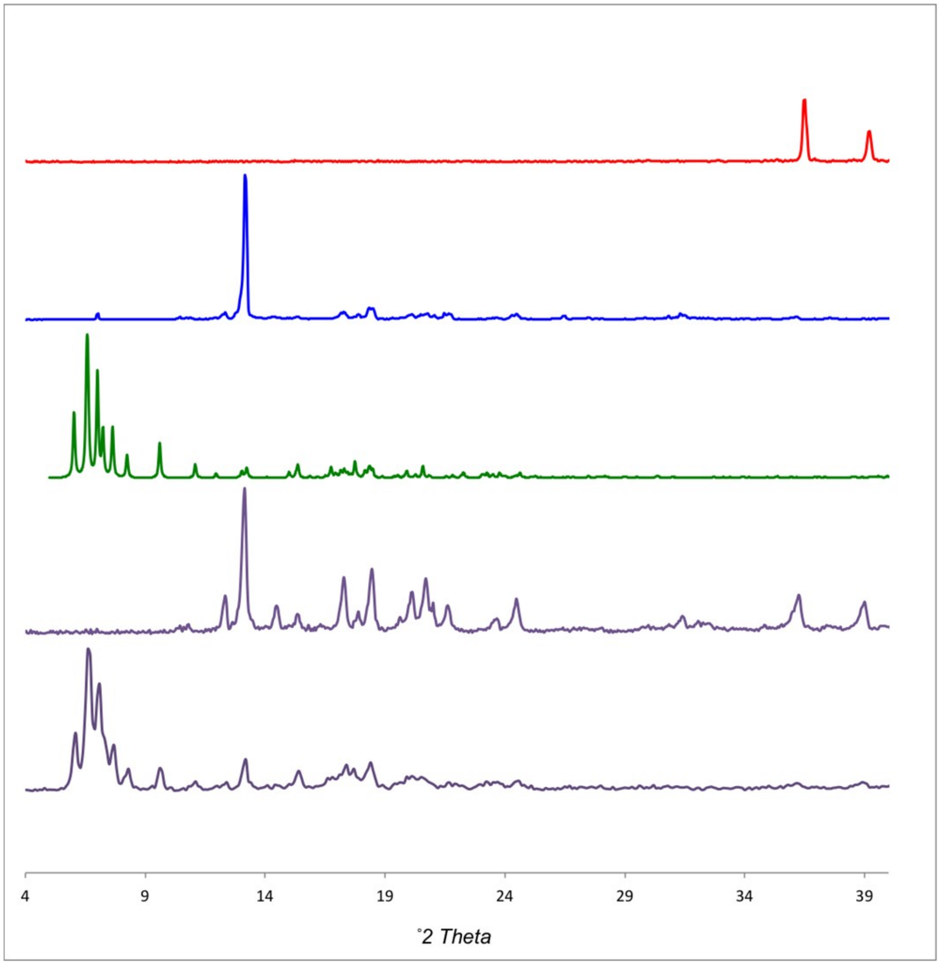

Figure S1. Powder X-ray diffraction data for the synthesis of **2** from Zn: a) Zn; b) **1**; c) Simulated pattern of [Co^{II}(3,5-dtbsq)$_2$]$_4$(benzene) tetranuclear complex TBSQCO from CCDC database; d) Crude milled product of Zn (1 equiv), **1** (2 equiv) NEAT; e) Crude milled product of Zn (1 equiv), **1** (2 equiv) LAG 1 to 1 PhMe/H_2O

1.3.1.2 *From Zinc Oxide*

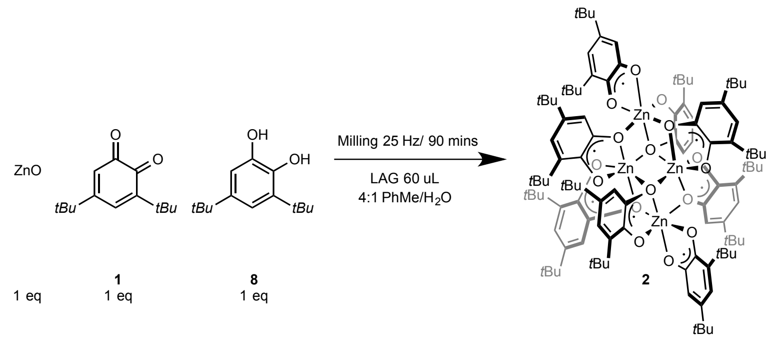

Scheme S2: ZnO (31.1 mg, 0.382 mmol, 1 equiv); **1** (84.1 mg, 0.382 mmol, 1 equiv); **8** (84.9 mg, 0.382 mmol, 1 equiv).

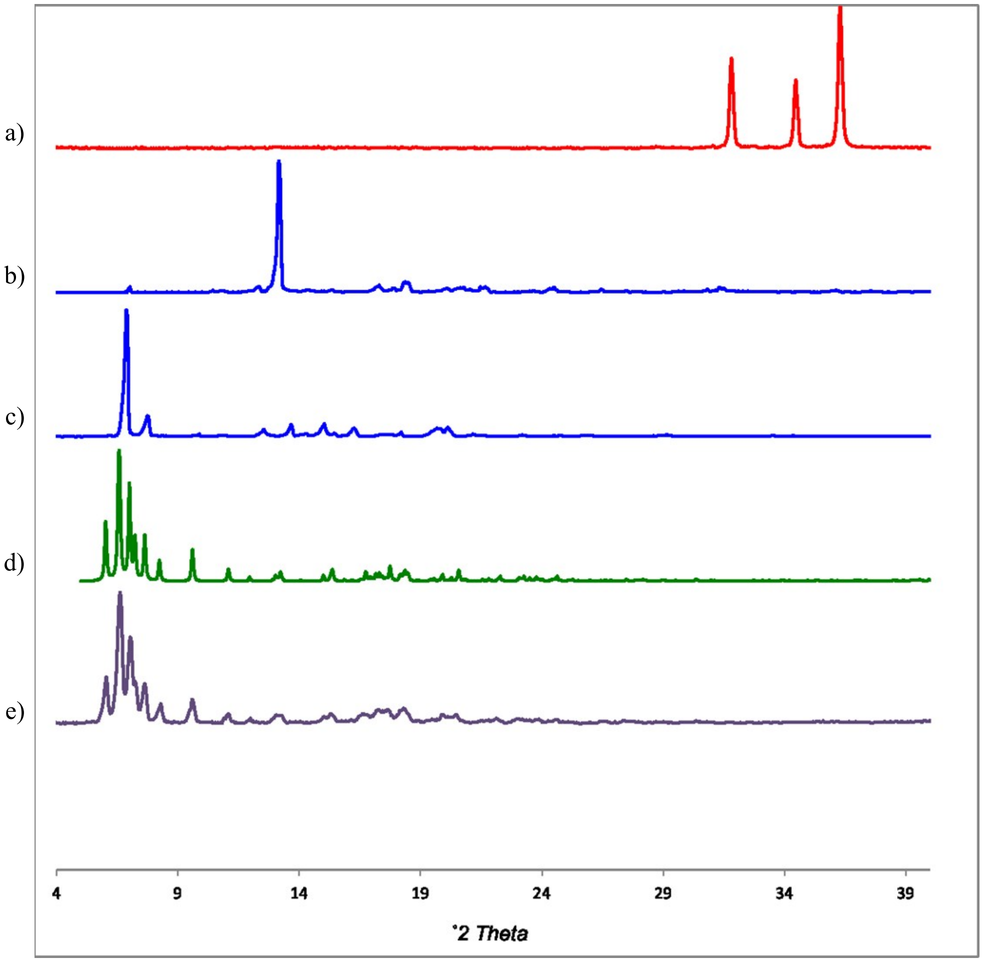

Figure S2. Powder X-ray diffraction data for the synthesis of **2** from ZnO: a) ZnO; b) **1**; c) **8**; d) Simulated pattern of $[Co^{II}(3,5\text{-dtbsq})_2]_4$(benzene) tetranuclear complex TBSQCO from CCDC database; e) Crude milled product of ZnO (1 equiv), **1** (1 equiv), **8** (1 equiv)

*1.3.2 Synthesis of Zn₂(3,5-dtbsq)₄(pyridine)₂ (**3**) and Zn(3,5-dtbsq)₂(pyridine)₂ (**4**)*

1.3.2.1 From Zinc powder

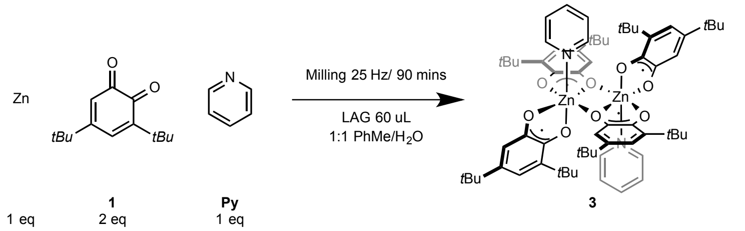

Scheme S3: Zn (0.342 mmol, 22.3 mg, 1 equiv); **1** (0.684 mmol, 150.6 mg, 2 equiv); pyridine (**Py**) (0.342 mmol, 27.0 mg, 27.5 uL, 1 equiv)

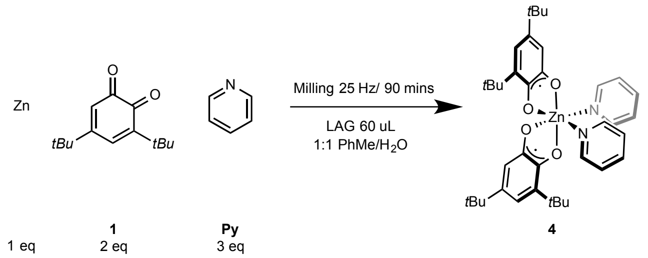

Scheme S4: Zn (0.269 mmol, 17.6 mg, 1 equiv); **1** (0.538 mmol, 118.6 mg, 2 equiv); **Py** (0.807 mmol, 63.9 mg, 65.0 uL, 3 equiv)

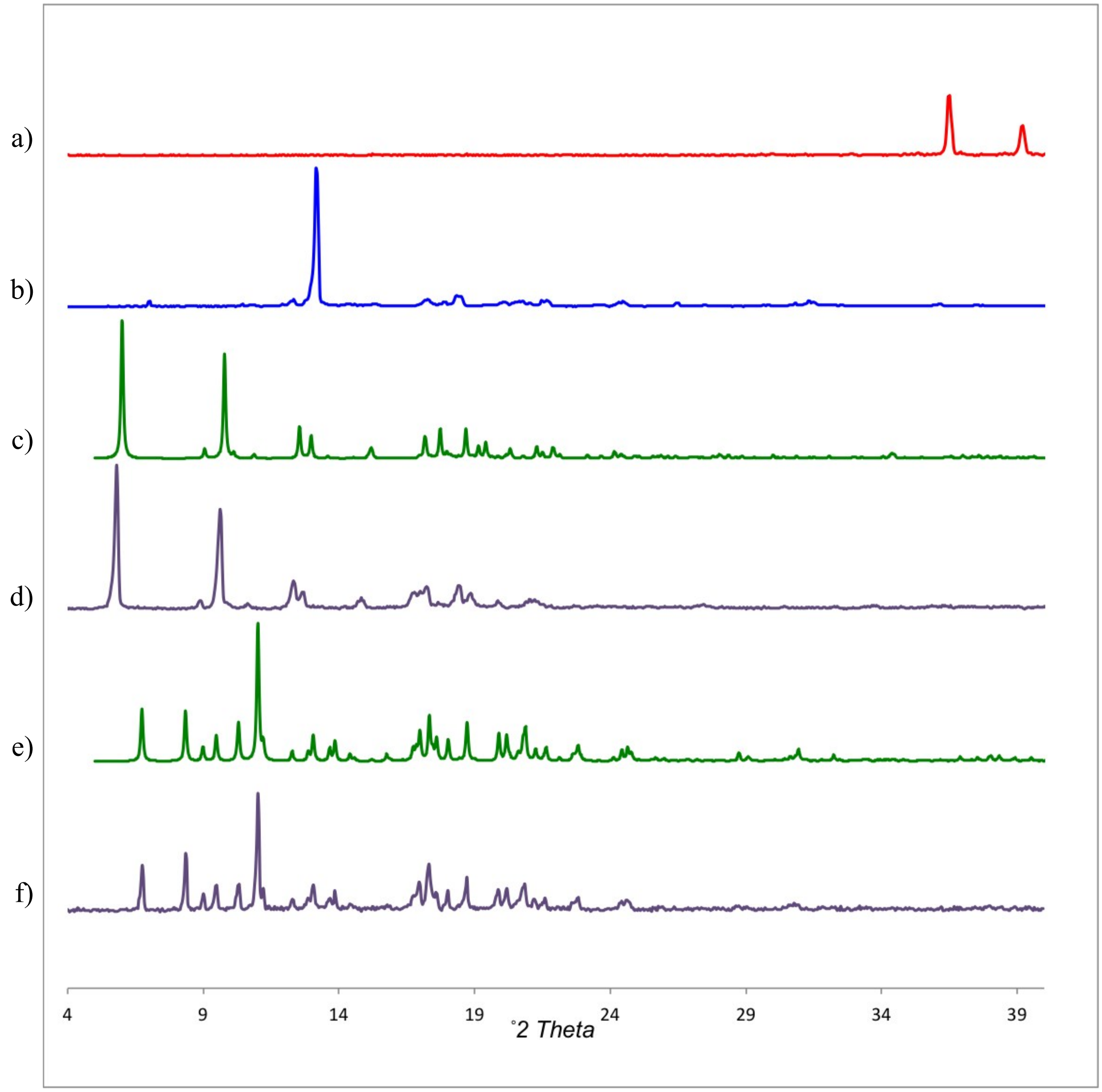

Figure S3. Powder X-ray diffraction data for the synthesis of **3** and **4** from Zn: a) Zn; b) **1**; c) Simulated pattern of **3**; d) Crude milled product of Zn (1 equiv), **1** (2 equiv), **Py** (3 equiv); e) Simulated pattern of **4**; f) Crude milled product of Zn (1 equiv), **1** (2 equiv), **Py** (1 equiv)

1.3.2.2 From Zinc Oxide

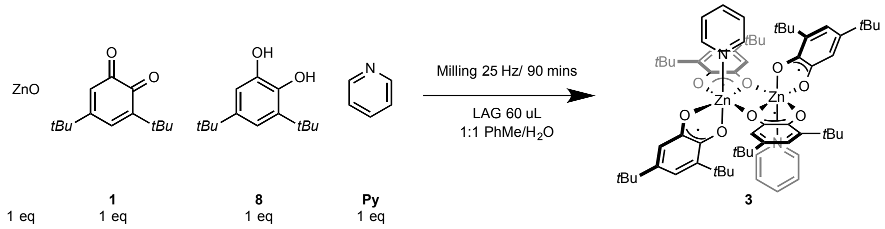

Scheme S5: ZnO (0.331 mmol, 26.7 mg, 1 equiv); **1** (0.331 mmol, 73.1 mg, 1 equiv); **8** (0.331 mmol, 73.7 mg, 1 equiv); **Py** (0.331 mmol, 26.2 mg, 26.7 uL, 1 equiv)

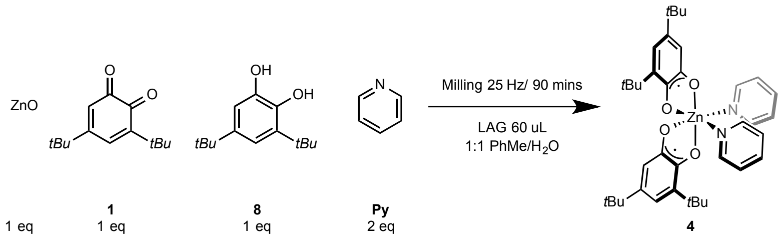

Scheme S6: ZnO (0.293 mmol, 23.9 mg, 1 equiv); **1** (0.293 mmol, 64.6 mg, 1 equiv); **8** (0.293 mmol, 65.2 mg, 1 equiv); **Py** (0.586 mmol, 46.4 mg, 47.2 uL, 2 equiv)

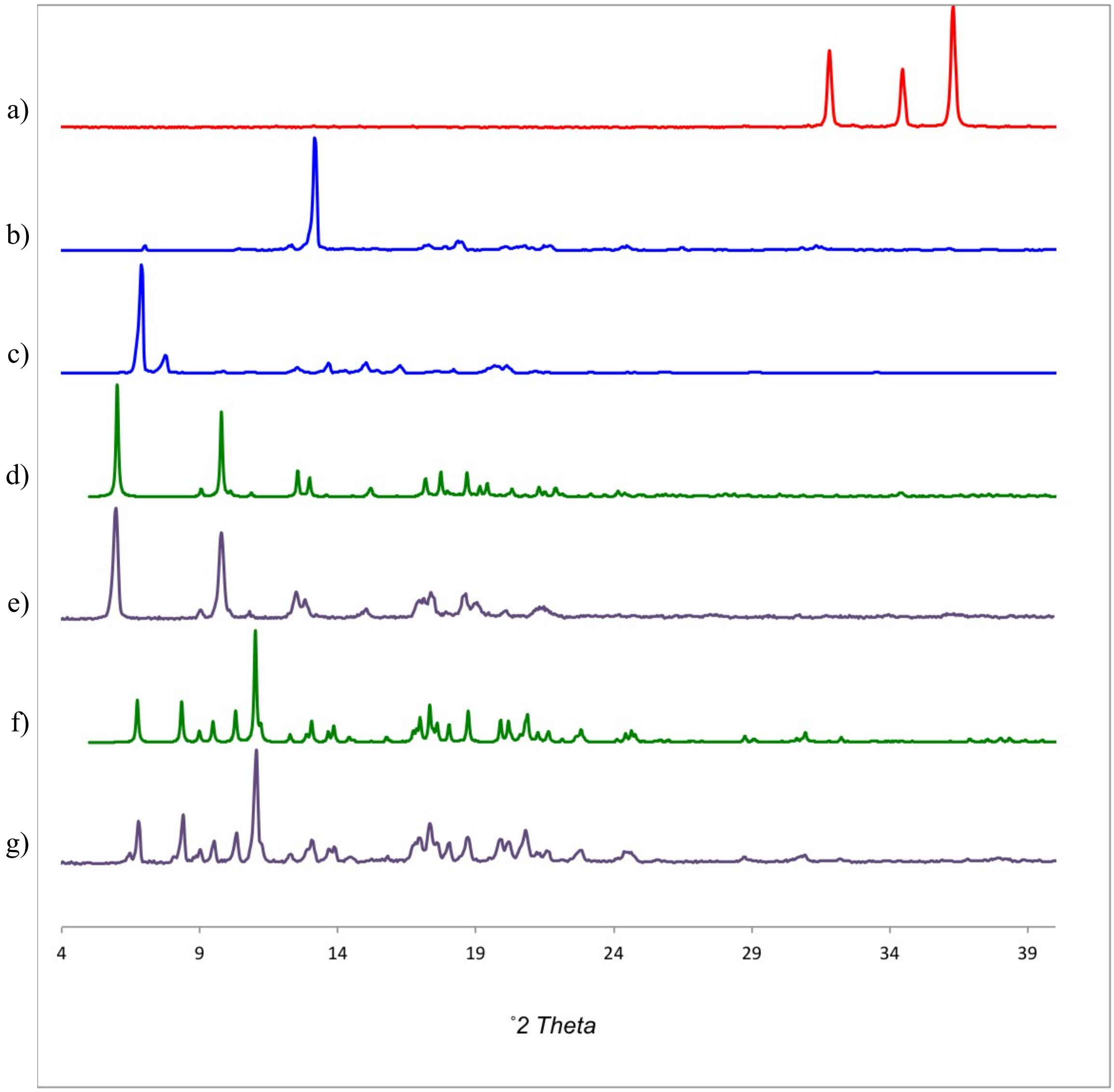

Figure S4. Powder X-ray diffraction data for the synthesis of **3** and **4** from ZnO: a) ZnO; b) **1**; c) **8**; d) Simulated pattern of **3**; e) Crude milled product of ZnO (1 equiv), **1** (1 equiv), **8** (1 equiv), **Py** (1 equiv); f) Simulated pattern of **4**; g) Crude milled product of ZnO (1 equiv), **1** (1 equiv), **8** (1 equiv), **Py** (2 equiv);

1.3.2.3 From complex **2**

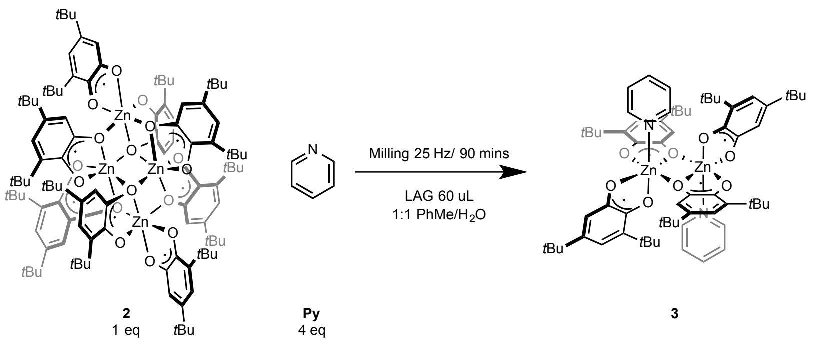

Scheme S7: **2** (0.0854 mmol, 173.0 mg, 1 equiv); **Py** (0.342 mmol, 27.0 mg, 27.5 uL, 4 equiv)

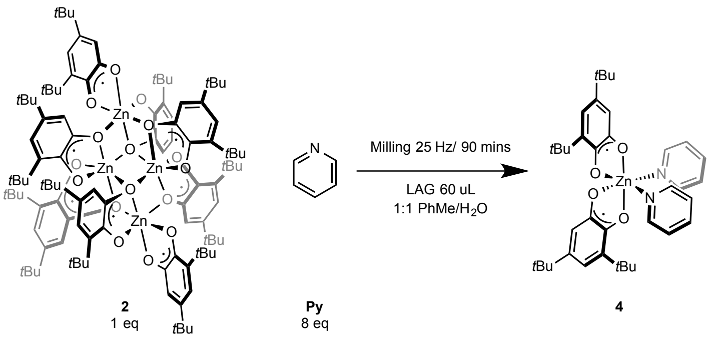

Scheme S8: **2** (0.0753 mmol, 152.4 mg, 1 equiv); **Py** (0.602 mmol, 47.6 mg, 48.5 uL, 8 equiv)

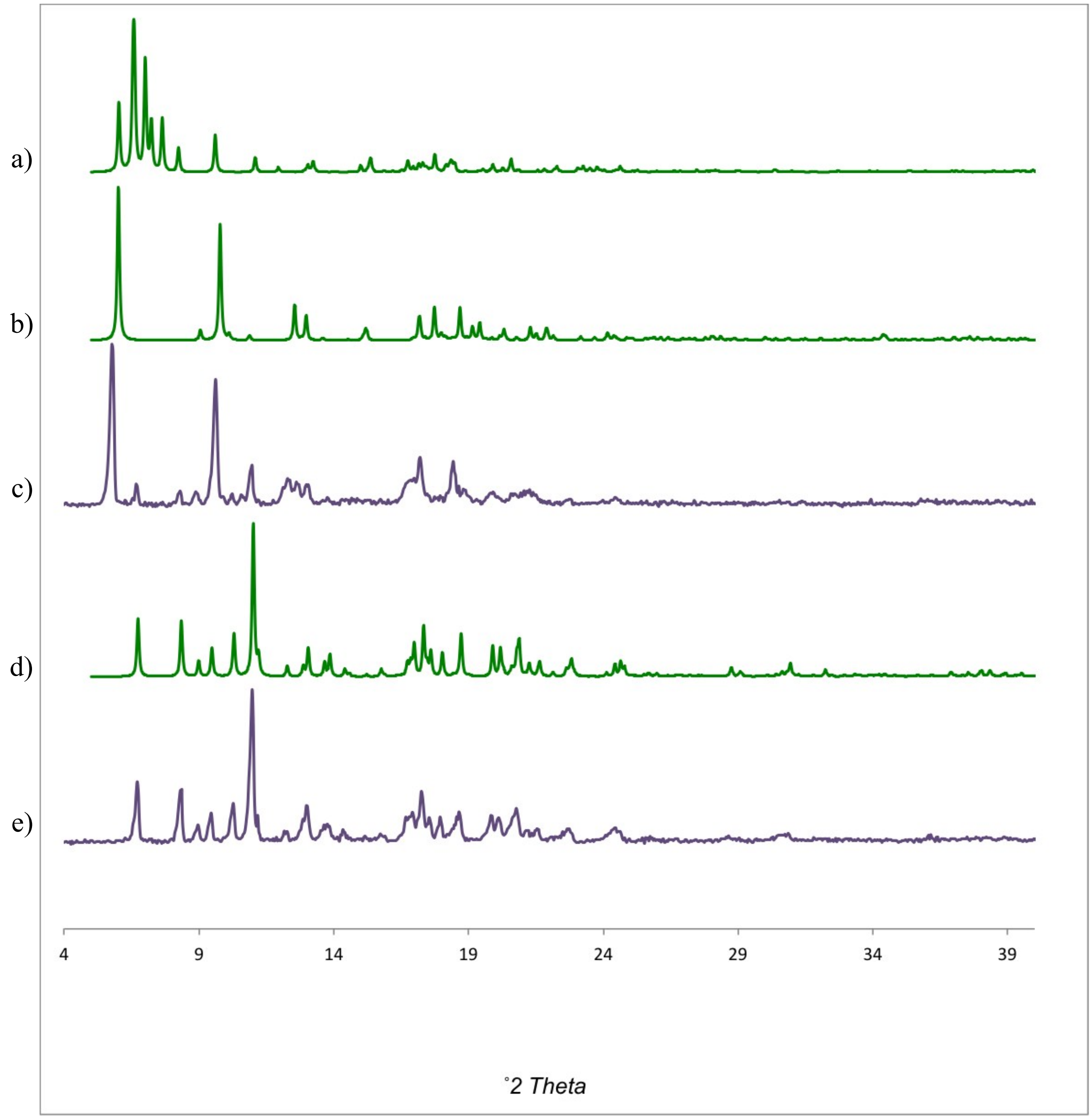

Figure S5. Powder X-ray diffraction data for the synthesis of **3** and **4** from complex **2**: a) Simulated pattern of $[Co^{II}(3,5\text{-dtbsq})_2]_4$(benzene) tetranuclear complex TBSQCO from CCDC database; b) Simulated pattern of **3**; c) Crude milled product of **2** (1 equiv), **Py** (4 equiv); d) Simulated pattern of **4;** e) Crude milled product of **2** (1 equiv), **Py** (8 equiv)

*1.3.3 Synthesis of Zn(3,5-dtbsq)$_2$(N-Methyl Imidazole)$_1$ (**5**) and Zn(3,5-dtbsq)$_2$(N-Methyl Imidazole)$_2$ (**6**)*

1.3.3.1 From Zinc powder

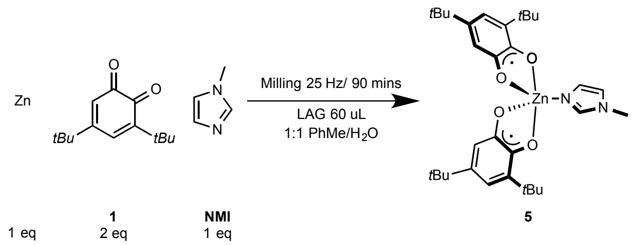

Scheme S9: Zn (0.340 mmol, 22.2 mg, 1 equiv); **1** (0.680 mmol, 150 mg, 2 equiv); N-Methyl Imidazole (**NMI**) (0.340 mmol, 27.9 mg, 27.1 uL, 1 equiv)

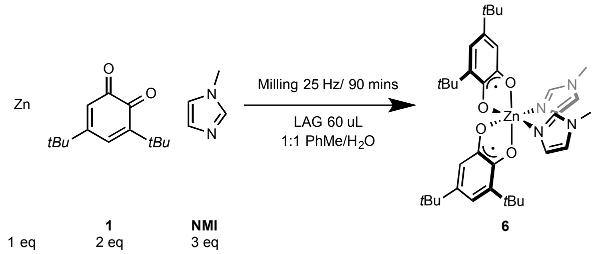

Scheme S10: Zn (0.266 mmol, 17.4 mg, 1 equiv); **1** (0.532 mmol, 117.1 mg, 2 equiv); **NMI** (0.798 mmol, 65.5 mg, 63.6 uL, 3 equiv)

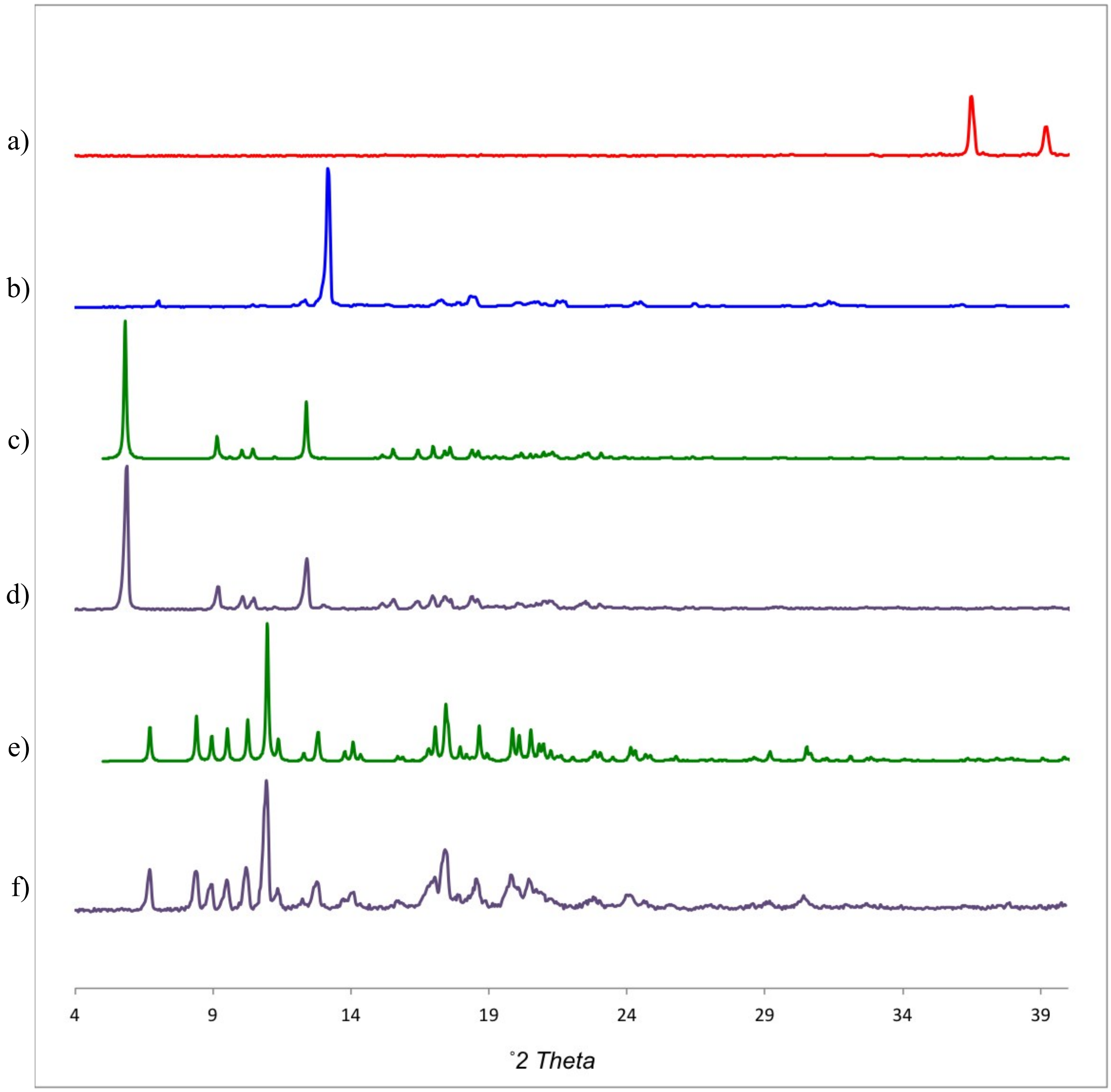

Figure S6. Powder X-ray diffraction data for the synthesis of **5** and **6** from Zn: a) Zn; b) **1**; c) Simulated pattern of **5**; d) Crude milled product of Zn (1 equiv), **1** (2 equiv), **NMI** (1 equiv); e) Simulated pattern of **6**; f) Crude milled product of Zn (1 equiv), **1** (2 equiv), **NMI** (3 equiv);

1.3.3.2 From Zinc Oxide

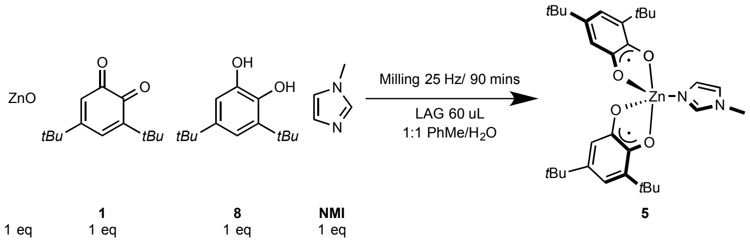

Scheme S11: ZnO (0.330 mmol, 26.9 mg, 1 equiv); **1** (0.330 mmol, 72.7 mg, 1 equiv); **8** (0.330 mmol, 73.5 mg, 1 equiv) **NMI** (0.330 mmol, 27.1 mg, 26.3 uL, 1 equiv)

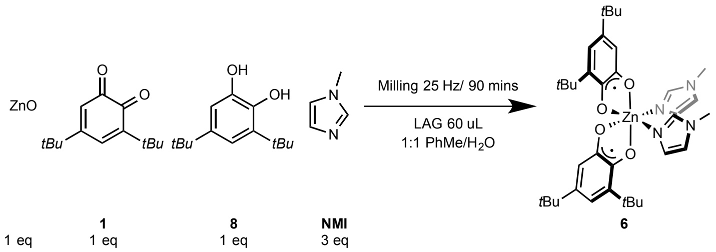

Scheme S12: ZnO (0.260 mmol, 21.1 mg, 1 equiv); **1** (0.260 mmol, 57.2 mg, 1 equiv); **8** (0.260 mmol, 57.7 mg, 1 equiv); **NMI** (0.779 mmol, 63.9 mg, 62.1 uL, 3 equiv)

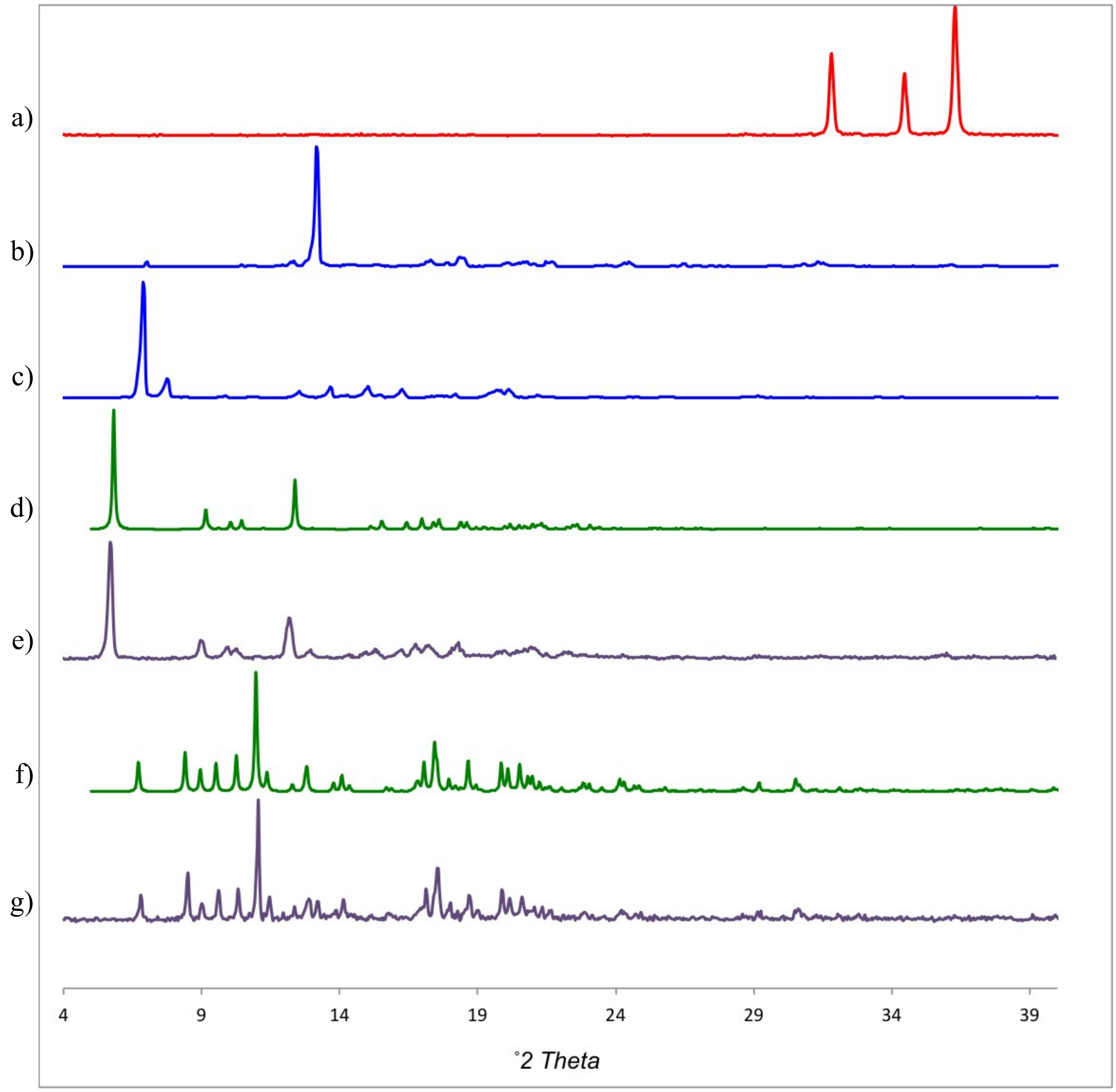

Figure S7. Powder X-ray diffraction data for the synthesis of **5** and **6** from ZnO: a) ZnO; b) **1**; c) **8**; d) Simulated pattern of **5**; e) Crude milled product of ZnO (1 equiv), **1** (1 equiv), **8** (1 equiv), **NMI** (1 equiv); f) Simulated pattern of **6**; g) Crude milled product of ZnO (1 equiv), **1** (1 equiv), **8** (1 equiv), **NMI** (3 equiv);

1.3.3.3 From complex **2**

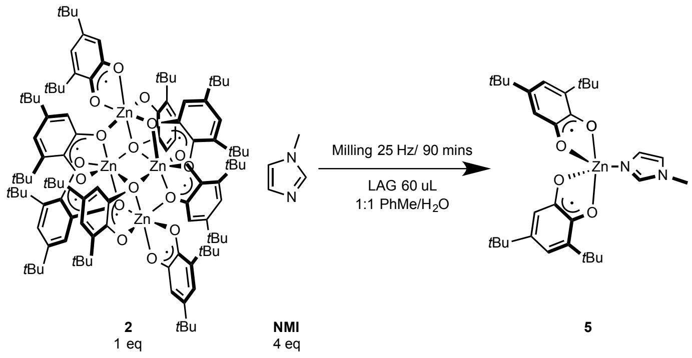

Scheme S13: **2** (0.0850 mmol, 172 mg, 1 equiv); **NMI** (0.340 mmol, 27.9 mg, 27.1 uL, 4 equiv)

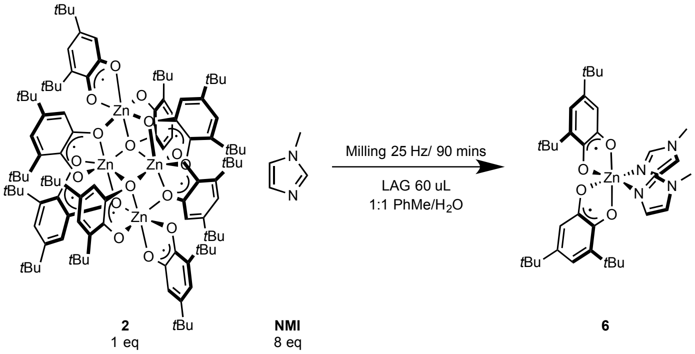

Scheme S14: **2** (0.0746 mmol, 151 mg, 1 equiv); **NMI** (0.597 mmol, 49.0 mg, 47.6 uL, 8 equiv)

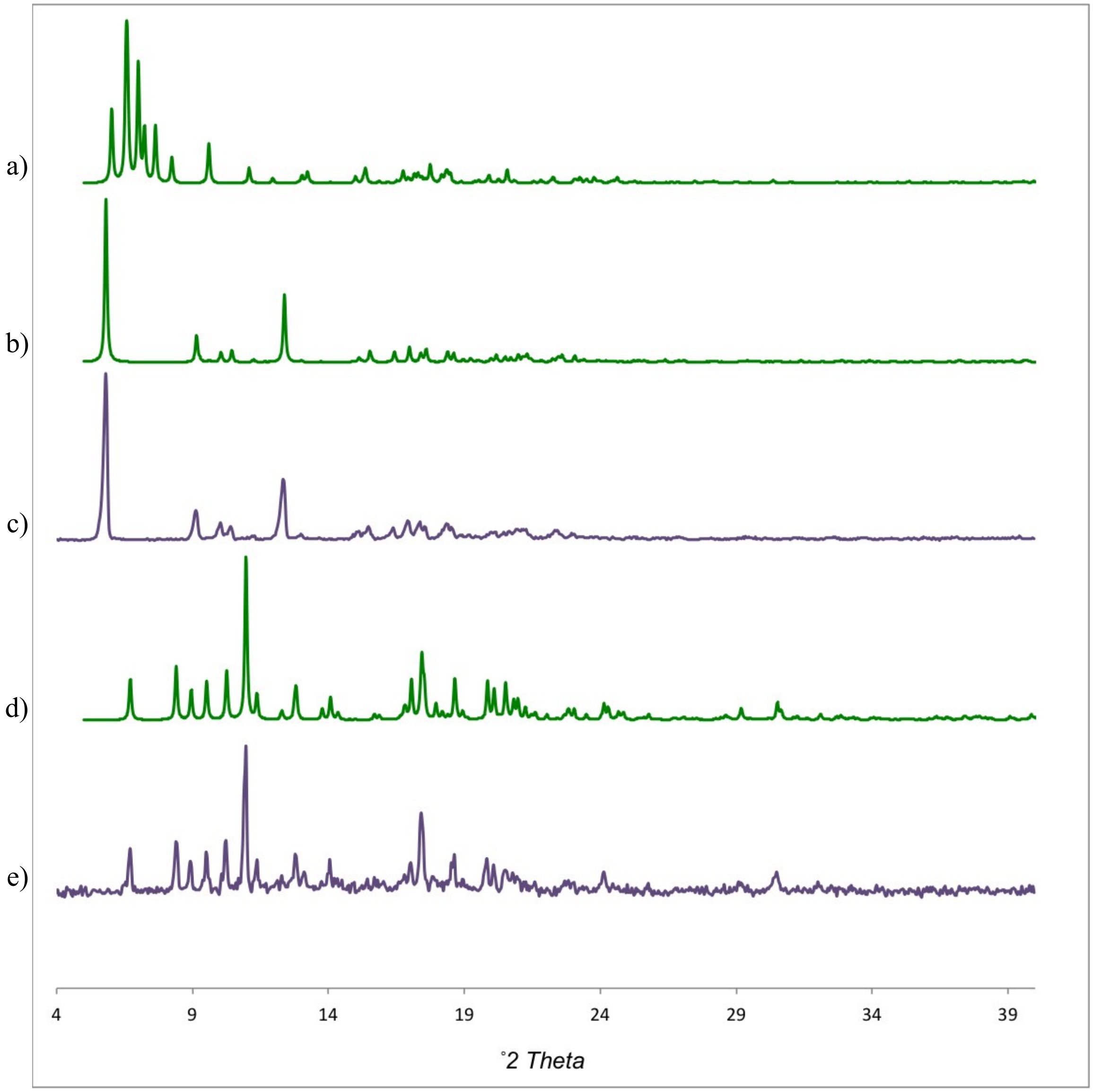

Figure S8. Powder X-ray diffraction data for the synthesis of **5** and **6** from complex **2**: a) Simulated pattern of $[Co^{II}(3,5\text{-dtbsq})_2]_4$(benzene) tetranuclear complex TBSQCO from CCDC database; b) Simulated pattern of **5**; c) Crude milled product of **2** (1 equiv), **NMI** (4 equiv); d) Simulated pattern of **6**; e) Crude milled product of **2** (1 equiv), **NMI** (8 equiv);

1.3.4 *Synthesis of Zn(3,5-dtbsq)$_2$(1,10-phenanthroline)* (**7**)

1.3.4.1 *From Zinc powder*

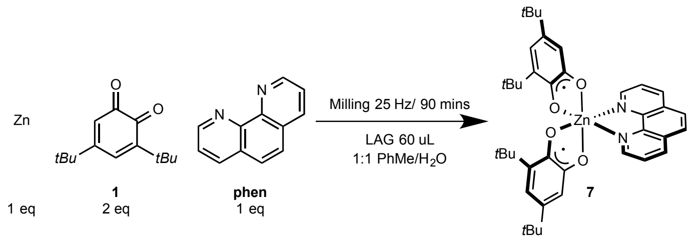

Scheme S15: Zn (0.284 mmol, 18.6 mg, 1 equiv); **1** (0.568 mmol, 125 mg, 2 equiv); 1,10-phenanthroline (**phen**) (0.284 mmol, 56.3 mg, 1 equiv)

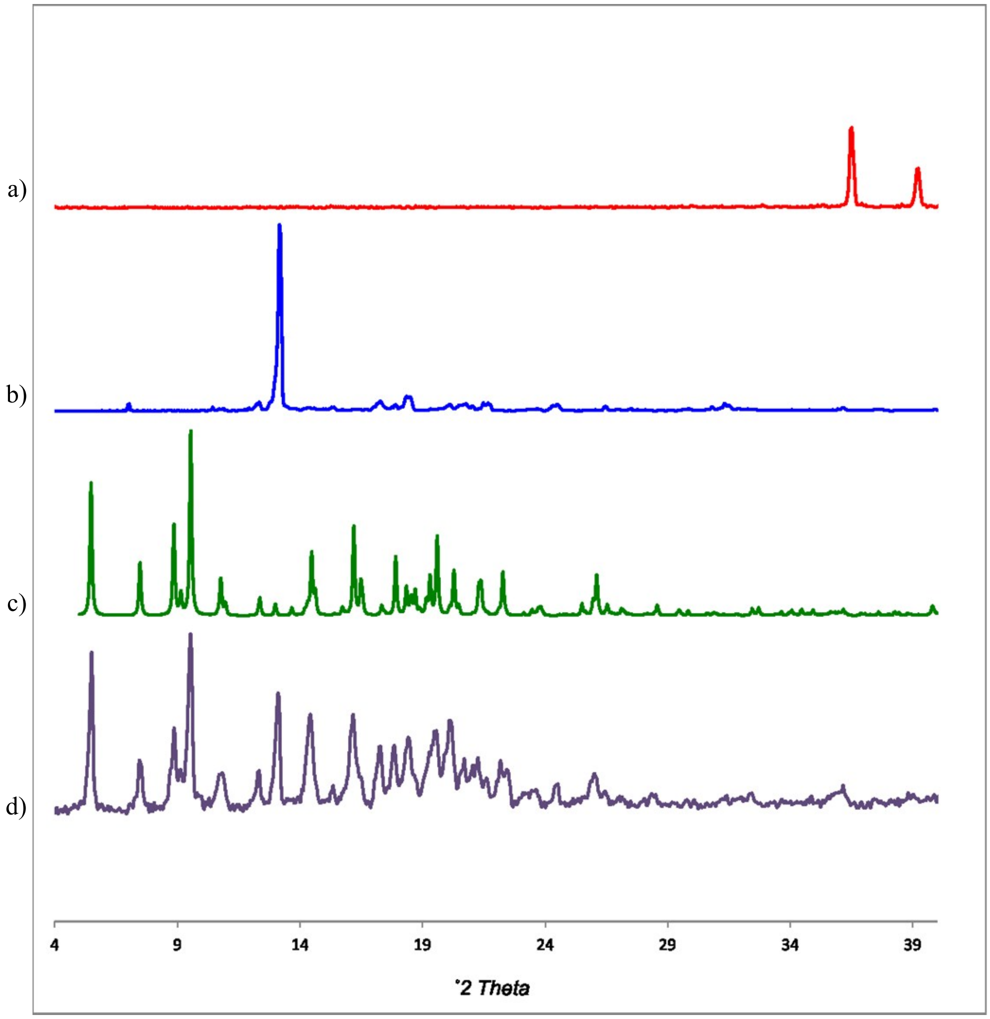

Figure S9. Powder X-ray diffraction data for the synthesis of **7** from Zn: a) Zn; b) **1**; c) Simulated pattern of **7**; d) Crude milled product of Zn (1 equiv), **1** (2 equiv), **phen** (1 equiv);

1.3.4.2 From Zinc Oxide

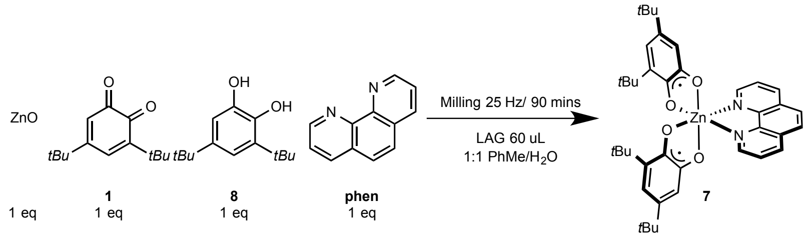

Scheme S16: ZnO (0.277 mmol, 22.5 mg, 1 equiv); **1** (0.277 mmol, 61.0 mg, 1 equiv); **8** (0.277 mmol, 61.6 mg, 1 equiv); **phen** (0.277 mmol, 54.9 mg, 1 equiv)

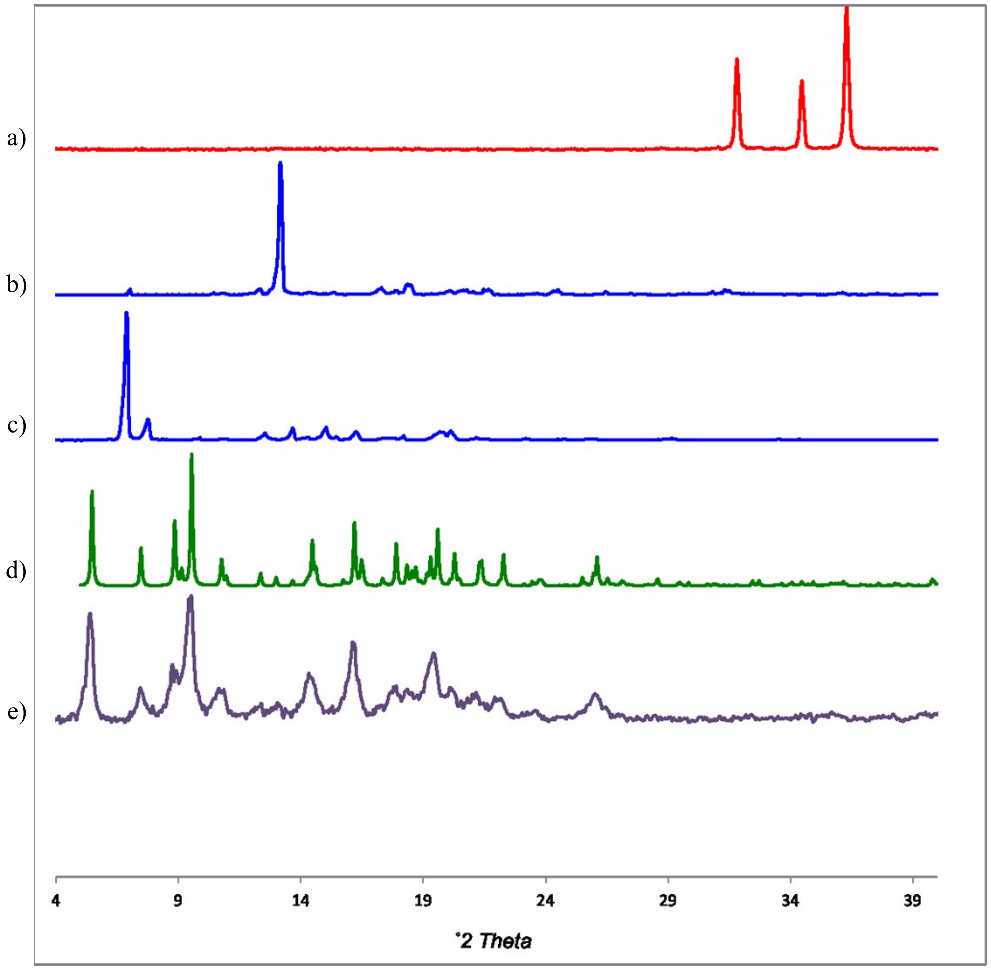

Figure S10. Powder X-ray diffraction data for the synthesis of **7** from ZnO: a) ZnO; b) **1**; c) **8**; d) Simulated pattern of **7**; e) Crude milled product of ZnO (1 equiv), **1** (1 equiv), **8** (1 equiv), **phen** (1 equiv);

1.3.4.3 From complex **2**

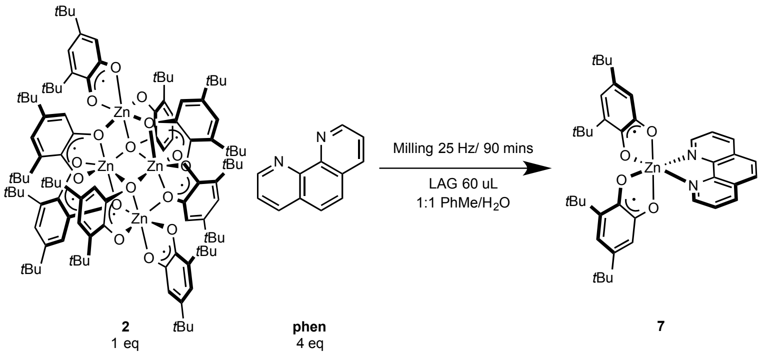

Scheme S17: **2** (0.0710 mmol, 144 mg, 1 equiv); **phen** (0.284 mmol, 56.3 mg, 4 equiv)

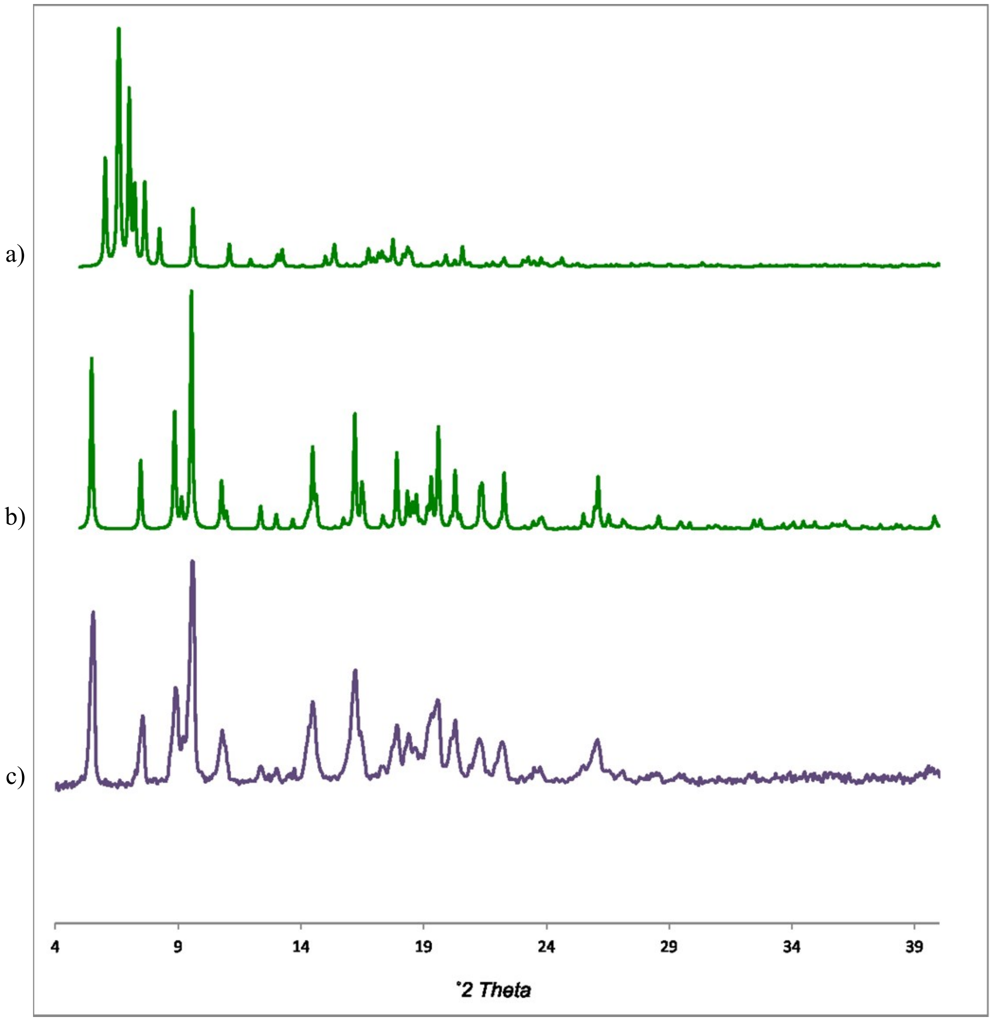

Figure S11. Powder X-ray diffraction data for the synthesis of **7** from complex **2**: a) Simulated pattern of $[Co^{II}(3,5\text{-dtbsq})_2]_4$(benzene) tetranuclear complex TBSQCO from CCDC database; b) Simulated pattern of **7**; c) Crude milled product of **2** (1 equiv), **phen** (4 equiv);

1.3.5 *Synthesis of and Zn_2(3,5-dtbsq)$_4$(1,4-bis(2-(pyridin-4-yl)vinyl)benzene)* (**9**) *and Zn(3,5-dtbsq)$_2$(1,4-bis(2-(pyridin-4-yl)vinyl)benzene)* (**10**)

1.3.5.1 *From Zinc powder*

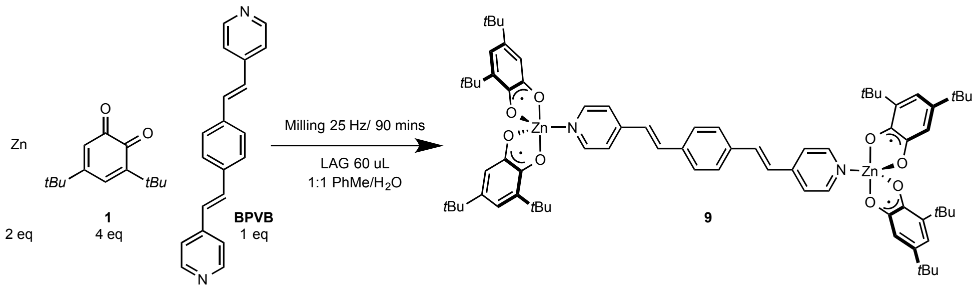

Scheme S18: Zn (0.309 mmol, 20.2 mg, 2 equiv); **1** (0.617 mmol, 136.0 mg, 4 equiv); 1,4-bis(2-(pyridin-4-yl)vinyl) benzene (**BPVB**) (0.154 mmol, 43.9 mg, 1 equiv)
.

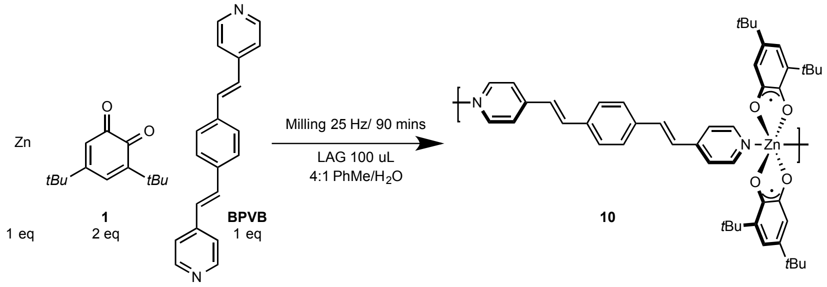

Scheme S19: Zn (0.253 mmol, 16.5 mg, 1 equiv); **1** (0.506 mmol, 111.5 mg, 2 equiv), **BPVB** (0.253 mmol, 72.0 mg, 1 equiv) LAG conditions were 100 uL of a 4:1 PhMe/H_2O mixture.

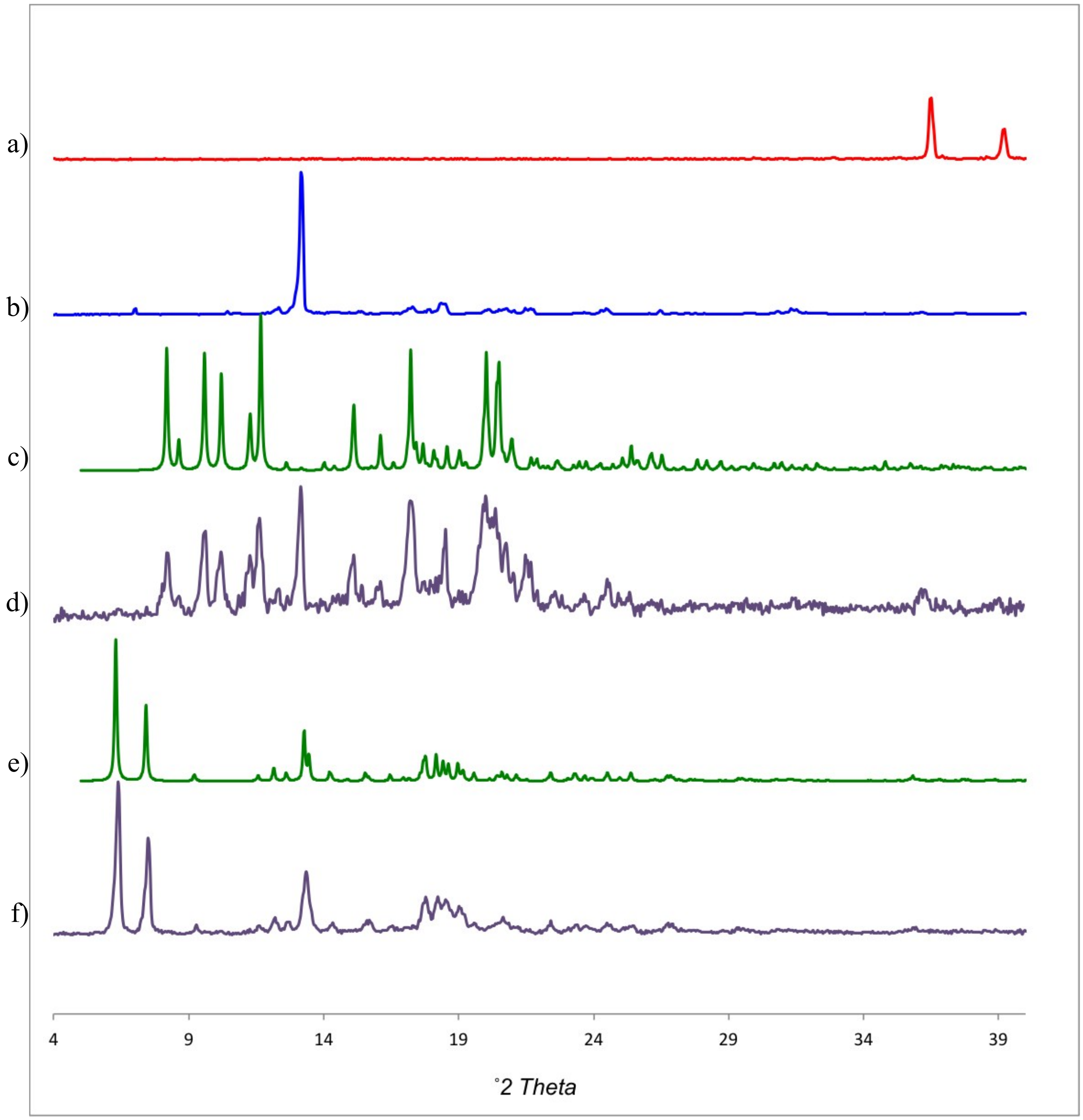

Figure S12. Powder X-ray diffraction data for the synthesis of **9** and **10** from Zn: a) Zn; b) **1**; c) Simulated pattern of **9**; d) Crude milled product of Zn (2 equiv), **1** (4 equiv), **BPVB** (1 equiv); e) Simulated pattern of **10**; f) Crude milled product of Zn (1 equiv), **1** (2 equiv), **BPVB** (1 equiv) LAG 100 uL 4:1 PhMe/H_2O;

1.3.5.2 From Zinc Oxide

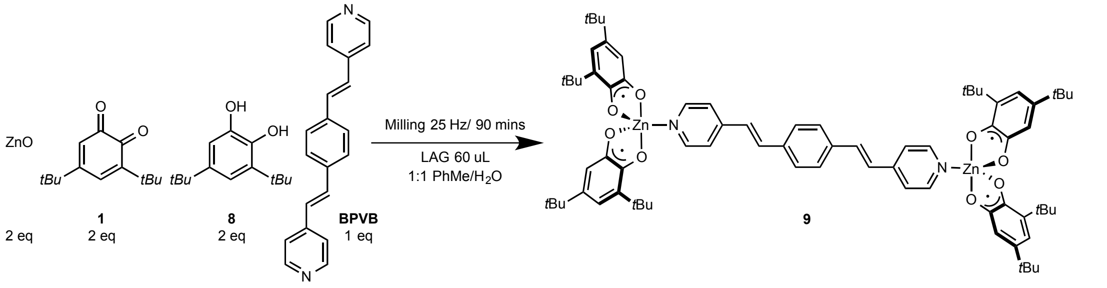

Scheme S20: ZnO (0.285 mmol, 23.2 mg, 2 equiv); **1** (0.285 mmol, 62.8 mg, 2 equiv); **8** (0.142 mmol, 63.4 mg, 2 equiv); **BPVB** (0.154 mmol, 40.5 mg, 1 equiv)

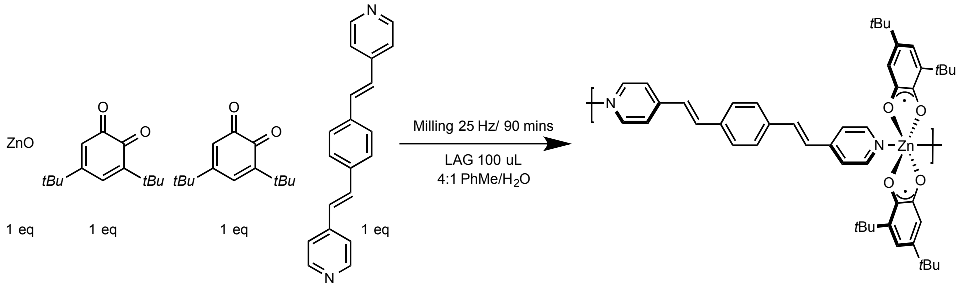

Scheme S21: ZnO (0.247 mmol, 20.1 mg, 1 equiv); **1** (0.247 mmol, 54.5 mg, 1 equiv); **8** (0.247 mmol, 55.0 mg, 1 equiv); **BPVB** (0.247 mmol, 70.4 mg, 1 equiv) LAG conditions were 100 uL of a 4:1 PhMe/H_2O mixture.

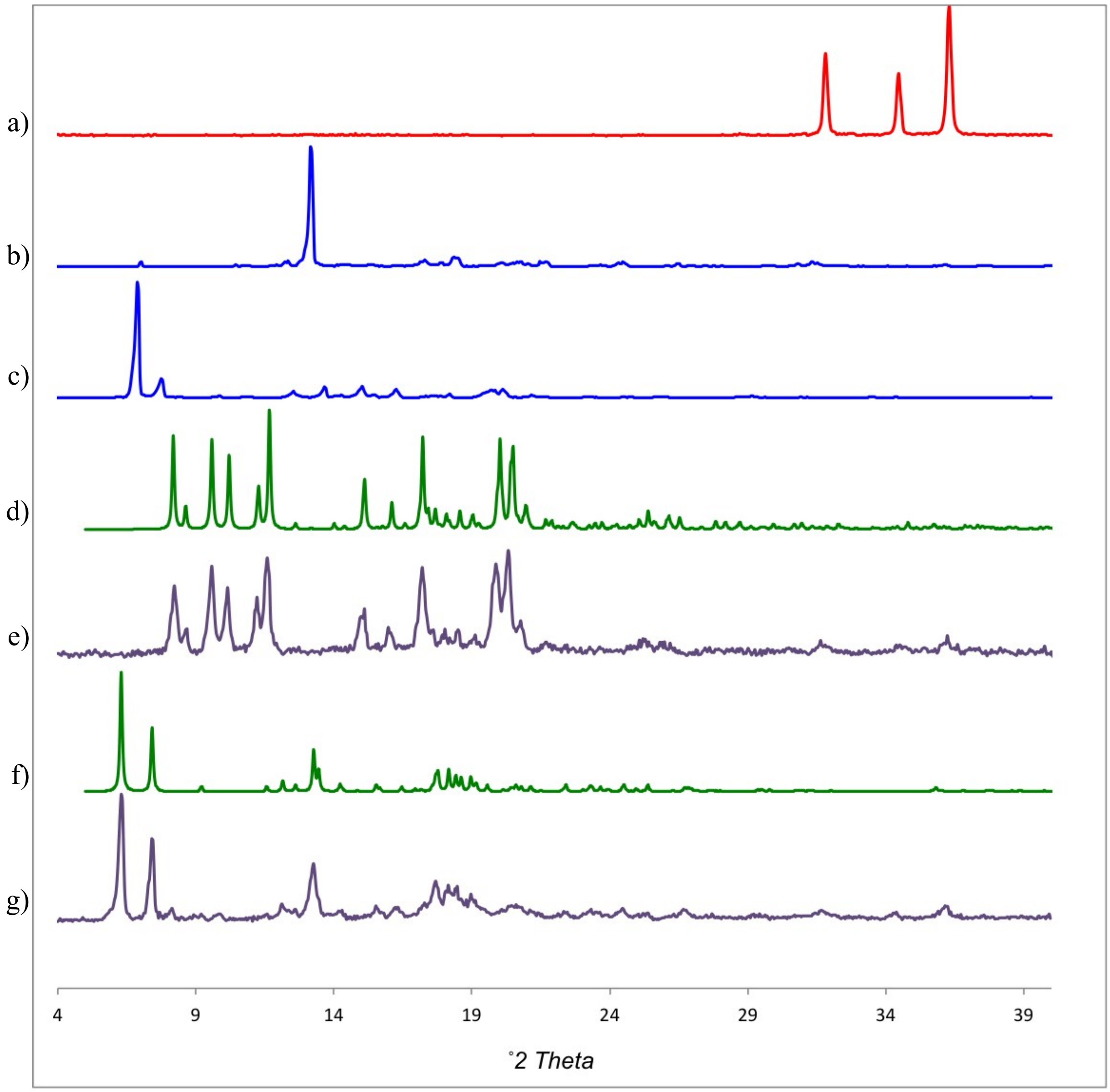

Figure S13. Powder X-ray diffraction data for the synthesis of **9** and **10** from ZnO: a) ZnO; b) **1**; c) **8**; d) Simulated pattern of **9**; e) Crude milled product of ZnO (2 equiv), **1** (2 equiv), **8** (2 equiv), **BPVB** (1 equiv); f) Simulated pattern of **10**; g) Crude milled product of Zinc Oxide (1 equiv), **1** (1 equiv), **8** (1 equiv), **BPVB** (1 equiv), LAG 100 uL 4:1 PhMe/H_2O;

1.3.5.3 *From complex* **2**

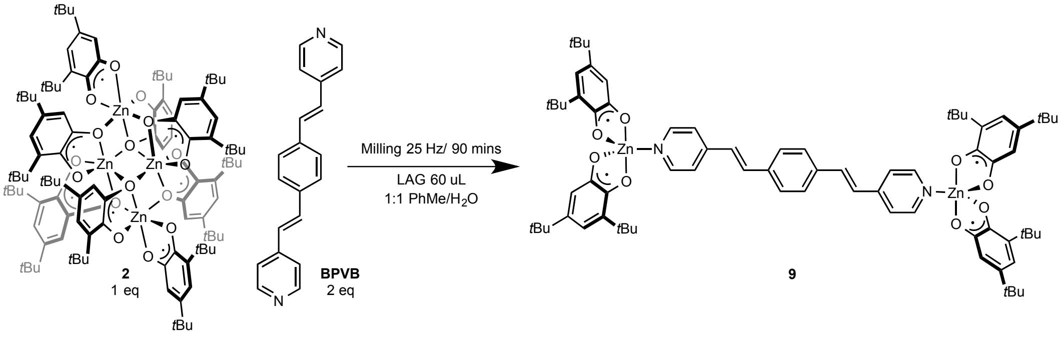

Scheme S22: **2** (0.0771 mmol, 156.1 mg, 1 equiv); **BPVB** (0.154 mmol, 43.9 mg, 2 equiv)

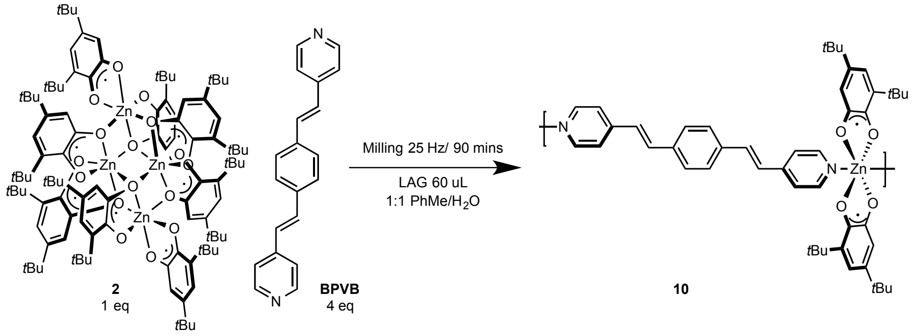

Scheme S23: **2** (0.0633 mmol, 128.0 mg, 1 equiv); **BPVB** (0.253 mmol, 72.0 mg, 4 equiv) LAG conditions were 100 uL of a 1:1 PhMe/H_2O mixture.

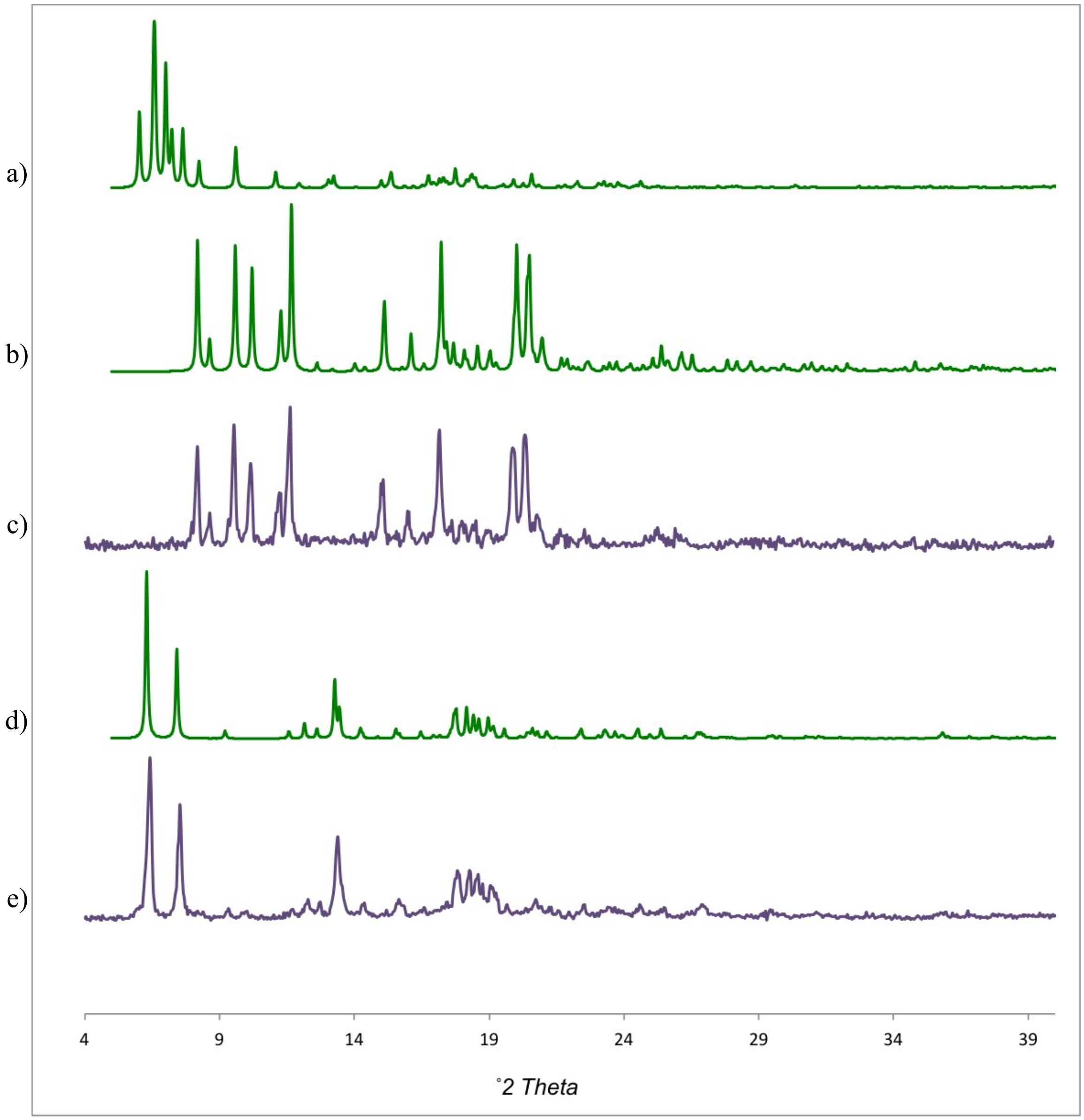

Figure S14. Powder X-ray diffraction data for the synthesis of **9** and **10** from complex **2**: a) Simulated pattern of $[Co^{II}(3,5\text{-dtbsq})_2]_4$(benzene) tetranuclear complex TBSQCO from CCDC database; b) Simulated pattern of **9**; c) Crude milled product of **2** (1 equiv), **BPVB** (2 equiv); d) Simulated pattern of **10**; e) Crude milled product of **2** (1 equiv), **BPVB** (4 equiv) LAG 100 uL 4:1 PhMe/H_2O;

1.3.6 Synthesis of Zn$_3$(3,5-dtbsq)$_6$(1,3,5-tris(2-(pyridin-4-yl)vinyl)benzene) (**11**)

1.3.6.1 From Zinc powder

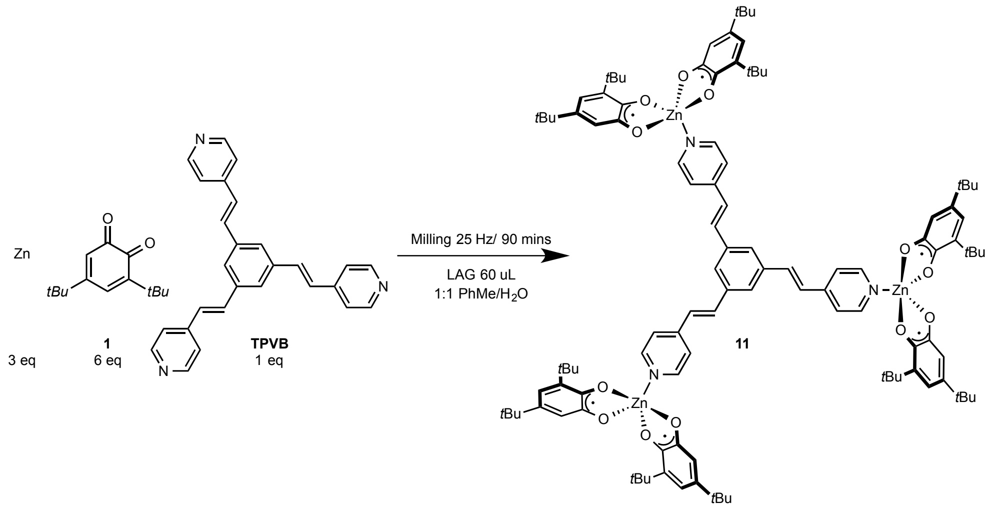

Scheme S24: Zn (0.315 mmol, 20.6 mg, 3 equiv); **1** (0.630 mmol, 139 mg, 6 equiv); 1,3,5-tris(2-(pyridin-4-yl)vinyl)benzene (**TPVB**) (0.105 mmol, 40.7 mg, 1 equiv)

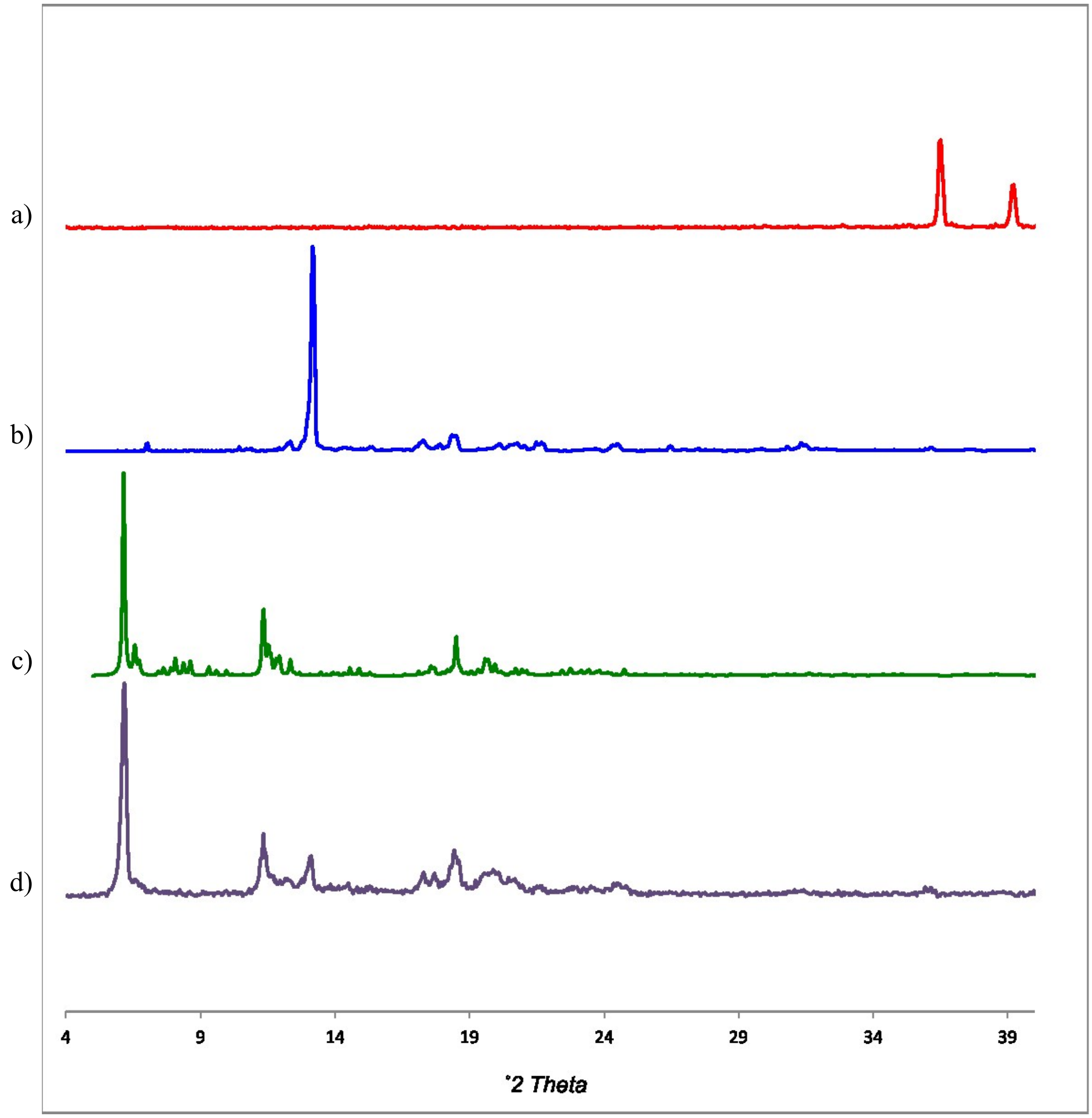

Figure S15. Powder X-ray diffraction data for the synthesis of **11** from Zn: a) Zn; b) **1**; c) Simulated pattern of **11**; d) Crude milled product of Zn (3 equiv), **1** (6 equiv), **TPVB** (1 equiv);

1.3.6.2 From Zinc Oxide

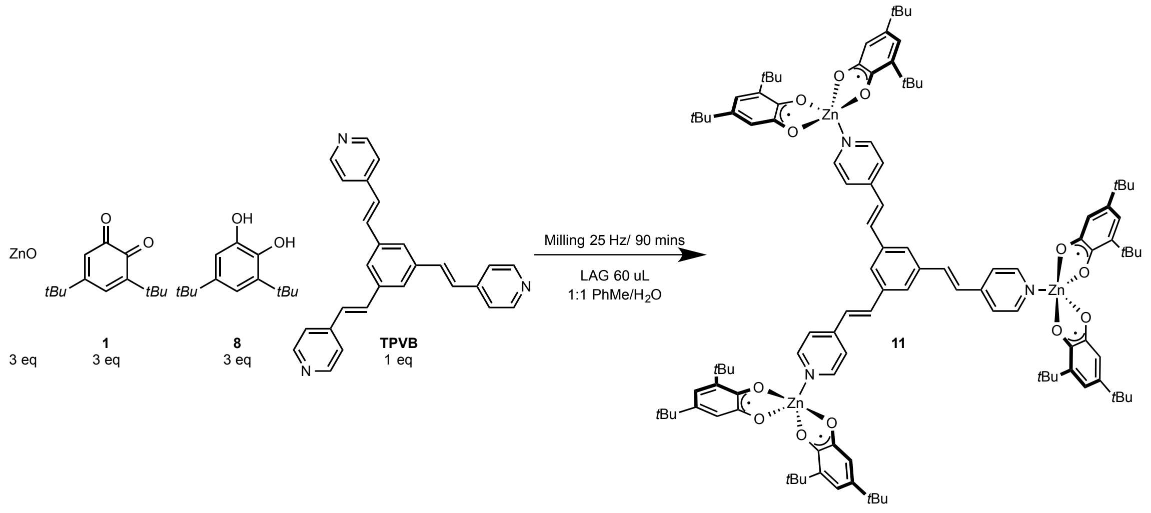

Scheme S25: ZnO (0.306 mmol, 24.9 mg, 3 equiv); **1** (0.306 mmol, 67.5 mg, 3 equiv); **8** (0.306 mmol, 68.1 mg, 3 equiv); **TPVB** (0.102 mmol, 39.5 mg, 1 equiv).

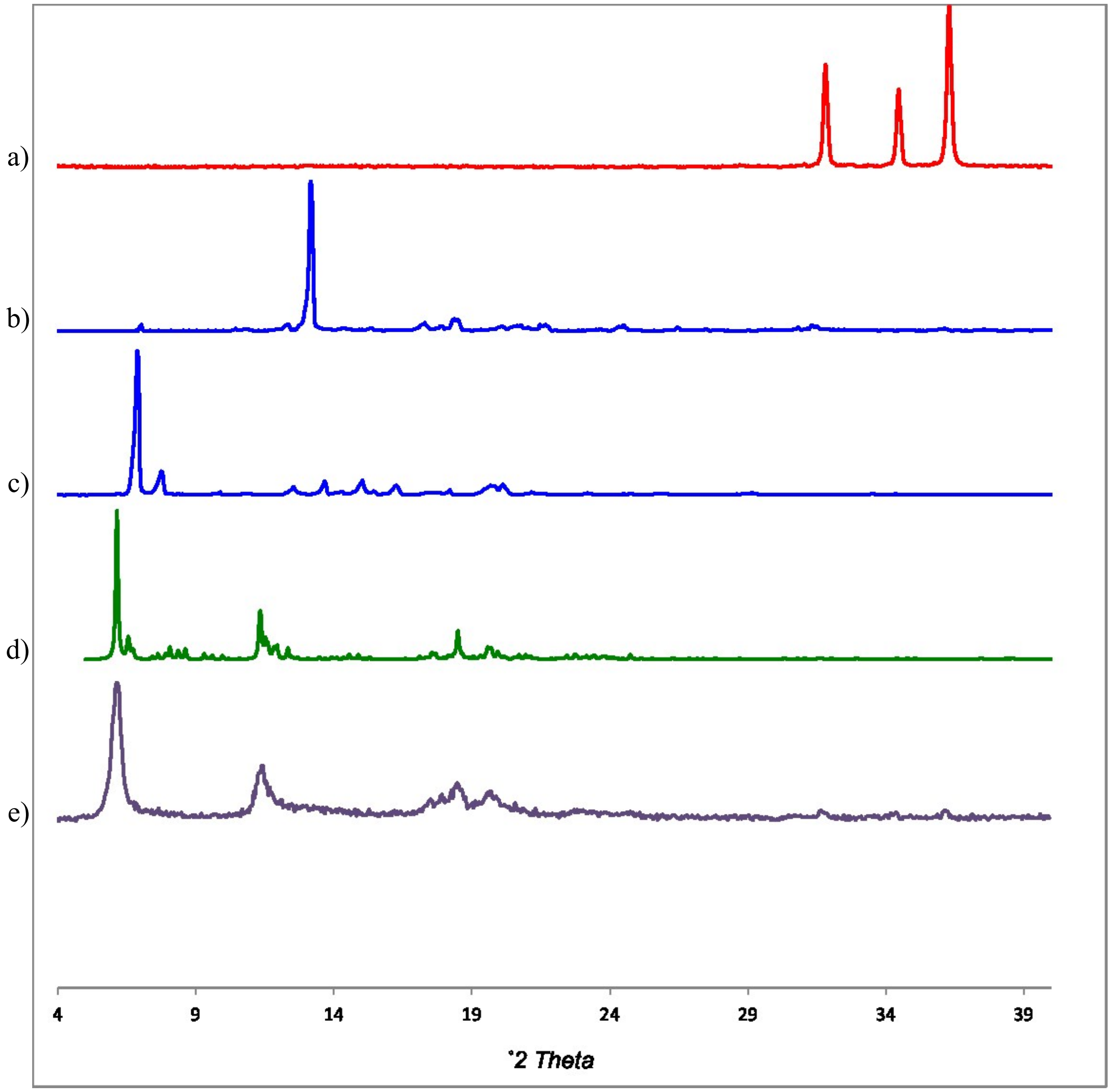

Figure S16. Powder X-ray diffraction data for the synthesis of **11** from ZnO: a) ZnO; b) **1**; c) **8**; d) Simulated pattern of **11**; e) Crude milled product of ZnO (3 equiv), **1** (3 equiv), **8** (3 equiv), **TPVB** (1 equiv);

1.3.6.3 From complex **2**

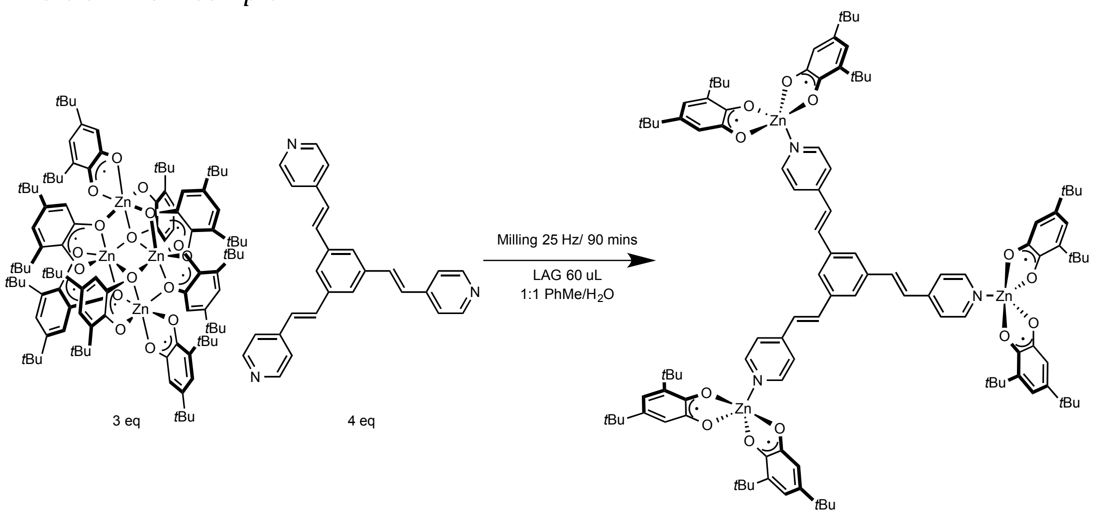

Scheme S25: **2** (0.0787 mmol, 159 mg, 3 equiv); **TPVB** (0.105 mmol, 40.7 mg, 4 equiv).

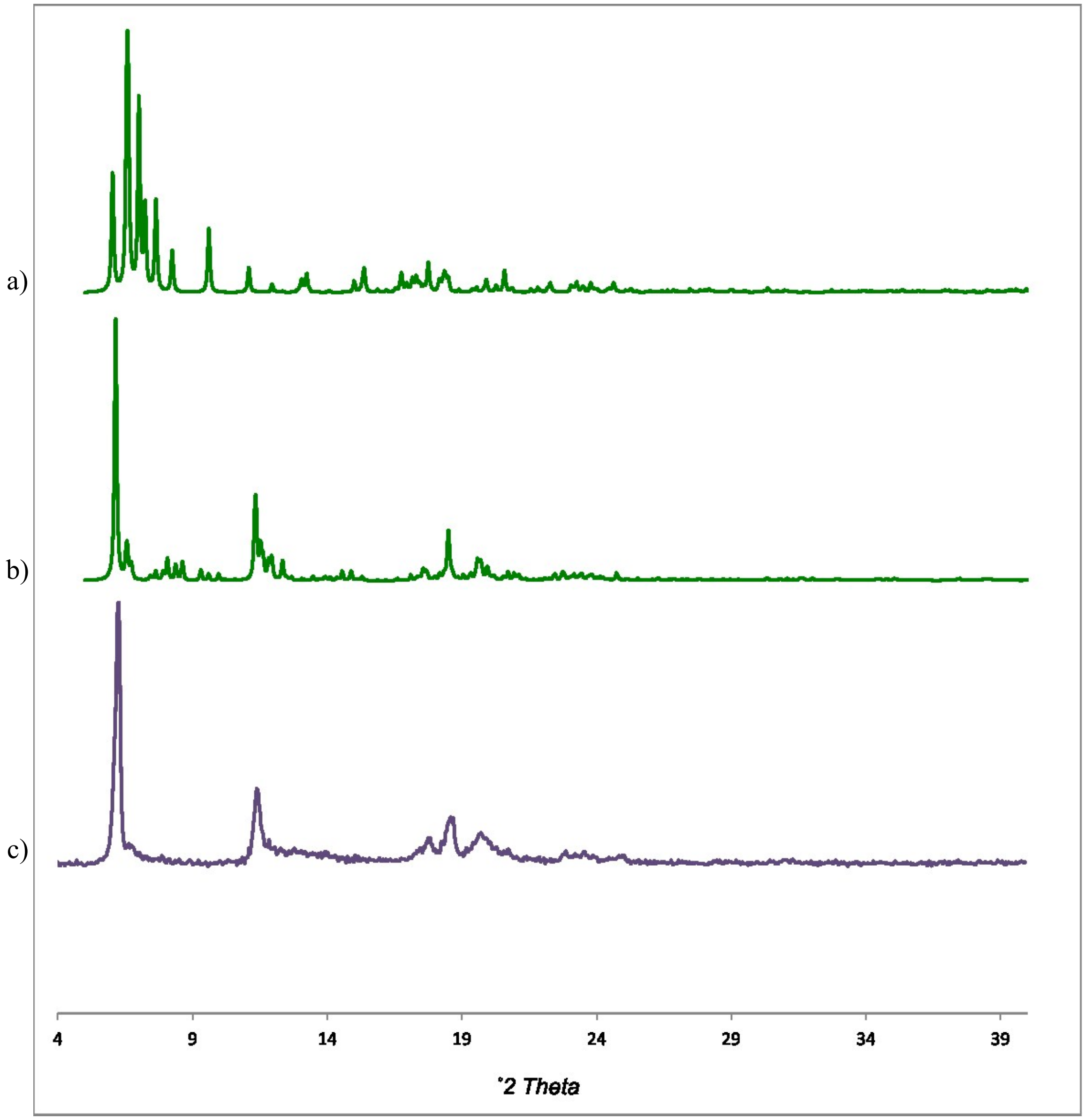

Figure S17. Powder X-ray diffraction data for the synthesis of **11** from complex **2**: a) Simulated pattern of [Co^{II}(3,5-dtbsq)$_2$]$_4$(benzene) tetranuclear complex TBSQCO from CCDC database; b) Simulated pattern of **11**; c) Crude milled product of **2** (3 equiv), **TPVB** (4 equiv);

1.3.7 *Ligand Interconversion*

1.3.7.1 *Interconversion of pyridine complexes **3** and **4***

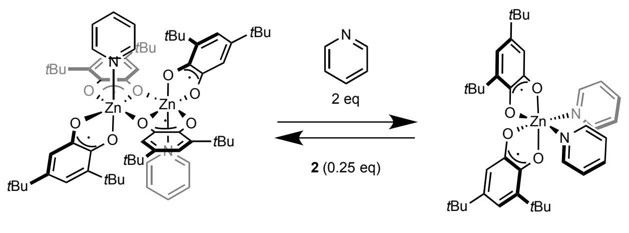

Scheme S25: (**3** to **4**)**: 3** (0.151 mmol, 176.2 mg, 1 equiv); **Py** (0.301 mmol, 23.8 mg, 24.3 uL, 2 equiv); (**4** to **3**)**: 4** (0.171 mmol, 113.5 mg, 1 equiv); **2** (0.0427 mmol, 86.48 mg, 0.25 equiv).

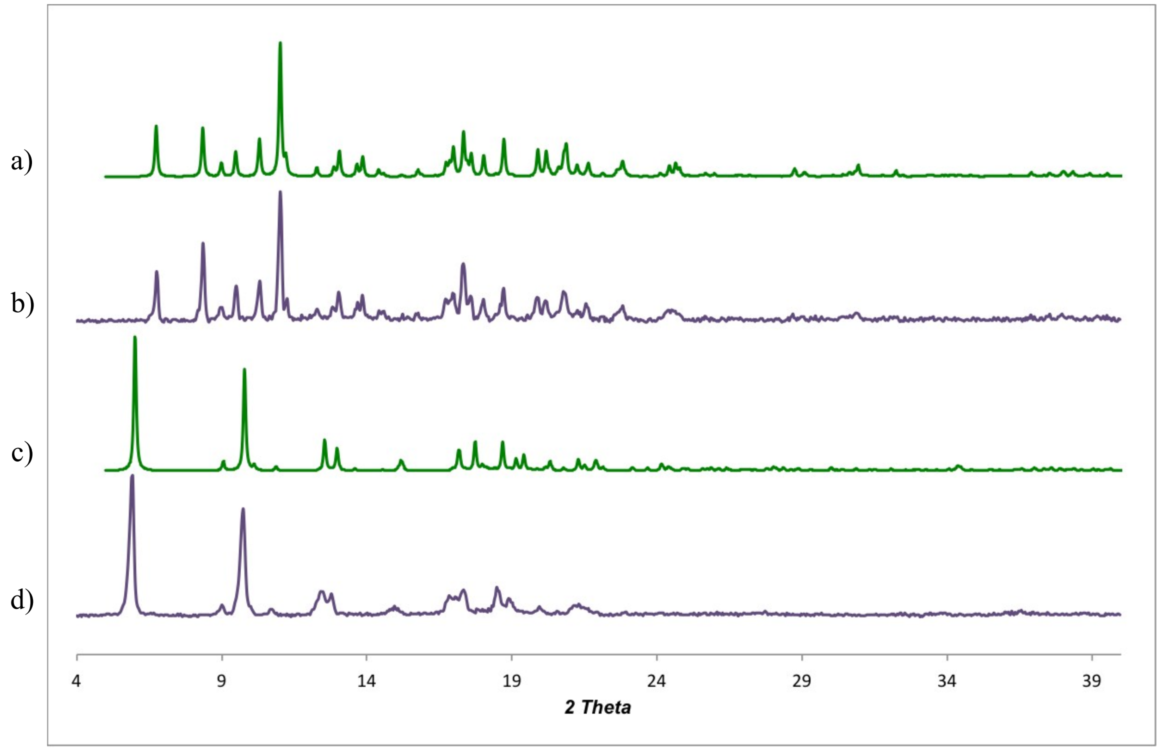

Figure S18. Powder X-ray diffraction data for the interconversion of complexes **3** and **4**: a) Simulated pattern of **4**; b) Crude milled product of **3** (1 equiv), **Py** (2 equiv); c) Simulated pattern of **3**; d) Crude milled product of **4** (1 equiv), **2** (0.25 equiv)

1.3.7.2 Interconversion of N-methyl imidazole complexes **5** *and* **6**

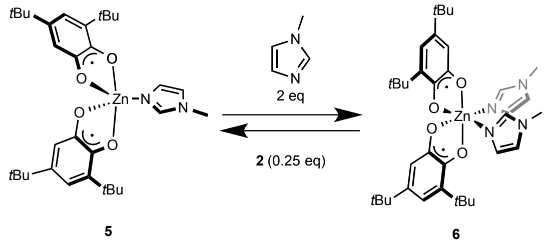

Scheme S26: (**5** to **6**)**: 5** (0.298 mmol, 175.5 mg, 1 equiv), **NMI** (0.596 mmol, 49.0 mg, 47.6 uL, 2 equiv) (**6** to **5**)**: 6** (0.170 mmol, 114.0 mg, 1 equiv), **2** (0.0425 mmol, 86.00 mg, 0.25 equiv)

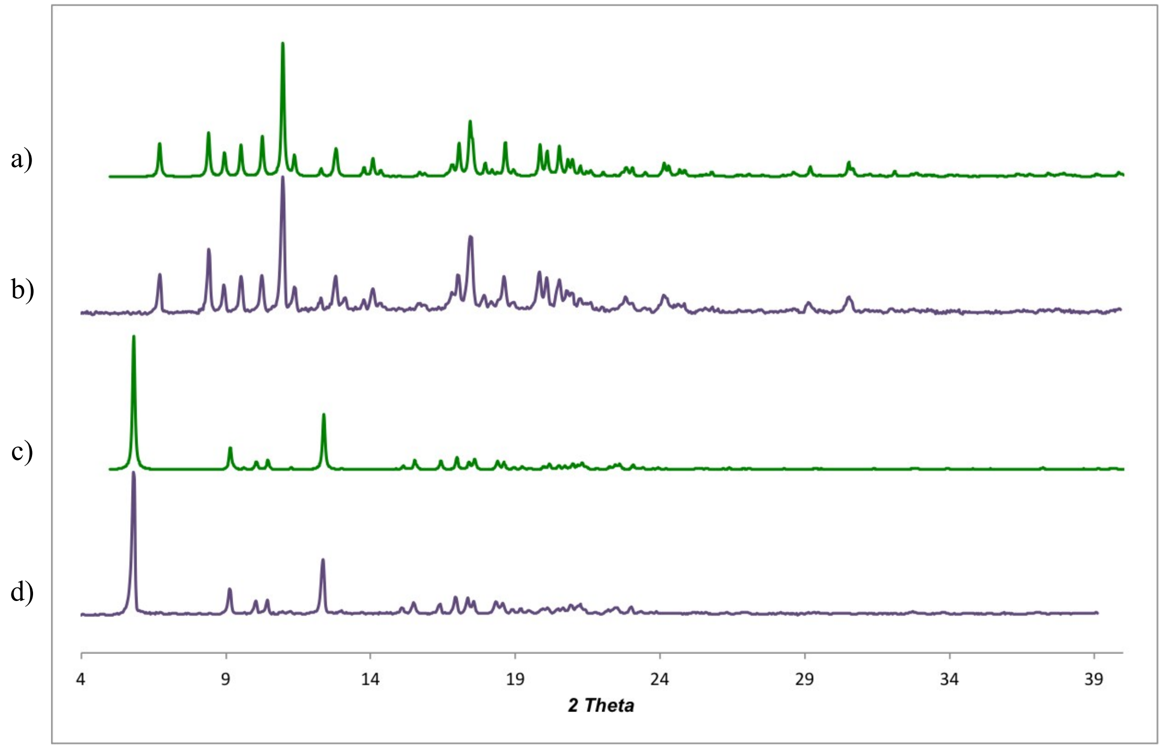

Figure S19. Powder X-ray diffraction data for the interconversion of complexes **5** and **6**: a) Simulated pattern of **6**; b) Crude milled product of **5** (1 equiv), **NMI** (2 equiv); c) Simulated pattern of **3**; d) Crude milled product of **6** (1 equiv), **2** (0.25 equiv)

*1.3.7.3 Conversion of pyridine and N-methyl imidazole complexes **3, 4, 5, 6** to 1,10-phenanthroline complex **7**;*

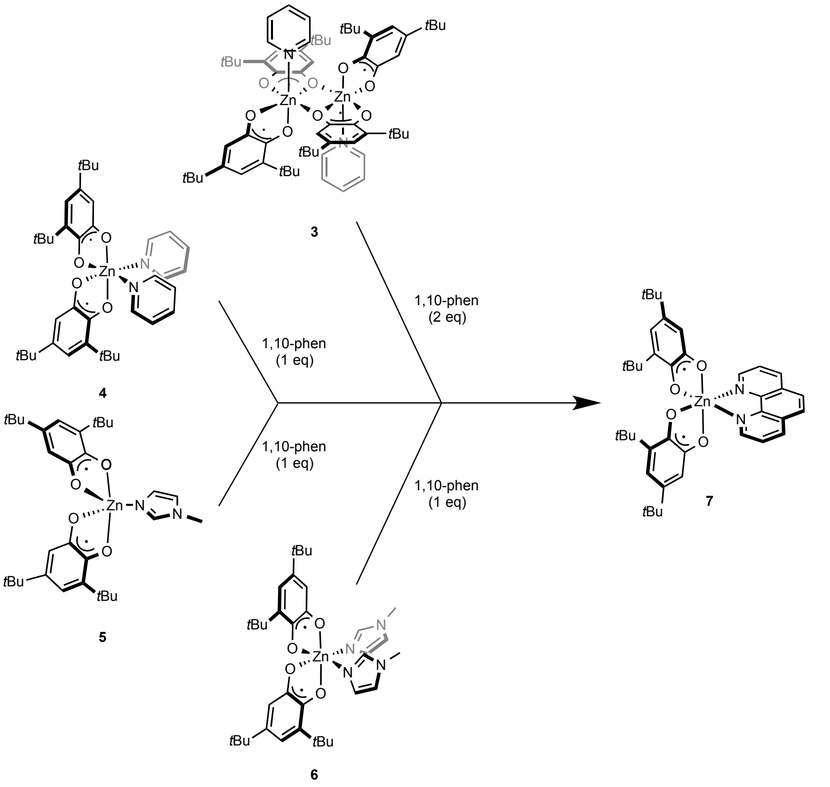

Scheme S27: (**3 to 7**)**: 3** (0.131 mmol, 152.9 mg, 1 equiv); **phen** (0.261 mmol, 47.1 mg, 1 equiv); (**4** to **7**)**: 4** (0.237 mmol, 157.3 mg, 1 equiv); **phen** (0.237 mmol, 42.7 mg, 1 equiv); (**5** to **7**)**: 5** (0.260 mmol, 153.1 mg, 1 equiv); **phen** (0.260 mmol, 46.9 mg, 1 equiv); (**6** to **7**)**: 6** (0.235 mmol, 157.6 mg, 1 equiv); **phen** (0.235 mmol, 42.4 mg, 1 equiv)

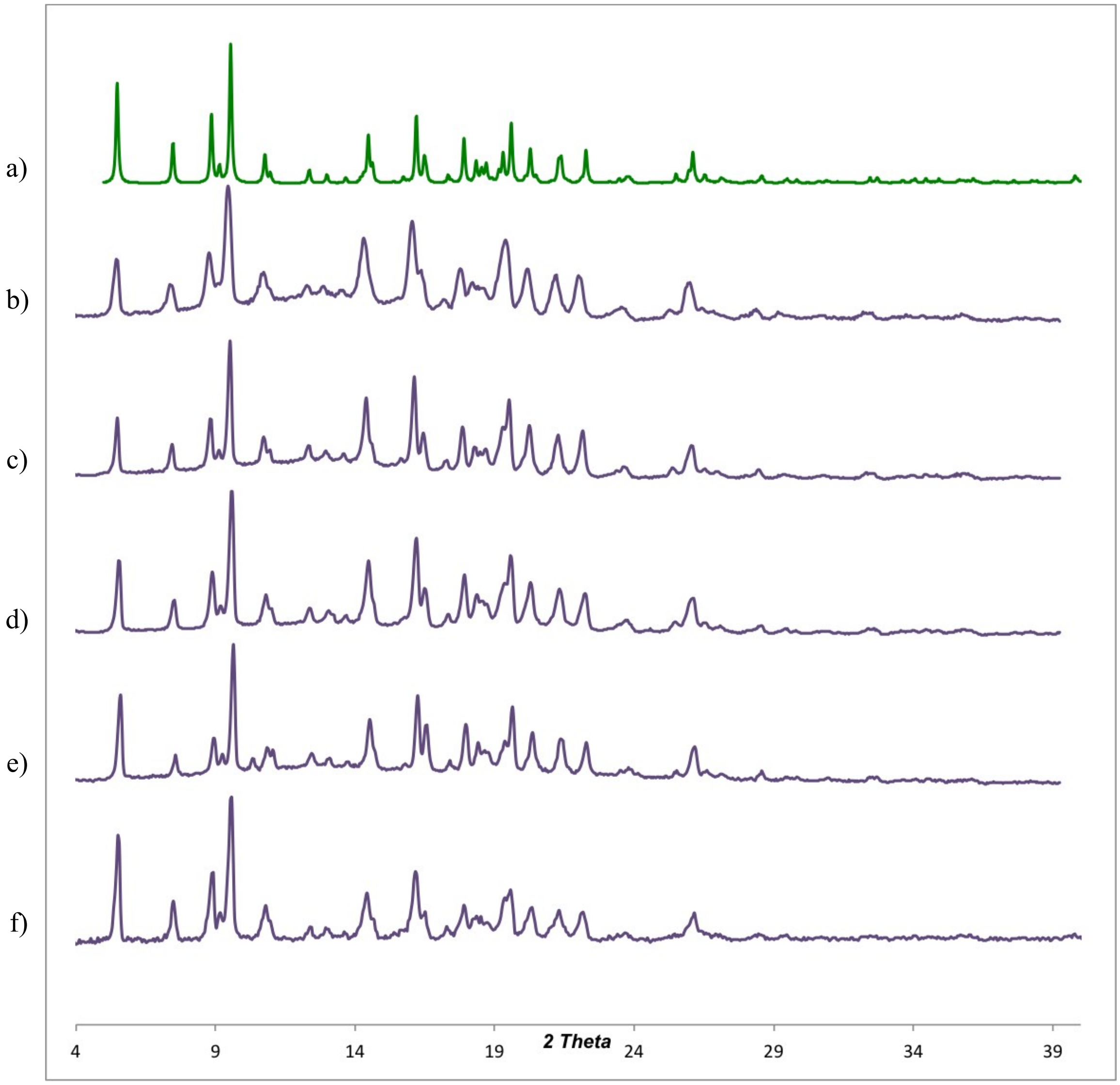

Figure S20. Powder X-ray diffraction data for the conversion of complexes **3, 4, 5, 6** to complex **7:** a) Simulated pattern of **7**; b) Crude milled product of **3** (1 equiv), **phen** (2 equiv); c) Crude milled product of **4** (1 equiv), **phen** (1 equiv); d) Crude milled product of **5** (1 equiv), **phen** (1 equiv); e) Crude milled product of **6** (1 equiv), **phen** (1 equiv); f) Crude milled product of **7** (1 equiv), **Py** (2 equiv).

1.3.8 Alternate Metal Reactivity

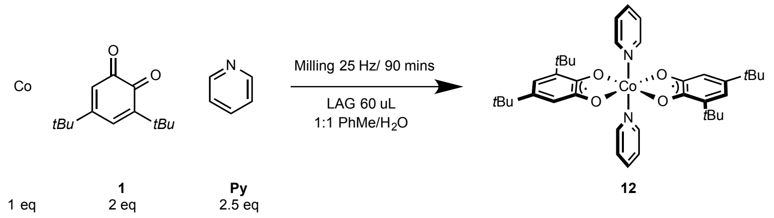

Scheme S28: Cobalt Powder (0.287 mmol, 16.9 mg, 1 equiv); **1** (0.574 mmol, 126 mg, 2 equiv); **Py** (0.717 mmol, 56.7 mg, 57.8 uL, 2.5 equiv)

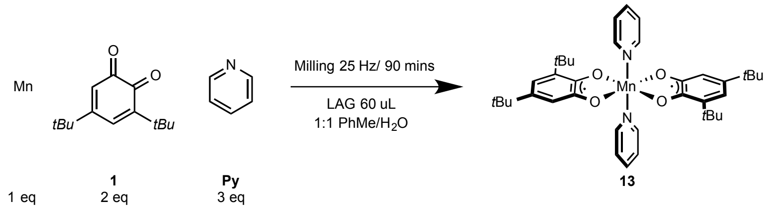

Scheme S29: Manganese Powder (0.273 mmol, 15.0 mg, 1 equiv); **1** (0.546 mmol, 120 mg, 2 equiv); **Py** (0.819 mmol, 64.8 mg, 65.9 uL, 3 equiv)

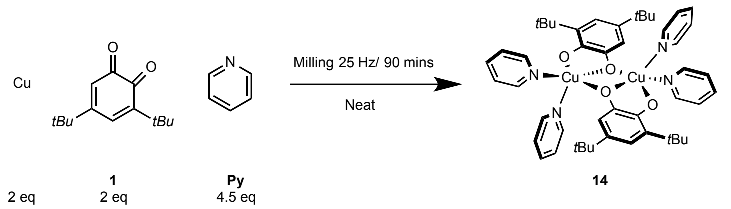

Scheme S30: Copper Powder (0.433 mmol, 27.5 mg, 2 equiv); **1** (0.433 mmol, 95.4 mg, 2 equiv); **Py** (0.974 mmol, 77.1 mg, 78.5 uL, 4.5 equiv) Reactants were milled NEAT.

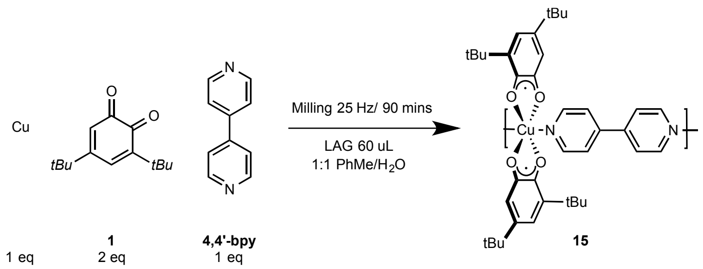

Scheme S31: Copper Powder (0.303 mmol, 19.2 mg, 1 equiv); **1** (0.606 mmol, 133.4 mg, 2 equiv); 4,4'-bipyridine (**4,4'-bpy**) (0.303 mmol, 47.3 mg, 1 equiv)

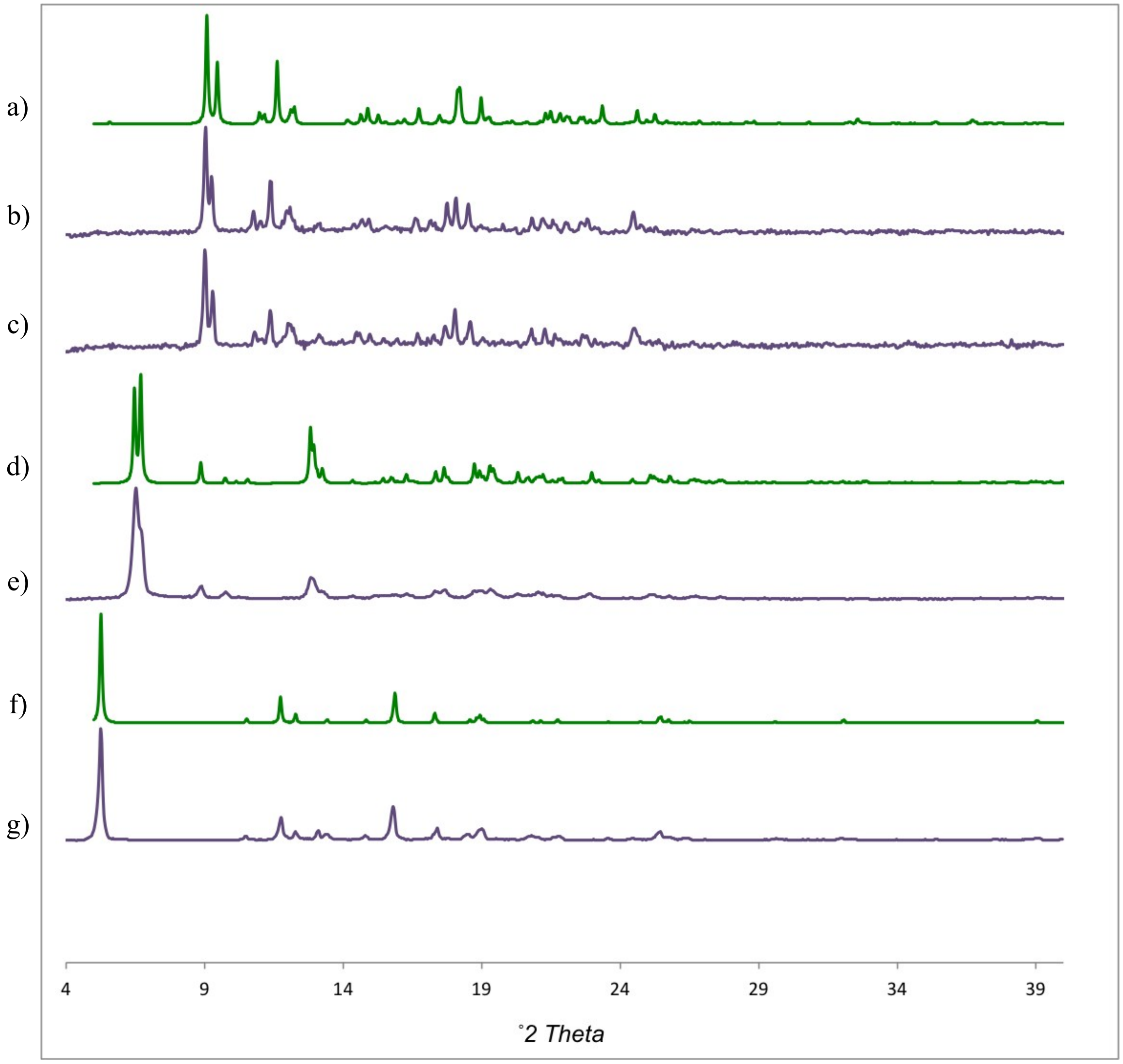

Figure S21. Powder X-ray diffraction data of mechanochemical reactivity of **1** with Cobalt, Manganese and Copper a) Simulated pattern of Co(3,5-dtbsq)$_2$(pyridine)$_2$-(pyridine)$_{0.5}$ complex PUTFAM from CCDC database; b) Crude milled product of Co (1 equiv), **1** (2 equiv), **Py** (2.5 equiv); c) Crude milled product of Mn (1 equiv), **1** (2 equiv), **Py** (3 equiv); d) Simulated pattern of Cu$_2$(3,5-dtbc)$_2$(pyridine)$_4$-(pyridine)$_{0.5}$ complex PITSIU from CCDC database; e) Crude milled product of Cu (2 equiv), **1** (2 equiv), **Py** (4.5 equiv), Reactants were milled NEAT; f) Simulated pattern of Cu(3,5-dtbsq)$_2$(4,4'-bipyridine) complex DUDZOR from CCDC database; g) Crude milled product of Cu (1 equiv), **1** (2 equiv), **4,4'-bpy** (1 equiv)

1.4 Solvothermal Synthesis with respective PXRD patterns

We have attempted to prepare complexes **2-7** and **9-11** by solvothermal synthesis using the following general procedure.

A flame-dried, round-bottom flask, equipped with a Teflon coated magnetic stir bar was charged with Zn (2 mmol, 130.7 mg, 1 equiv), **2** *(4 mmol, 881 mg, 2 equiv) and amine ligand* ***XX****. To this solid mixture, 30 mL of inert anhydrous solvent was added via syringe. The reaction mixturewas then bubbled with* N_2 *for 5 minutes. Three different reaction conditions were attempted. Condition* ***A****: 24h reflux in anhydrous toluene; Condition* ***B****: 90 mins reflux in anhydrous toluene; Condition* ***C****: 90 mins reflux in a 1 to 1 mixture of toluene and* H_2O*. Resulting reactions mixtures were collected and the solvent removed under vacuo, the resulting crude solids were then analyzed by PXRD.*

*1.4.1 Solvothermal Synthesis of complex **2** from Zinc Powder*

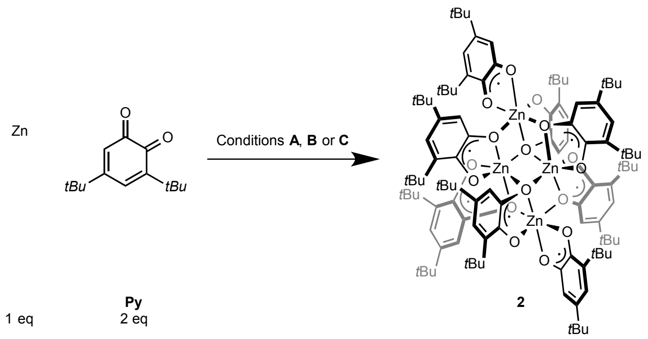

Scheme S32: Zn (2 mmol, 130.7 mg, 1 equiv), **1** (4 mmol, 881 mg, 2 equiv)

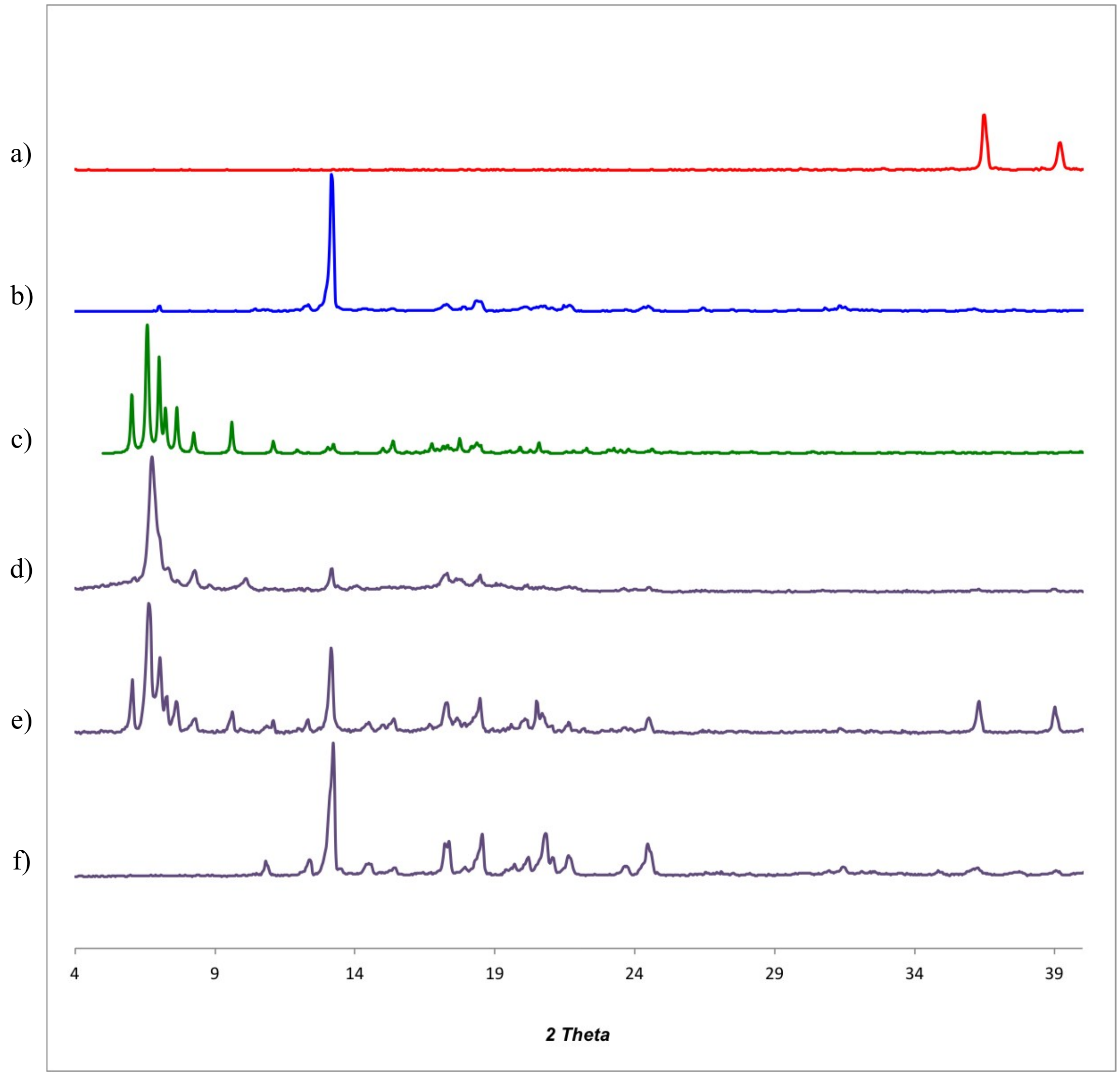

Figure S22. Powder X-ray diffraction data for the solvothermal synthesis of **2** from Zn: a) Zn; b) **1**; c) Simulated pattern of $[Co^{II}(3,5\text{-dtbsq})_2]_4$(benzene) tetranuclear complex TBSQCO from CCDC database; d) Crude product of Zn (1 equiv), **1** (2 equiv) Condition **A**; e) Crude product of Zn (1 equiv), **1** (2 equiv) Condition **B**; f) Crude product of Zn (1 equiv), **1** (2 equiv) Condition **C**

1.4.2 Solvothermal Synthesis of complex **2** *from Zinc Oxide*

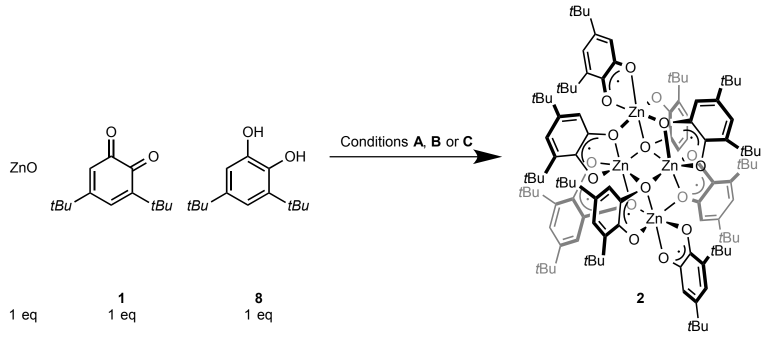

Scheme S33: ZnO (2 mmol, 162.8 mg, 1 equiv), **1** (2 mmol, 440.62 mg, 1 equiv), **8** (2 mmol, 444.6 mg, 1 equiv).

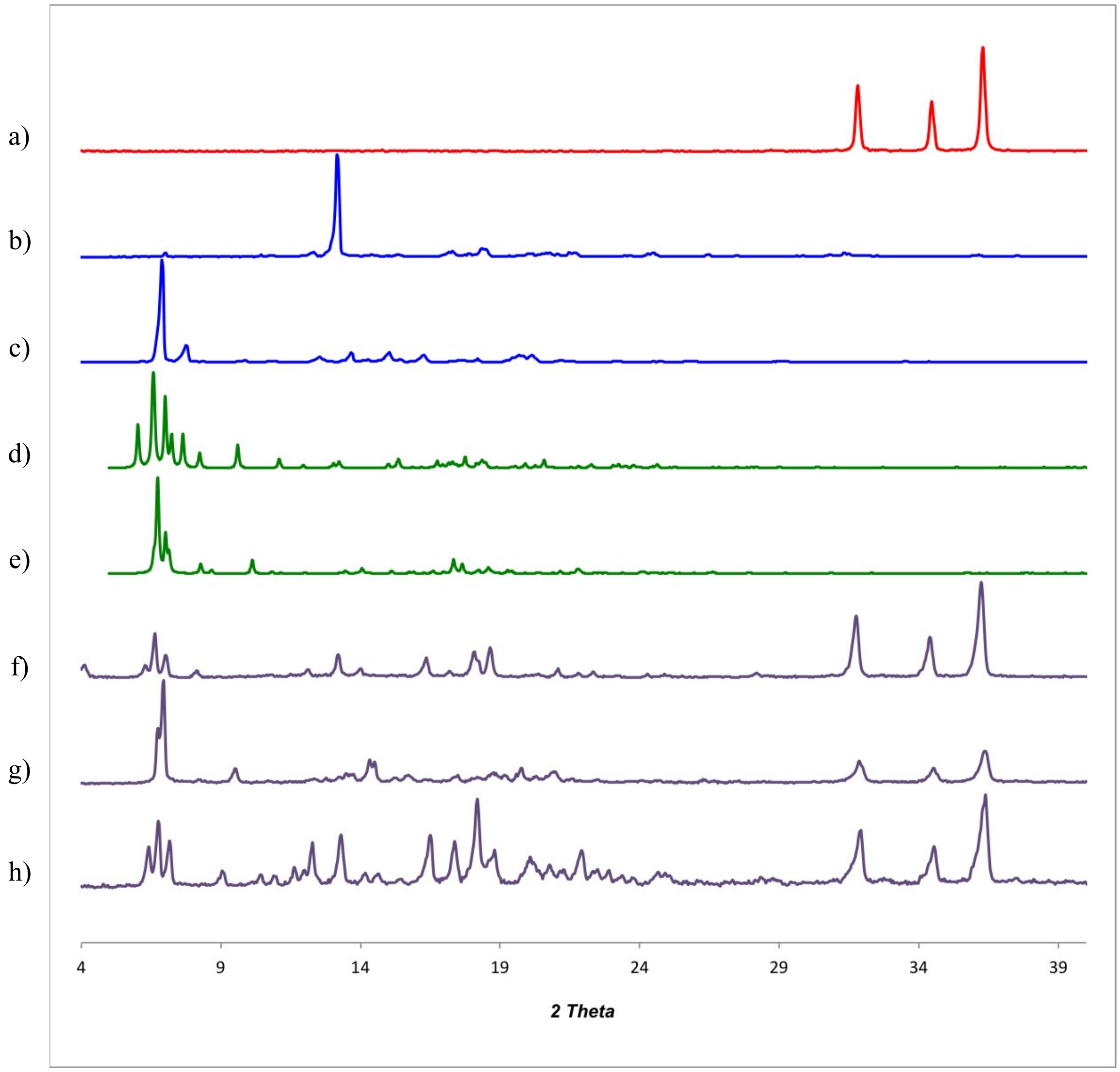

Figure S23. Powder X-ray diffraction data for the solvothermal synthesis of **2** from ZnO: a) ZnO; b) **1**; c) **8**; d) Simulated pattern of $[Co^{II}(3,5\text{-dtbsq})_2]_4$(benzene) tetranuclear complex TBSQCO from CCDC database; e) Simulated pattern of $[Mn^{II}(3,5\text{-dtbsq})_2]_4$ tetranuclear complex BANVOB from CCDC database; f) Crude product of ZnO (1 equiv), **1** (1 equiv), **8** (1 equiv) Condition **A**; g) Crude product of ZnO (1 equiv), **1** (1 equiv), **8** (1 equiv) Condition **B**; f) Crude product of ZnO (1 equiv), **1** (1 equiv), **8** (1 equiv) Condition **C**;

1.4.3 Solvothermal Synthesis of complexes **3** *and* **4**

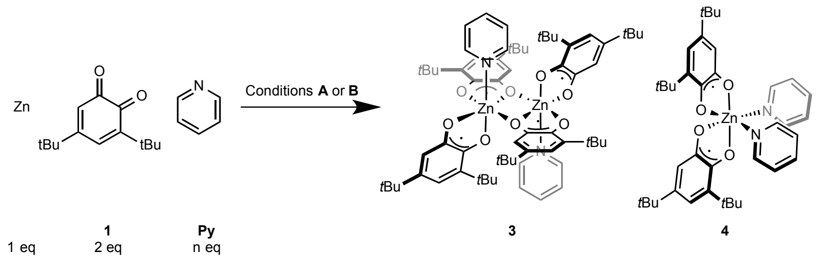

Scheme S34: (**n=1**) Zn (1.705 mmol, 111.5 mg, 1 equiv); **1** (3.41 mmol, 753 mg, 2 equiv); **Py** (1.705 mmol, 133.0 mg, 135.5 uL, 1 equiv); (**n=2**) Zn (1.505 mmol, 98.5 mg, 1 equiv); **1** (3.01 mmol, 665 mg, 2 equiv); **Py** (3.01 mmol, 238.1 mg, 242.5 uL, 2 equiv); (**n=10**) Zn (1.505 mmol, 98.5 mg, 1 equiv); **1** (3.01 mmol, 665 mg, 2 equiv); **Py** (15.05 mmol, 1190.5 mg, 1212.5 uL, 10 equiv)

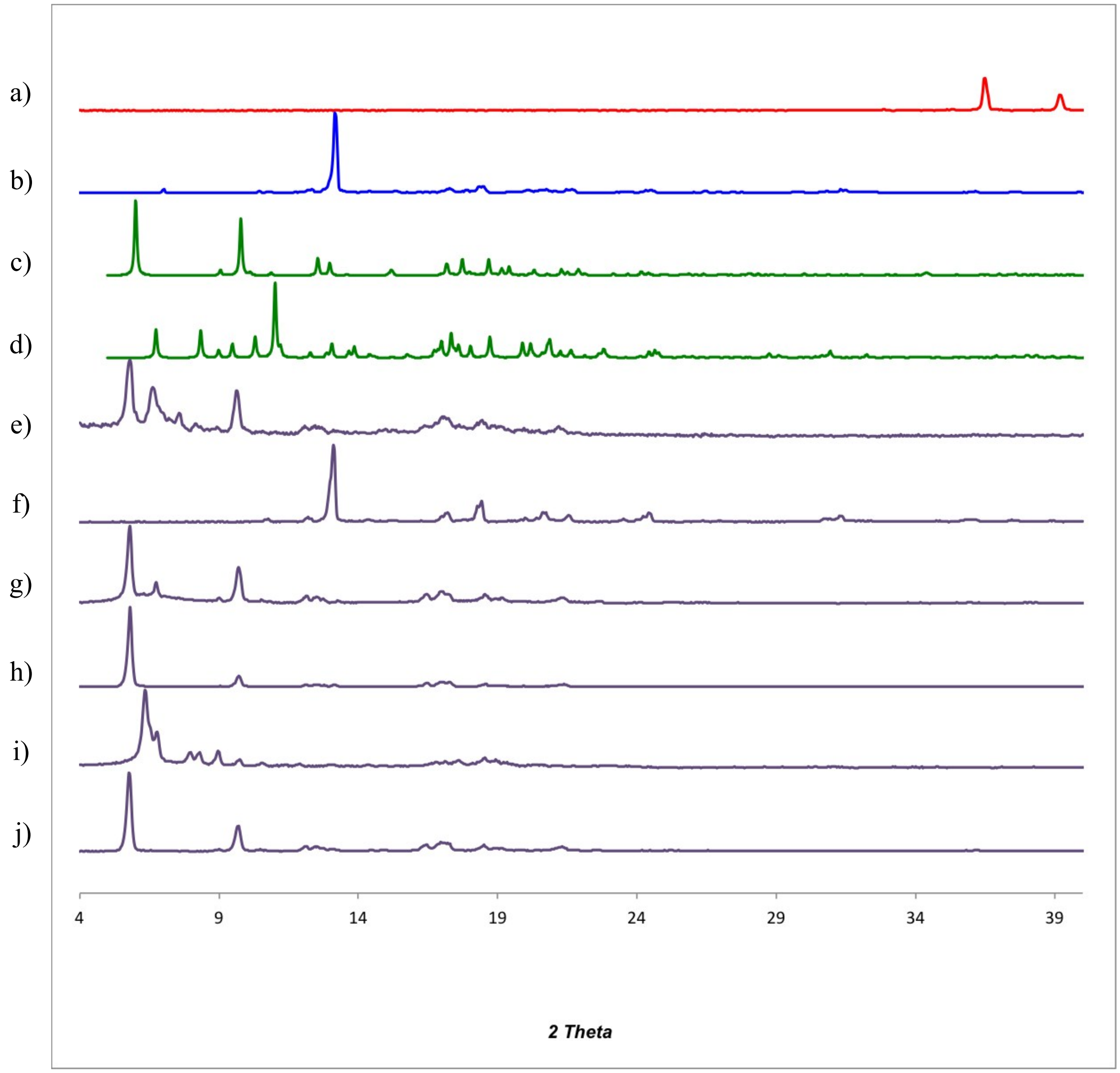

Figure S24. Powder X-ray diffraction data for the synthesis of **3** and **4** from Zn: a) Zn; b) **1**; c) Simulated pattern of **3**; d) Simulated pattern of **4**; e) Crude product of Zn (1 equiv), **1** (2 equiv), **Py** (1 equiv) Condition **A**; f) Crude product of Zn (1 equiv), **1** (2 equiv), **Py** (1 equiv) Condition **B**; g) Crude product of Zn (1 equiv), **1** (2 equiv), **Py** (2 equiv) Condition **A**; h) Crude product of Zn (1 equiv), **1** (2 equiv), **Py** (2 equiv) Condition **B**; i) Crude product of Zn (1 equiv), **1** (2 equiv), **Py** (10 equiv) Condition **A**; j) Crude product of Zn (1 equiv), **1** (2 equiv), **Py** (10 equiv) Condition **B**;

1.4.4 *Solvothermal Synthesis of complexes* **5** *and* **6**

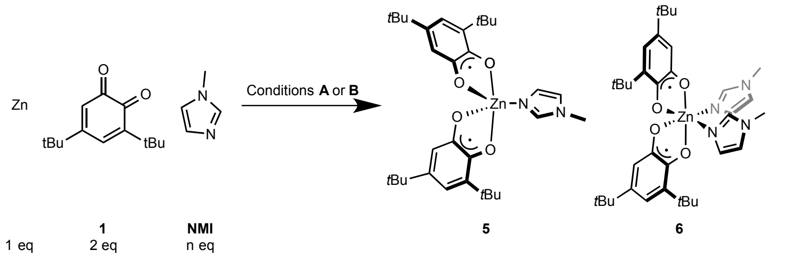

Scheme S35: (**n=1**) Zn (1.70 mmol, 111.5 mg, 1 equiv); **1** (3.40 mmol, 749 mg, 2 equiv); **NMI** (1.705 mmol, 133.0 mg, 135.5 uL, 1 equiv) (**n=2**) Zn (1.49 mmol, 97.5 mg, 1 equiv); **1** (2.98 mmol, 657.5 mg, 2 equiv); **NMI** (2.98 mmol, 245.1 mg, 238 uL, 2 equiv); (**n=10**) Zinc Powder (1.70 mmol, 111 mg, 1 equiv); **1** (3.40 mmol, 749 mg, 2 equiv); **NMI** (17.0 mmol, 1190.5 mg, 1190 uL, 10 equiv)

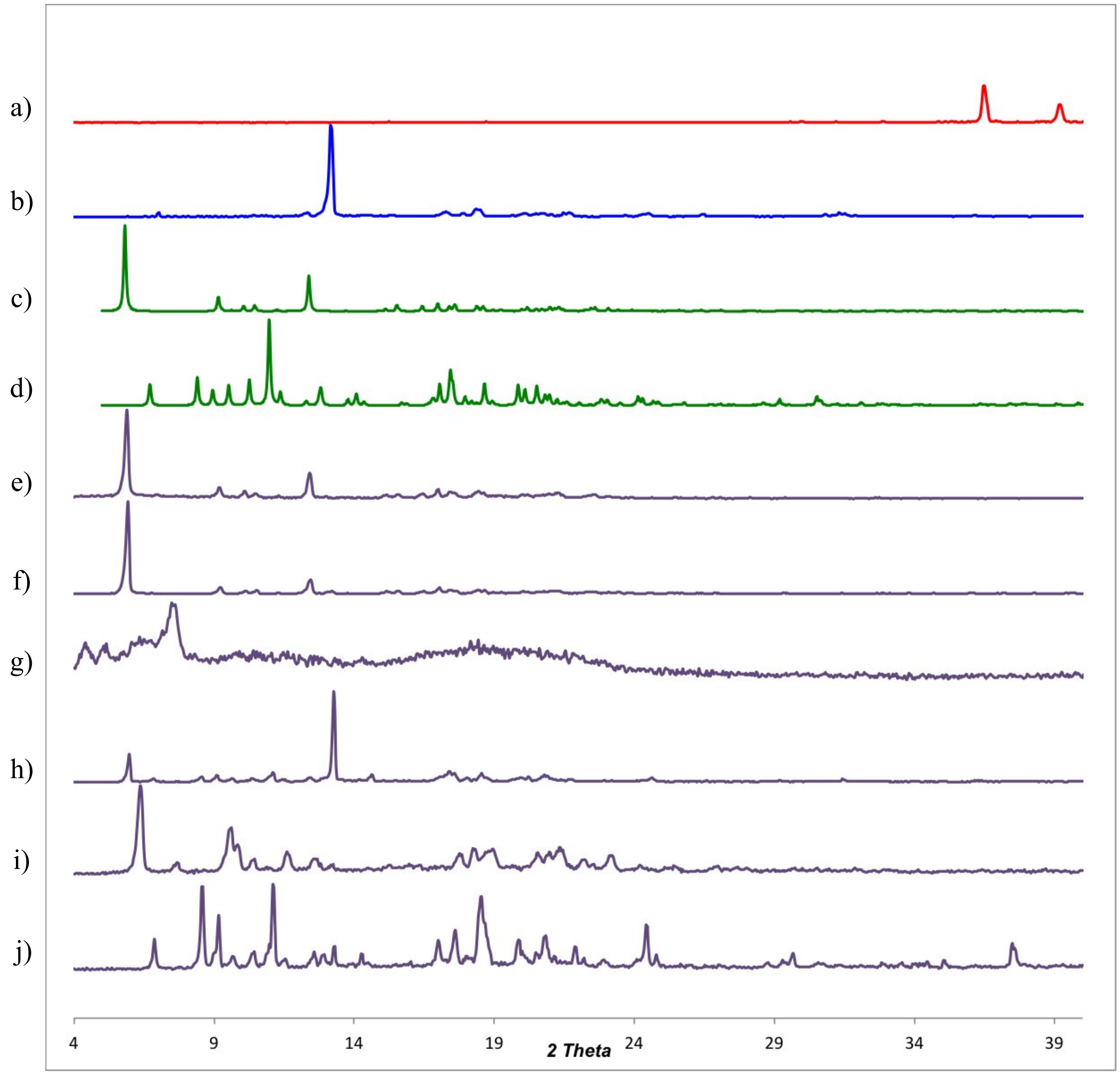

Figure S25. Powder X-ray diffraction data for the synthesis of **5** and **6** from Zn: a) Zn; b) **1**; c) Simulated pattern of **5**; d) Simulated pattern of **6**; e) Crude product of Zn (1 equiv), **1** (2 equiv), **NMI** (1 equiv) Condition **A**; f) Crude product of Zn (1 equiv), **1** (2 equiv), **NMI** (1 equiv) Condition **B**; g) Crude product of Zn (1 equiv), **1** (2 equiv), **NMI** (2 equiv) Condition **A**; h) Crude product of Zn (1 equiv), **1** (2 equiv), **NMI** (2 equiv) Condition **B**; i) Crude product of Zn (1 equiv), **1** (2 equiv), **NMI** (10 equiv) Condition **A**; j) Crude product of Zn (1 equiv), **1** (2 equiv), **NMI** (10 equiv) Condition **B**;

1.4.5 Solvothermal Synthesis of complex **7**

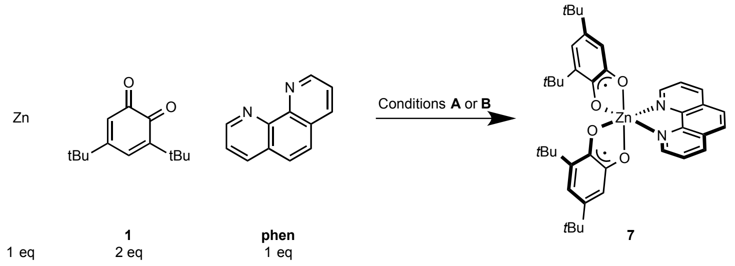

Scheme S36: Zn (1.42 mmol, 92.8 mg, 1 equiv); **1** (2.84 mmol, 626 mg, 2 equiv); **phen** (1.42 mmol, 281.5 mg, 1 equiv)

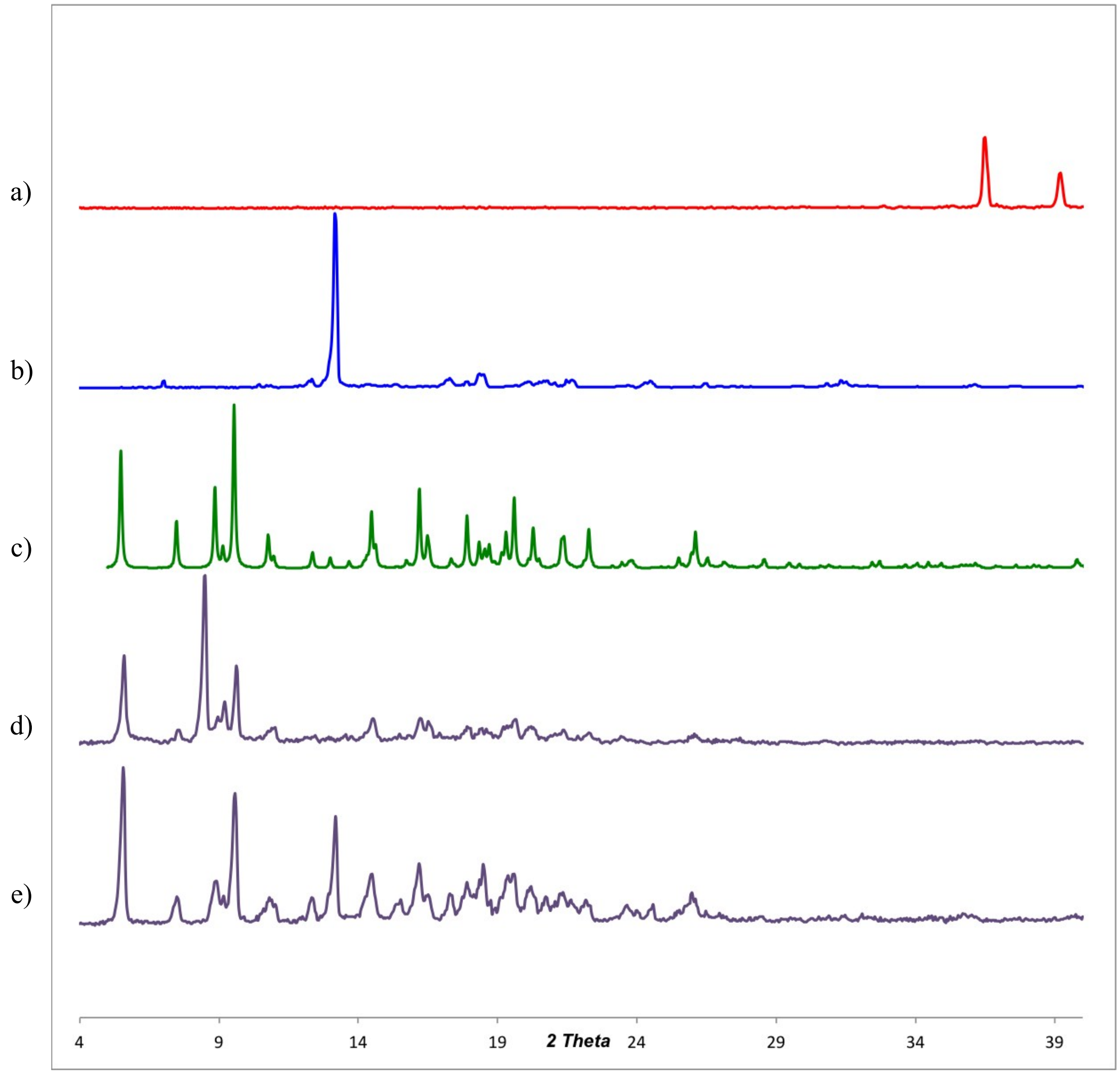

Figure S26. Powder X-ray diffraction data for the solvothermal synthesis of **7** from Zn: a) Zn; b) **1**; c) Simulated pattern of **7**; d) Crude product of Zn (1 equiv), **1** (2 equiv), **phen** (1 equiv) Condition **A**; e) Crude product of Zn (1 equiv), **1** (2 equiv), **phen** (1 equiv) Condition **B**;

1.4.6 Solvothermal Synthesis of complexes **9** *and* **10**

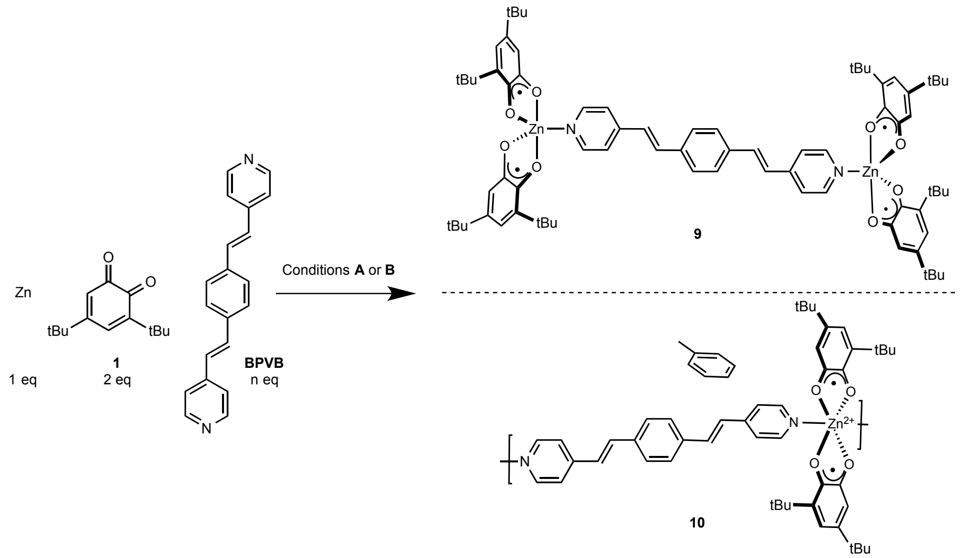

Scheme S37: (**n=0.5**) Zn (1.54 mmol, 101 mg, 1 equiv); **1** (3.09 mmol, 680 mg, 2 equiv); **BPVB** (0.772 mmol, 219.5 mg, 0.5 equiv) (**n=1**) Zn (1.27 mmol, 82.5 mg, 1 equiv); **1** (2.33 mmol, 557.5 mg, 2 equiv); **BPVB** (1.27 mmol, 360 mg, 1 equiv)

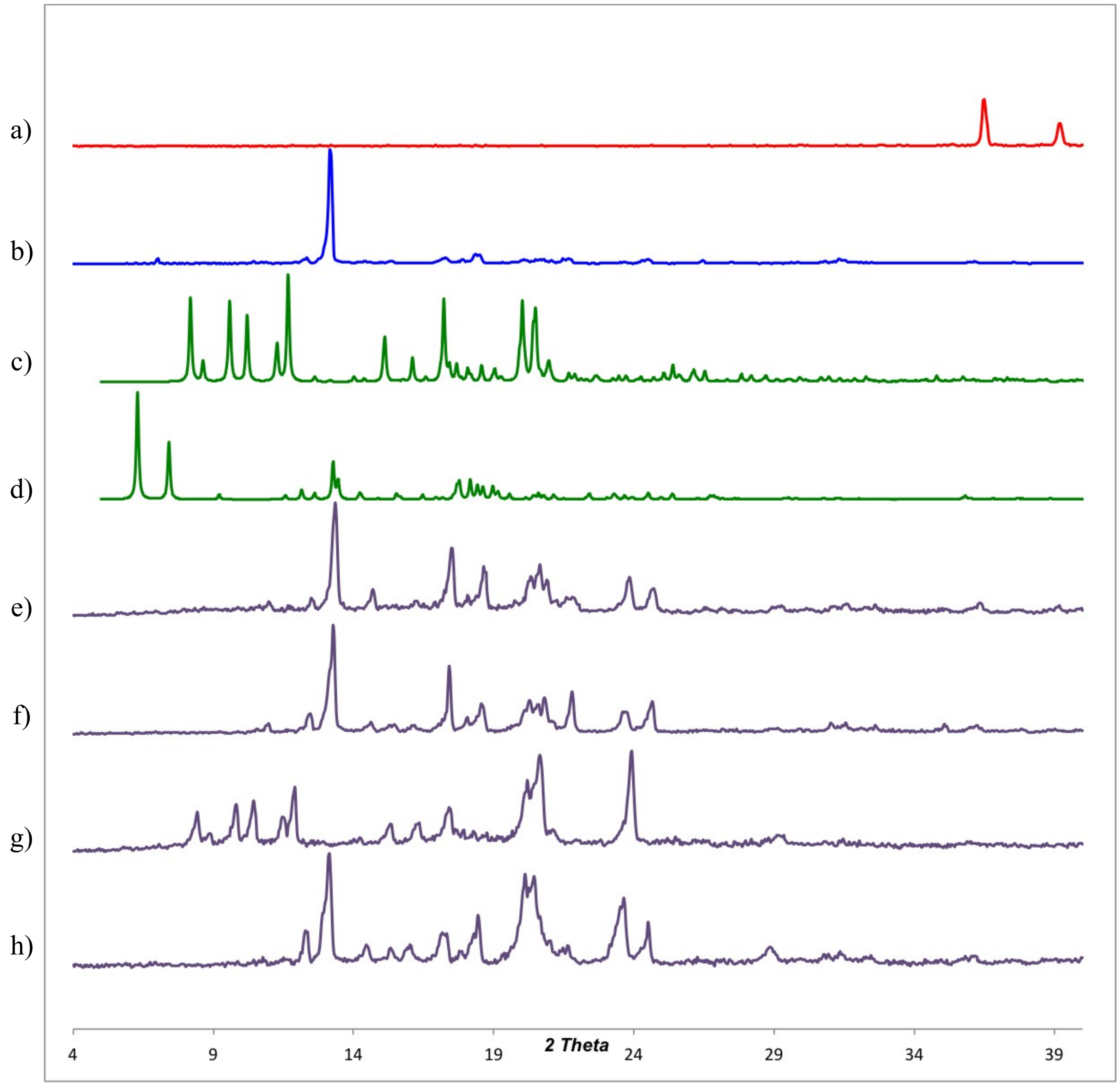

Figure S27. Powder X-ray diffraction data for the synthesis of **9** and **10** from Zn: a) Zn; b) **1**; c) Simulated pattern of **9**; d) Simulated pattern of **10**; e) Crude product of Zn (1 equiv), **1** (2 equiv), **BPVB** (0.5 equiv) Condition **A**; f) Crude product of Zn (1 equiv), **1** (2 equiv), **BPVB** (0.5 equiv) Condition **B**; g) Crude product of Zn (1 equiv), **1** (2 equiv), **BPVB** (1 equiv) Condition **A**; h) Crude product of Zn (1 equiv), **1** (2 equiv), **BPVB** (1 equiv) Condition **B**;

1.4.7 Solvothermal Synthesis of complex **11**

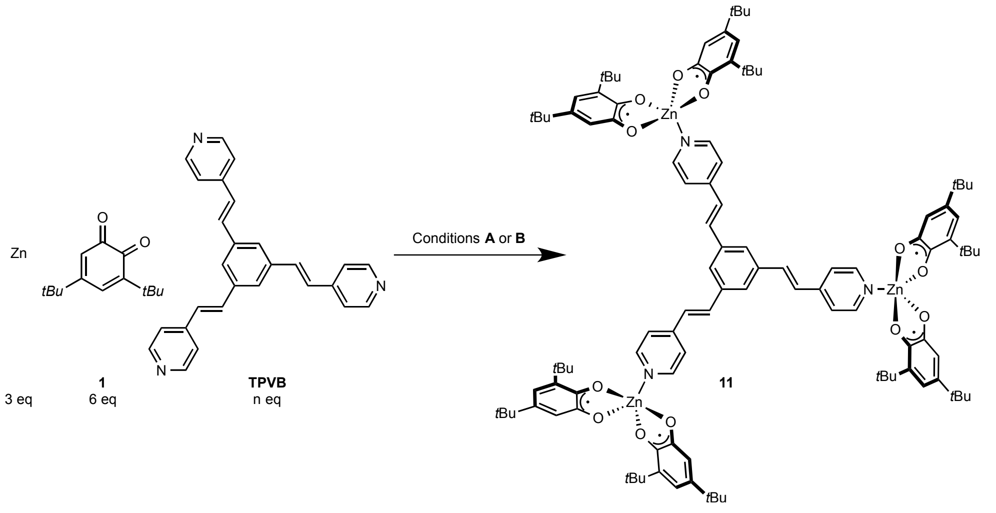

Scheme S38: (**n=1**) Zn (1.57 mmol, 102.9 mg, 3 equiv); **1** (3.15 mmol, 693 mg, 6 equiv); **TPVB** (0.525 mmol, 203 mg, 1 equiv); (**n=2**) Zn (1.31 mmol, 85.5 mg, 3 equiv); **1** (2.62 mmol, 576 mg, 6 equiv); **TPVB** (0.872 mmol, 338 mg, 2 equiv)

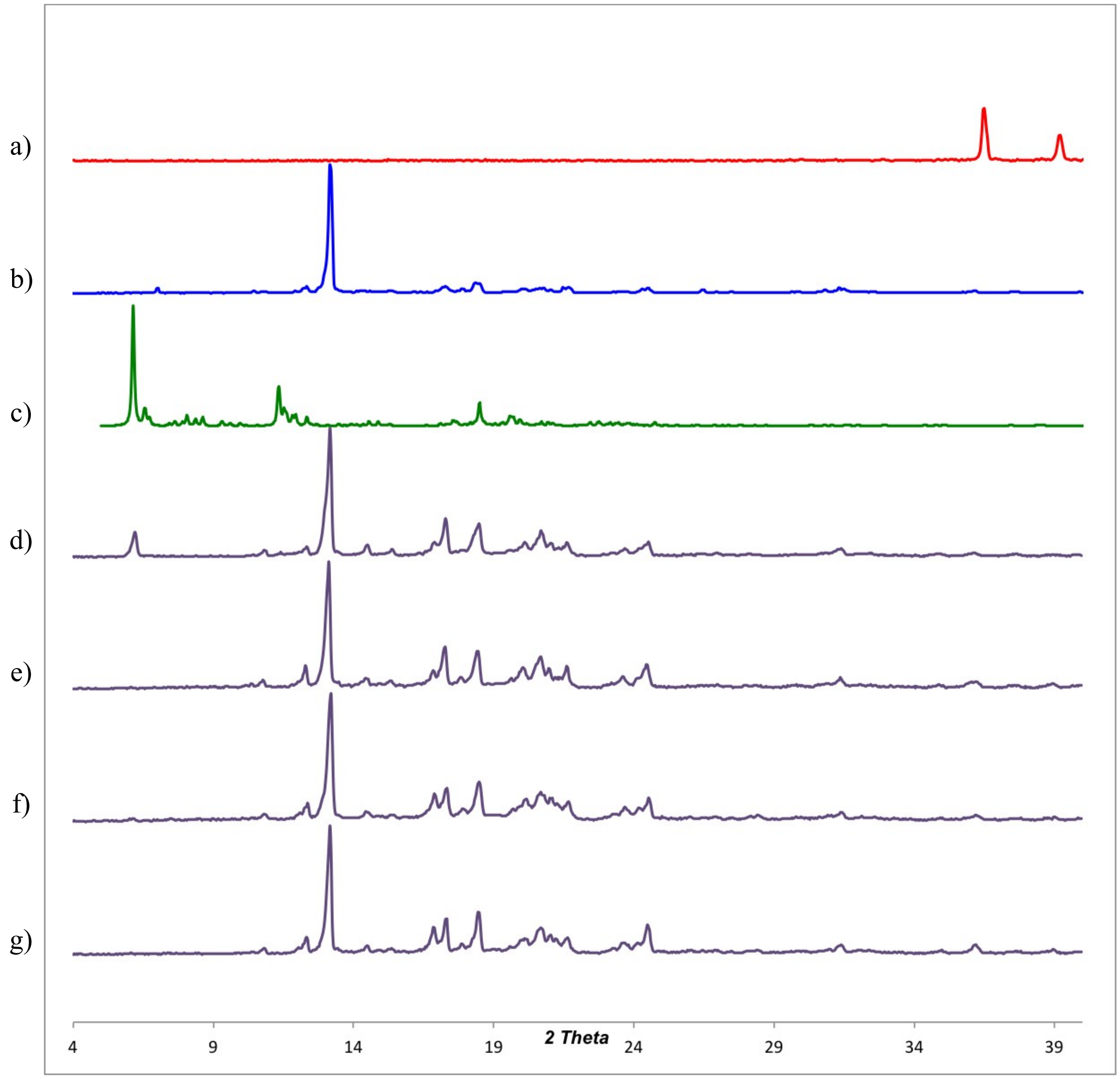

Figure S28. Powder X-ray diffraction patterns for the synthesis of **11** from Zn: a) Zn; b) **1**; c) Simulated pattern of **11**; d) Crude product of Zn (3 equiv), **1** (6 equiv), **TPVB** (1 equiv) Condition **A**; e) Crude product of Zn (3 equiv), **1** (6 equiv), **TPVB** (1 equiv) Condition **B**; f) Crude product of Zn (3 equiv), **1** (6 equiv), **TPVB** (2 equiv) Condition **A**; g) Crude product of Zn (3 equiv), **1** (6 equiv), **TPVB** (2 equiv) Condition **B**;

1.5 Conditions for Single crystal growth by recrystallization

1.5.1 $Zn_2(3,5\text{-}dtbsq)_4(pyridine)_2$ (**3**)

Crude milled product was dissolved in a minimal amount of toluene and filtered through celite to remove any insoluble impurities. 1 mL of the resulting filtrate was put into a vial and was recrystallized by vapour diffusion using petroleum ether as an anti-solvent resulting in small rectangular blue plates.

1.5.2 $Zn(3,5\text{-}dtbsq)_2(pyridine)_2$ (**4**)

Crude milled product was dissolved in toluene and filtered through celite to remove any insoluble impurities. The resulting filtrate was slowly evaporated in a recrystallization dish resulting in small blue plates.

1.5.3 $Zn(3,5\text{-}dtbsq)_2$(N-Methyl Imidazole) (**5**)

Crude milled product was dissolved in a minimal amount of dichloromethane and filtered through celite to remove any insoluble impurities. 1 mL of the resulting filtrate was put into a vial and was recrystallized by vapour diffusion using pentane as an anti-solvent resulting in big dark rectangular blue plates.

1.5.4 $Zn(3,5\text{-}dtbsq)_2(N\text{-}Methyl\ Imidazole)_2$ (**6**)

Crude milled product was dissolved in toluene and filtered through celite to remove any insoluble impurities. The resulting filtrate was slowly evaporated in a recrystallization dish resulting in big dark rectangular blue plates.

1.5.5 $Zn(3,5\text{-}dtbsq)_2$(1,10-phenanthroline) (**7**)

Crude milled product was dissolved in toluene and filtered through celite to remove any insoluble impurities. The resulting filtrate was slowly evaporated in a recrystallization dish resulting in small green plates.

1.5.6 $Zn_2(3,5\text{-}dtbsq)_4$(1,4-bis(2-(pyridin-4-yl)vinyl)benzene) (**9**)

Crude milled product was dissolved in a minimal amount of chloroform and filtered through celite to remove any insoluble impurities. 1 mL of the resulting filtrate was put into a vial and was recrystallized slowly by vapour diffusion using petroleum ether as an anti-solvent and kept at -20°C resulting in flat rectangular green plates after 2-3 days.

1.5.7 $Zn(3,5\text{-}dtbsq)_2$(1,4-bis(2-(pyridin-4-yl)vinyl)benzene) (**10**)

0.2 mmol of 1,4-bis(2-(pyridin-4-yl)vinyl)benzene was dissolved in 10 mL toluene in vial A and 0.1 mmol of $[Zn(3,5\text{-}dtbsq)_2]_4$ was dissolved in 10 mL toluene in vial B. The contents of vial B were syringed in the bottom of vial A using a long metal needle creating two distinct layers. Special care was made to ensure two distinct layers and the mixture was left

undisturbed. Translucent intense green/orange parallelogram plates were collected after 48 hours.

1.5.8 Zn_3(3,5-dtbsq)$_6$(1,3,5-tris(2-(pyridin-4-yl)vinyl)benzene) (**11**)

0.4 mmol of 1,3,5-tris(2-(pyridin-4-yl)vinyl)benzene was dissolved in 10 mL toluene in vial A and 0.3 mmol of [Zn(3,5-dtbsq)$_2$]$_4$ was dissolved in 10 mL toluene in vial B. The contents of vial B were syringed in the bottom of vial A using a long metal needle creating two distinct layers. Special care was made to ensure two distinct layers and the mixture was left undisturbed. Green plates were collected after 48 hours.

1.6 Solvent Evacuation of Zn_3(3,5-dtbsq)$_6$(TPVB)

Single crystals grown using conditions found in section *1.5.8* were analyzed by PXRD and TGA. The crystals were then subject to evacuation conditions (50˚C under high vacuum (4.5" Hg) for 12h) and re-analyzed by PXRD and TGA. The resulting data can be observed.

1.6.1 PXRD patterns

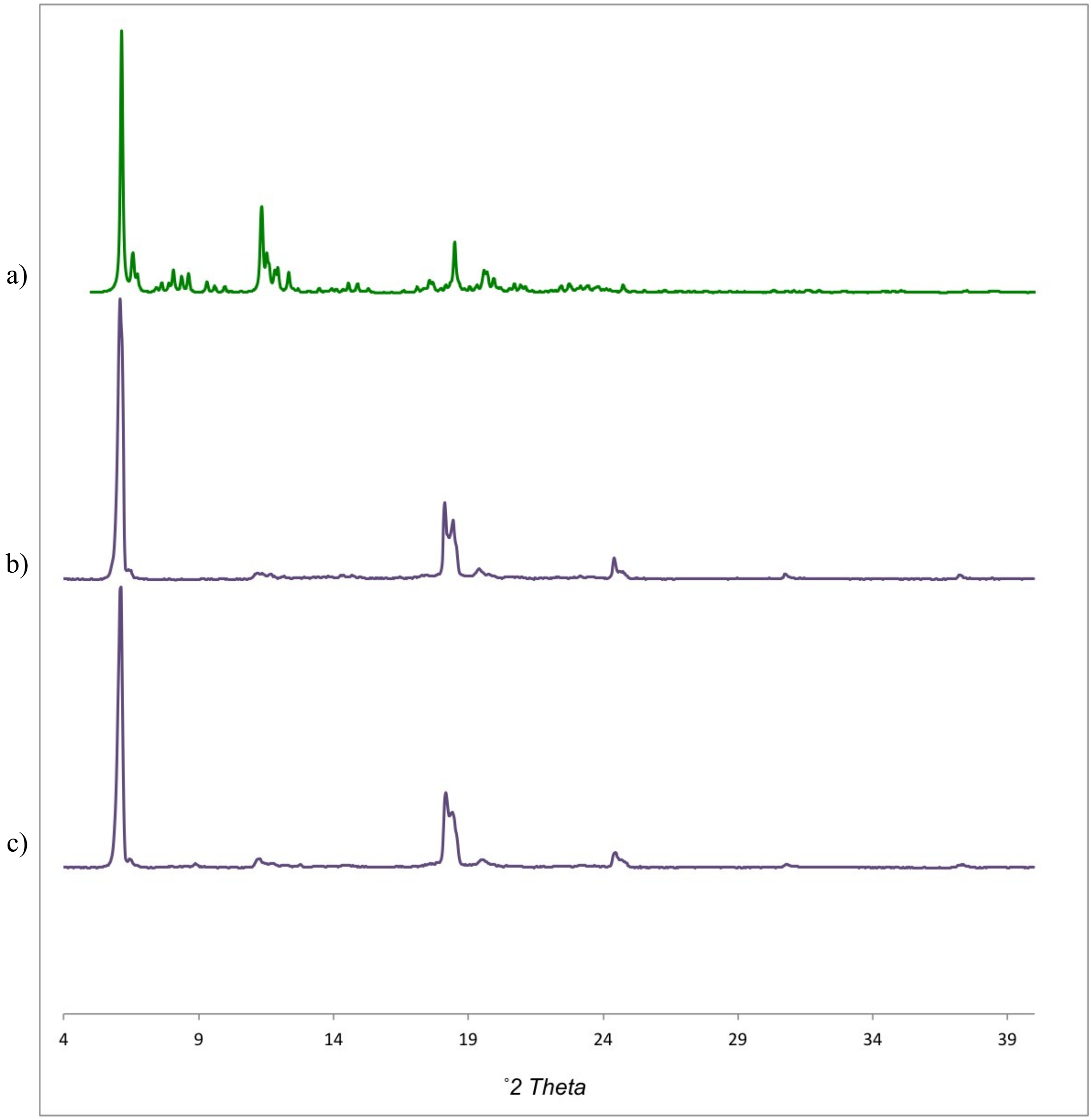

Figure S29: Powder X-ray diffraction data for the solvent evacuation of **11**: a) simulated pattern for **11;** b) single crystals of **11** grown from toluene; c) single crystals from b) evacuated at 50˚C for 12 h under high vac.

1.6.2 TGA thermograms

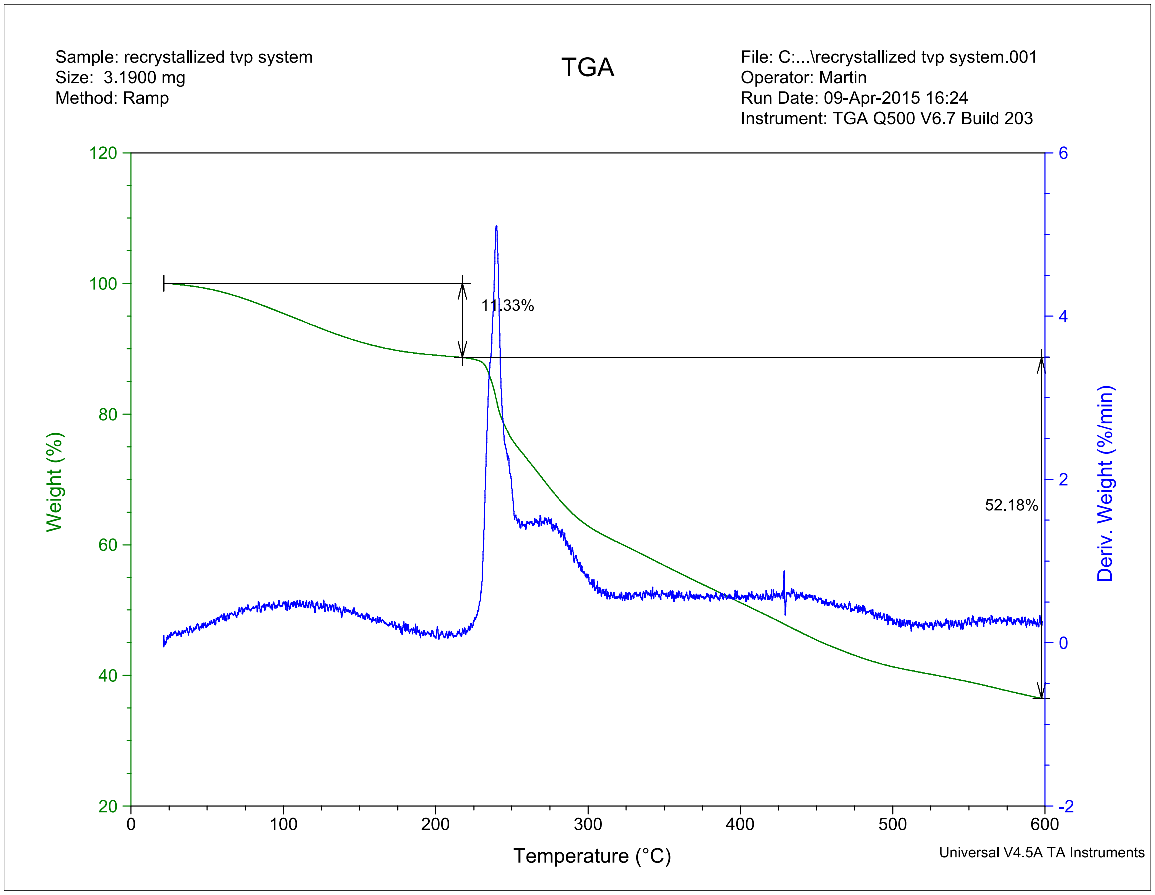

Figure S30: *TGA* of single crystals of **11** grown from toluene, TGA run was done under N_2.

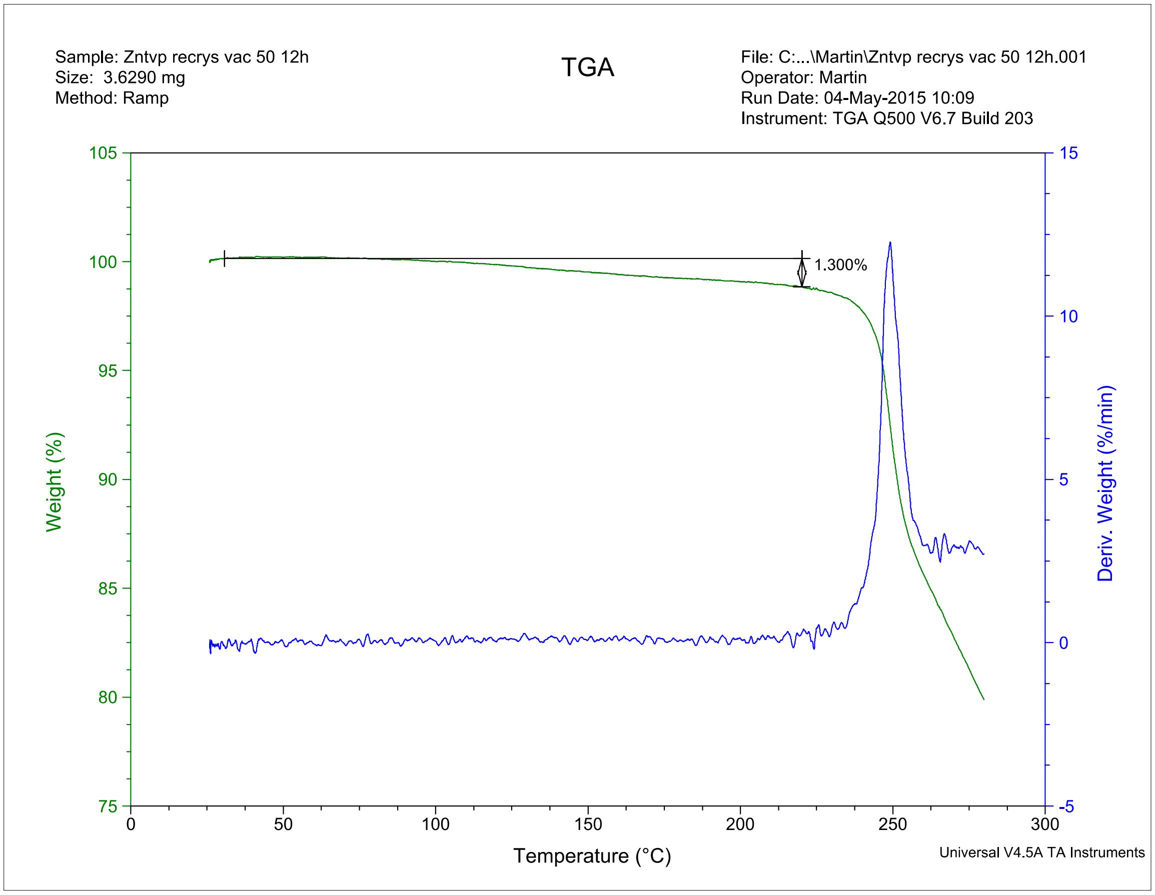

Figure S31: *TGA* of single crystals of **11** grown from toluene evacuated at 50°C for 12 h under high vac. TGA run was done under N_2

1.7 Ligand Synthesis

The following ligands were synthesized using a modified procedure from the work of Ward *Inorg. Chem.* **1995**, 34, 4828-4835.

1.7.1 1,4-bis(2-(pyridin-4-yl)vinyl)benzene (**BPVB**)

A flame-dried 10 mL vial equipped with a Teflon-coated stir bar and a rubber septum was charged with 1,4-dibromobenzene (8.48 mmol, 1.0 eq); $Pd(OAc)_2$ (0.471 mmol, 0.055 eq) and PPh_3 (0.508 mmol, 0.06 eq). Previously distilled Et_3N (8.5 mL) and 4-vinylpyridine (21.2 mmol, 2.5 eq) were syringed in. The solution was then flushed with N_2 for 5 mins and then capped and sealed tightly. The reaction was heated at 100˚C for 72h stirring vigorously. The resulting black residue was dissolved in 500 mL DCM and washed 3x with 200 mL H_2O. The organic layer was collected, dried with $MgSO_4$ and then rotovapped to afford a beige solid. The resulting beige solid was then recrystallized in acetone to afford the product.

1.7.2 1,3,5-tris(2-(pyridin-4-yl)vinyl)benzene (**TPVB**)

A flame-dried 10 mL vial equipped with a Teflon-coated stir bar and a rubber septum was charged with 1,3,5-tribromobenzene (2.02 mmol, 1.0 eq); $Pd(OAc)_2$ (0.53 mmol, 0.055 eq) and PPh_3 (0.38 mmol, 0.06 eq). Previously distilled Et_3N (6.4 mL) and 4-vinylpyridine (7.56 mmol, 3.75 eq) were syringed in. The solution was then flushed with N_2 for 5 mins and then capped and sealed tightly. The reaction was heated at 100˚C for 72h stirring vigorously. The resulting black residue was dissolved in 500 mL DCM and washed 3x with 200 mL H_2O. The organic layer was collected, dried with $MgSO_4$ and then rotovapped to afford a beige solid. The resulting beige solid was then recrystallized in acetone to afford the product.

2. Rendered Crystal Structures of complexes 3-7; 9-11

The following rendered images correspond to the crystal structures of complexes **3-7** and **9-11.** Note that hydrogen atoms and *tert*-butyl methyl groups have been omitted for clarity. Ball and stick models were rendered using POV-Ray.

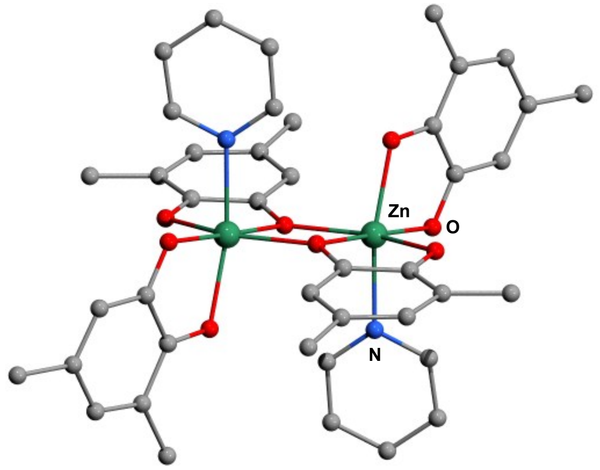

Figure S32: Ball-and-stick representation of a single molecule of Zn_2(3,5-dtbsq)$_4$(pyridine)$_2$ (**3**).

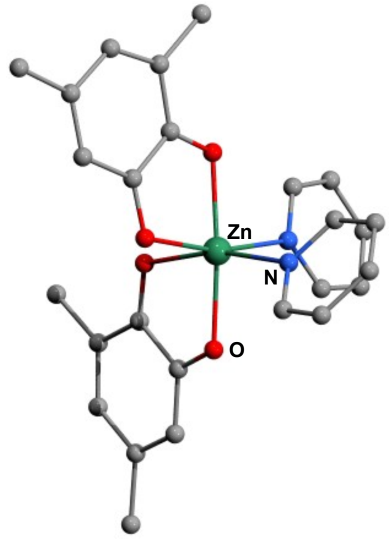

Figure S33: Ball-and-stick representation of a single molecule of Zn(3,5-dtbsq)$_2$(pyridine)$_2$ (**4**).

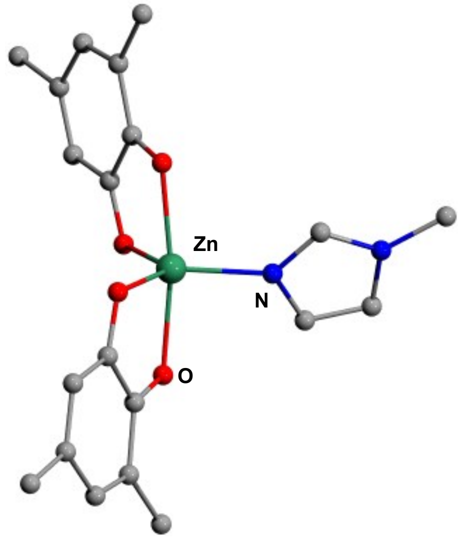

Figure S34: Ball-and-stick representation of a single molecule of Zn(3,5-dtbsq)$_2$(N-methyl imidazole)$_1$ (**5**).

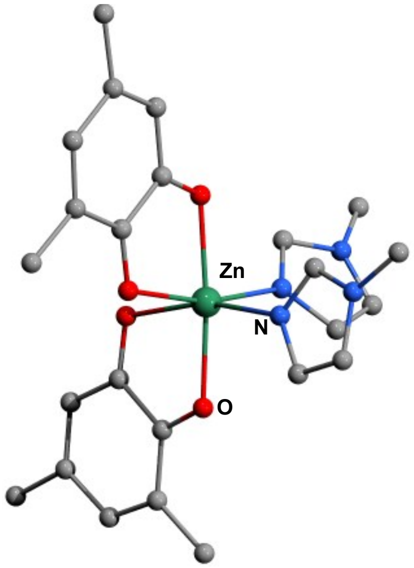

Figure S35: Ball-and-stick representation of a single molecule of Zn(3,5-dtbsq)$_2$(N-methyl imidazole)$_2$ (**6**).

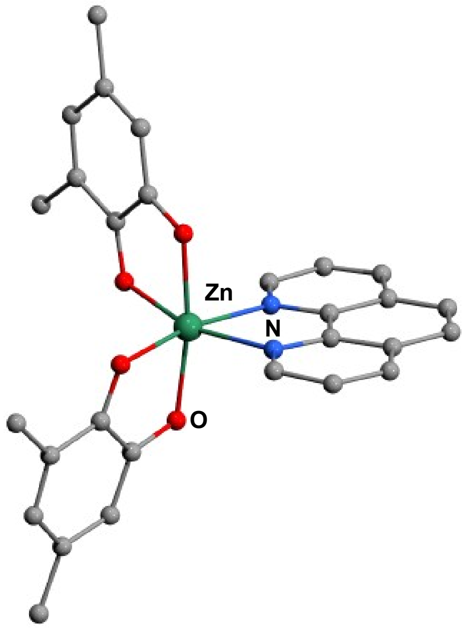

Figure S36: Ball-and-stick representation of a single molecule of Zn(3,5-dtbsq)$_2$(1,10-phenanthroline)-(toluene) (**7**). Note that the toluene solvate molecule has been omitted for clarity.

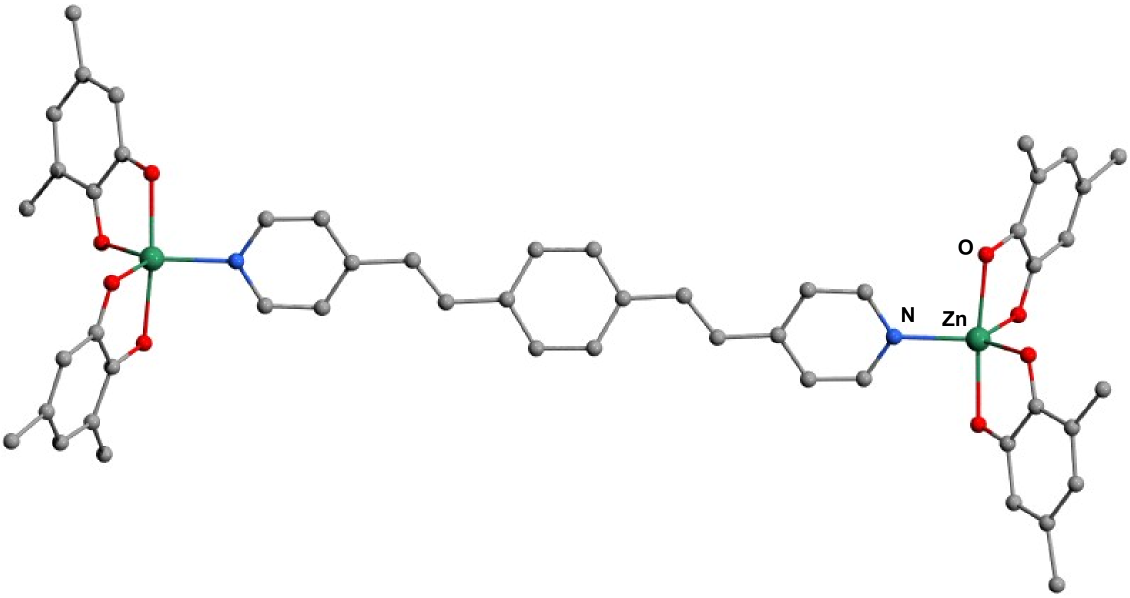

Figure S37: Ball-and-stick representation of a single molecule of Zn_2(3,5-dtbsq)$_4$(1,4-bis(2-(pyridin-4-yl)vinyl)benzene) (**9**).

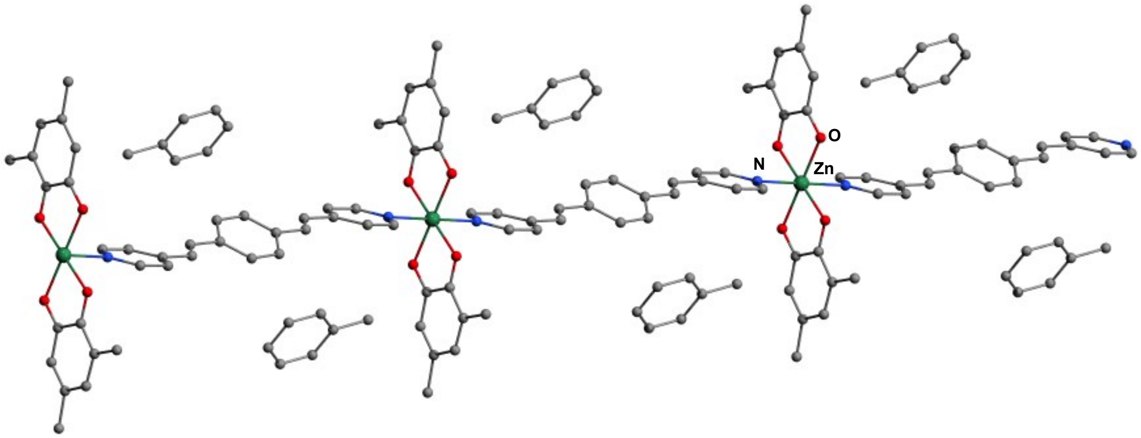

Figure S38: Ball-and-stick representation of a fragment of a linear coordination polymer chain in Zn(3,5-dtbsq)$_2$(1,4-bis(2-(pyridin-4-yl)vinyl)benzene) (**10**).

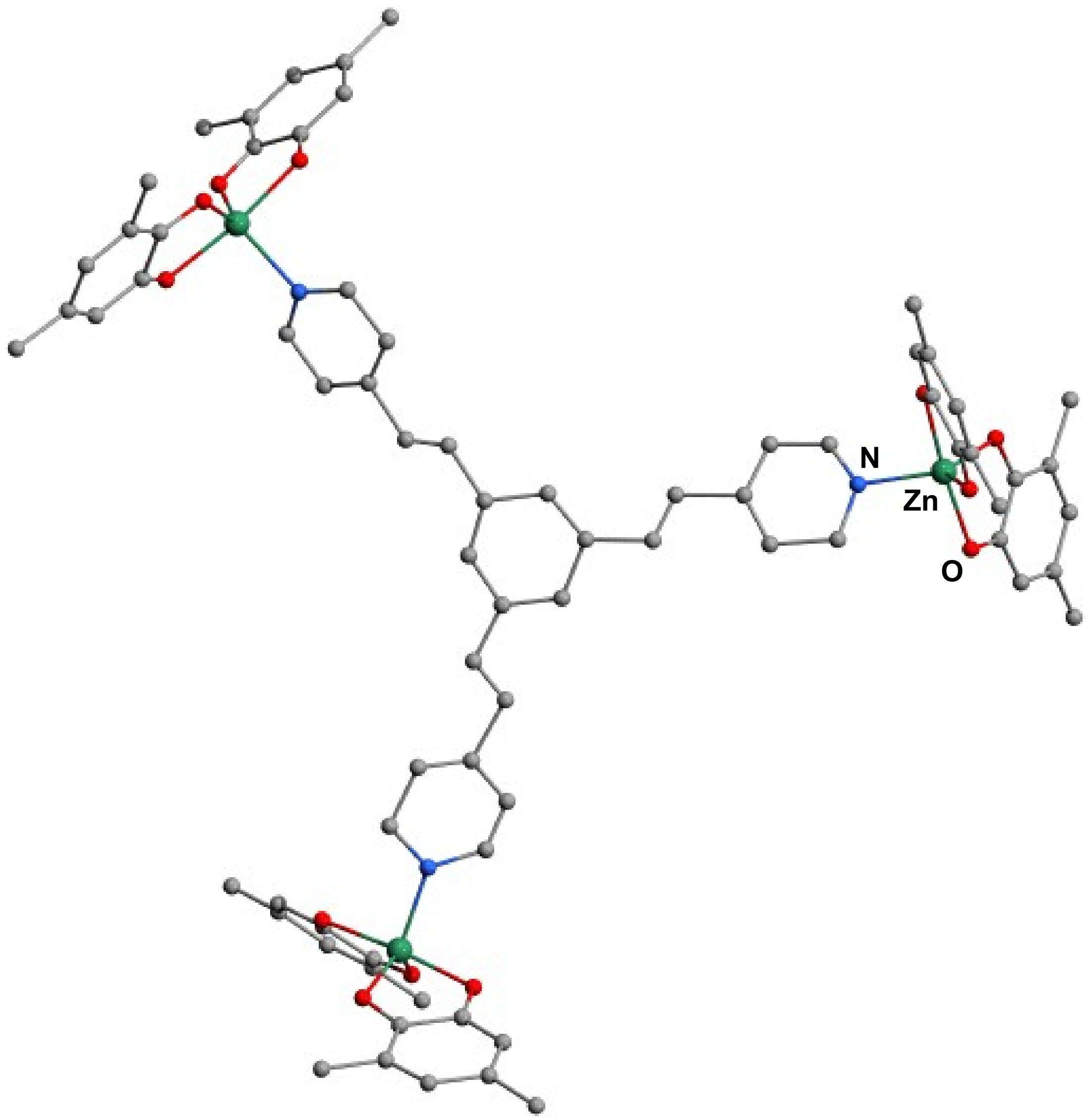

Figure S39: Ball-and-stick representation of a single molecule of Zn_3(3,5-dtbsq)$_6$(1,3,5-tris(2-(pyridin-4-yl)vinyl)benzene) (**11**). Included toluene molecules, partially disordered, have been removed for clarity.

3. FTIR-ATR Spectra

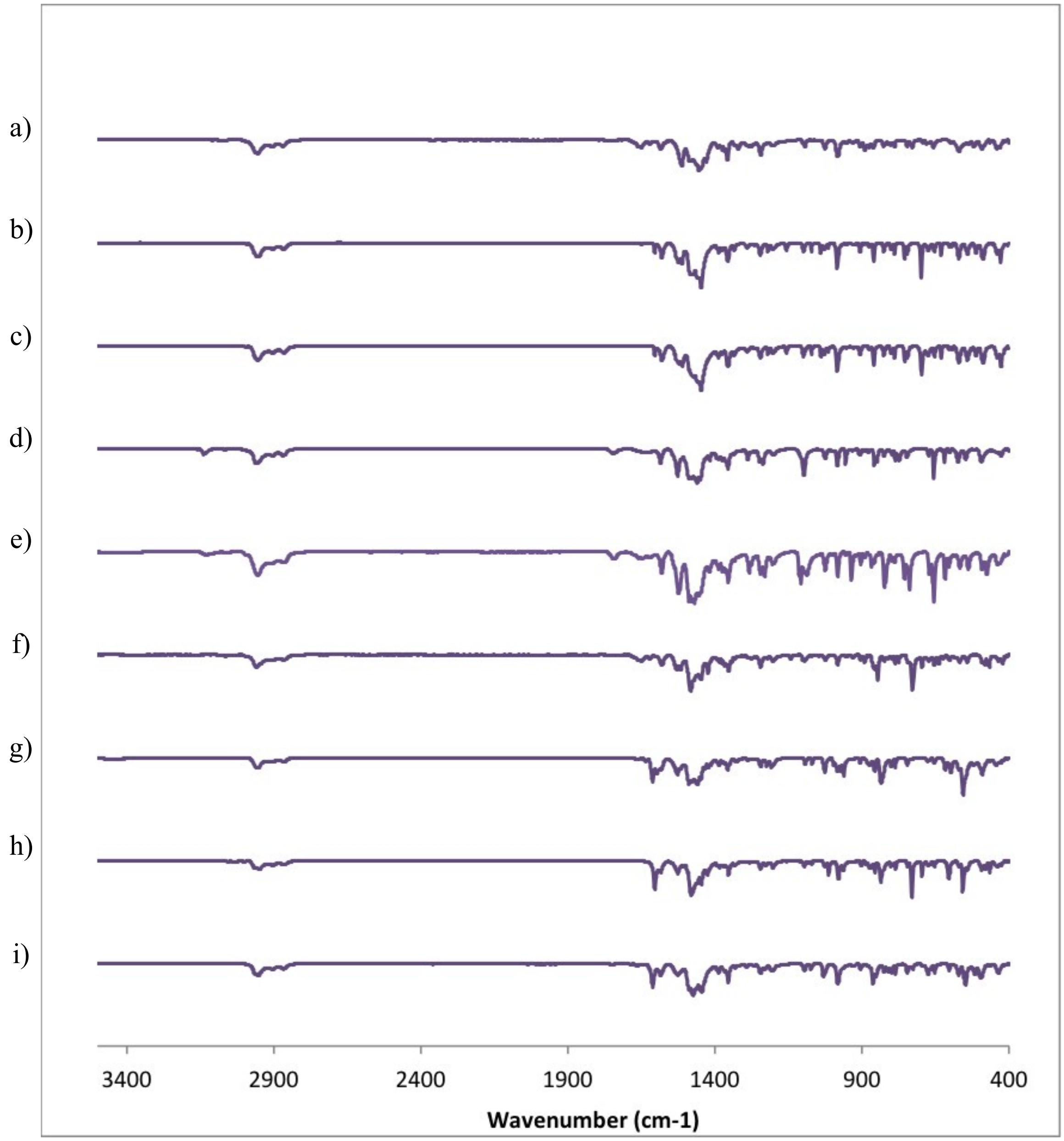

Figure S40. FT-IR Spectra of a) **2**; b) **3**; c) **4**; d) **5**; e) **6**; f) **7**; g) **9**; h) **10**; i) **11**

4. UV-Vis Spectra

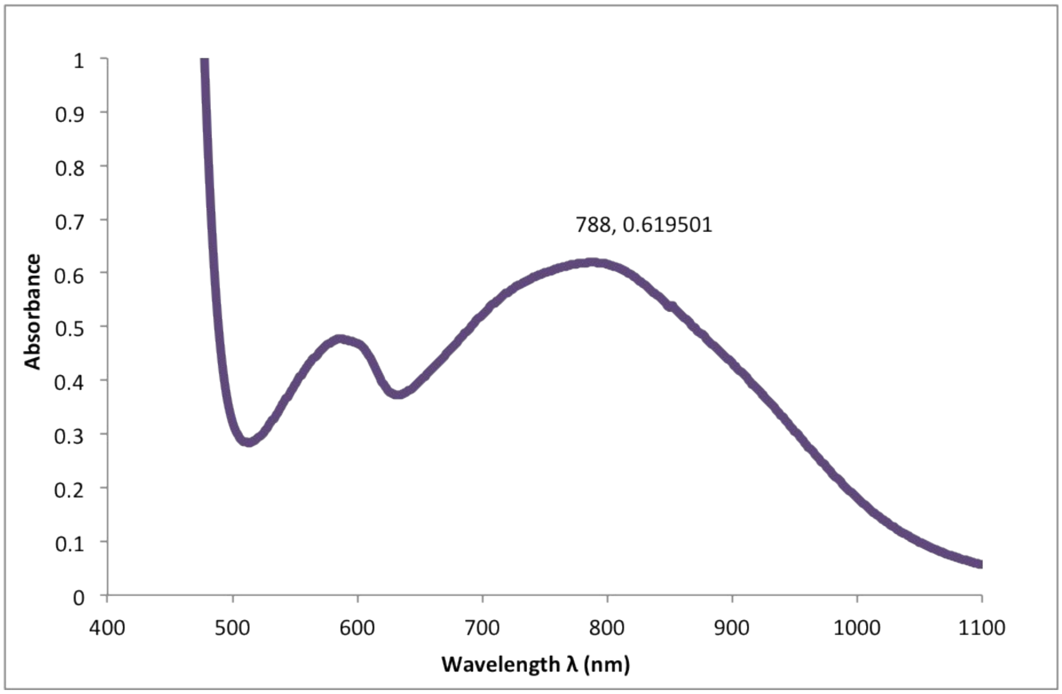

Figure S41. UV-Vis Spectrum of complex **2** ($1x10^{-3}$M)

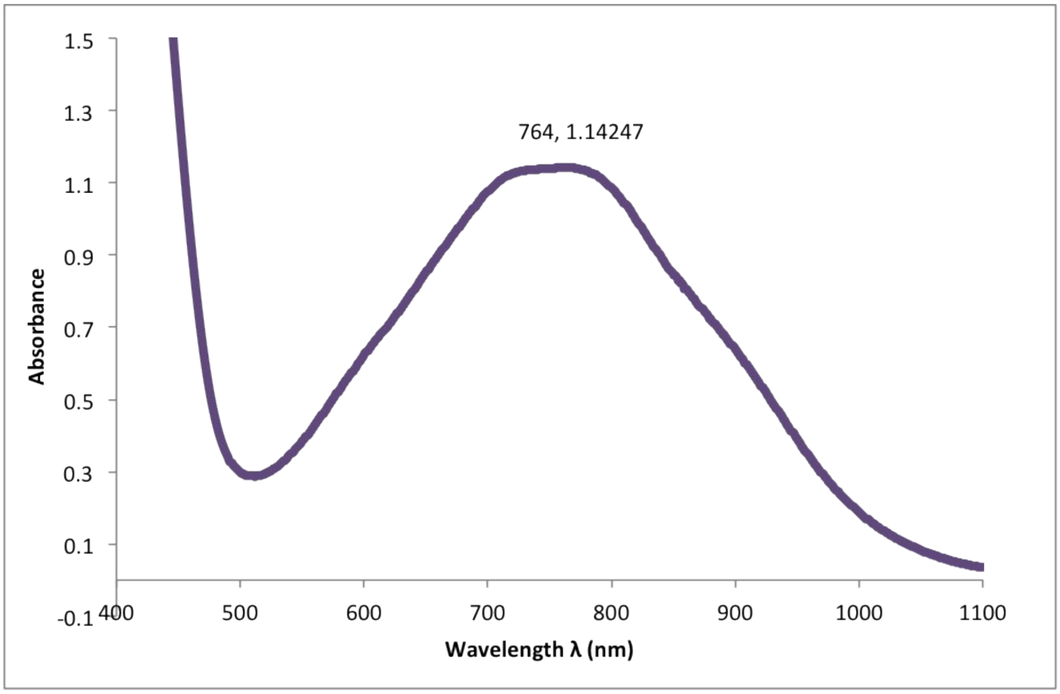

Figure S42. UV-Vis Spectrum of complex **3** ($1x10^{-3}$M)

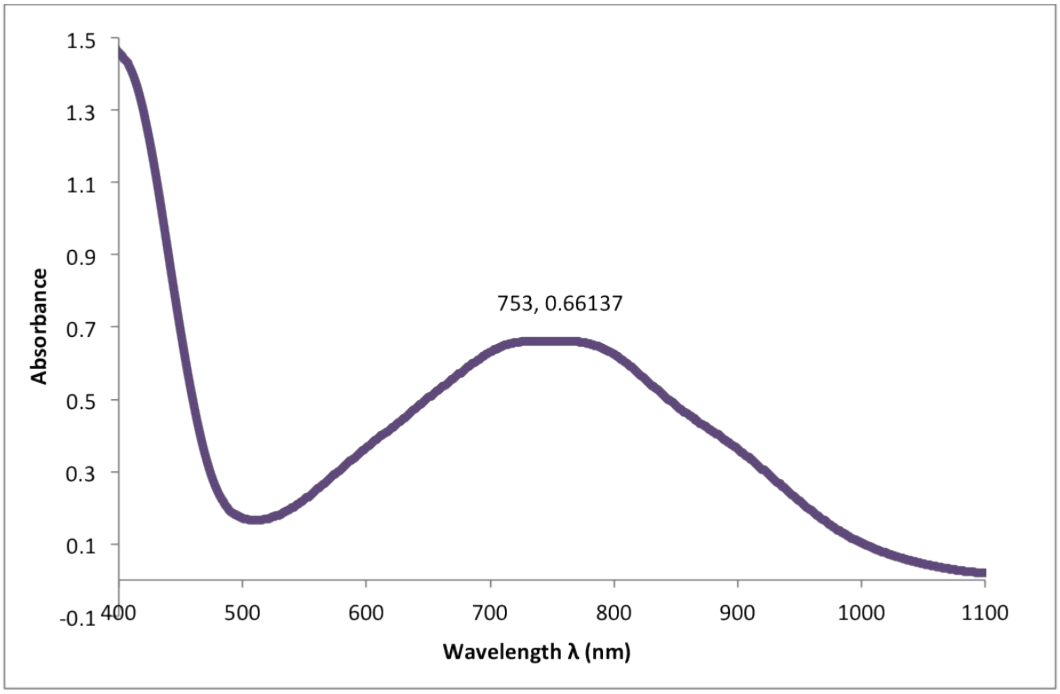

Figure S43. UV-Vis Spectrum of complex **4** ($1x10^{-3}M$)

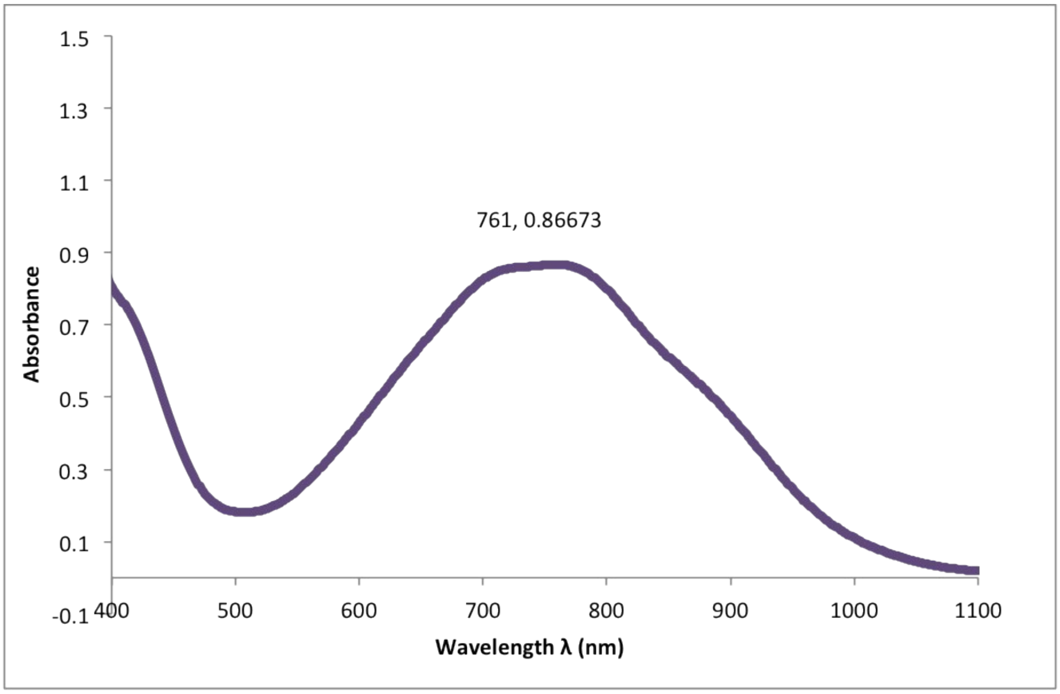

Figure S44. UV-Vis Spectrum of complex **5** ($1x10^{-3}M$)

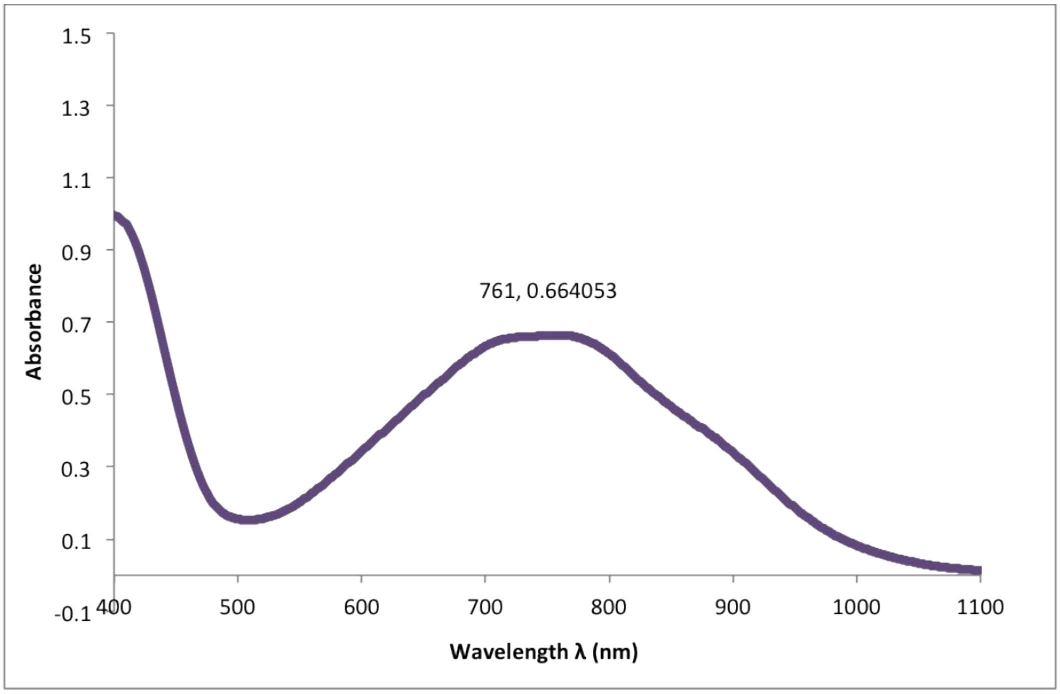

Figure S45. UV-Vis Spectrum of complex **6** ($1x10^{-3}$M)

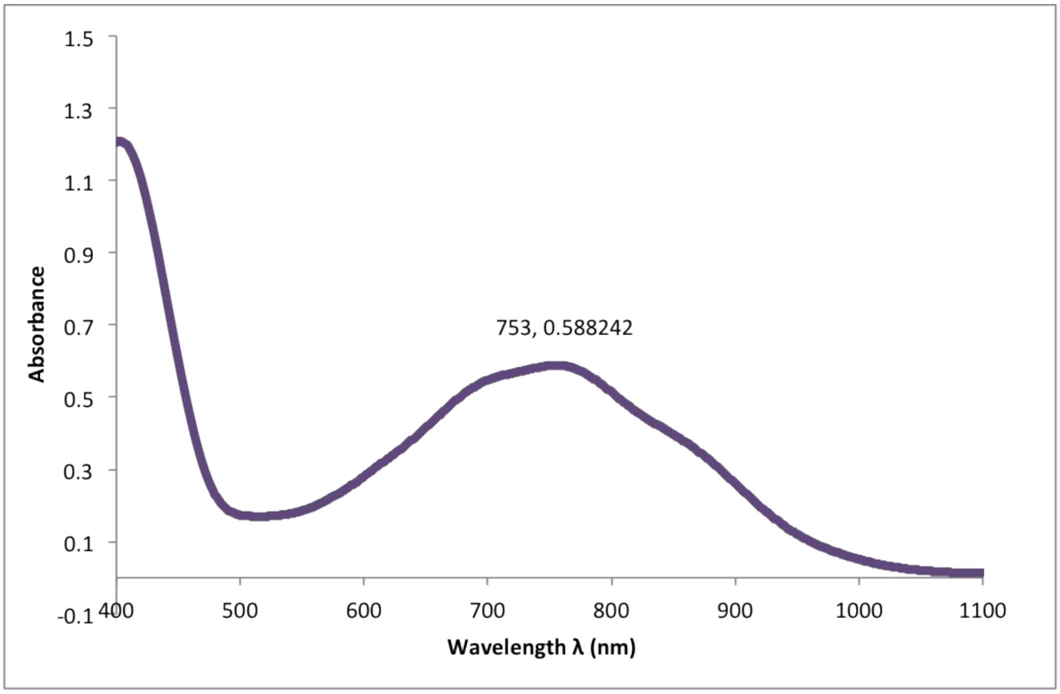

Figure S46. UV-Vis Spectrum of complex **7** ($1x10^{-3}$M)

Complex	λmax (nm)	Concentration (mol/L)	Peak Absorbance	ε for λmax ((L/mol)(1/cm))
2	788	0.001	0.6195	619.5
3	764	0.001	1.14247	1142.47
4	753	0.001	0.66137	661.37
5	761	0.001	0.86673	866.73
6	761	0.001	0.664053	664.053
7	753	0.001	0.588242	588.242

Table S1. Tabulated UV-Vis spectral features of complexes **2**-**7**

5. EPR Spectra

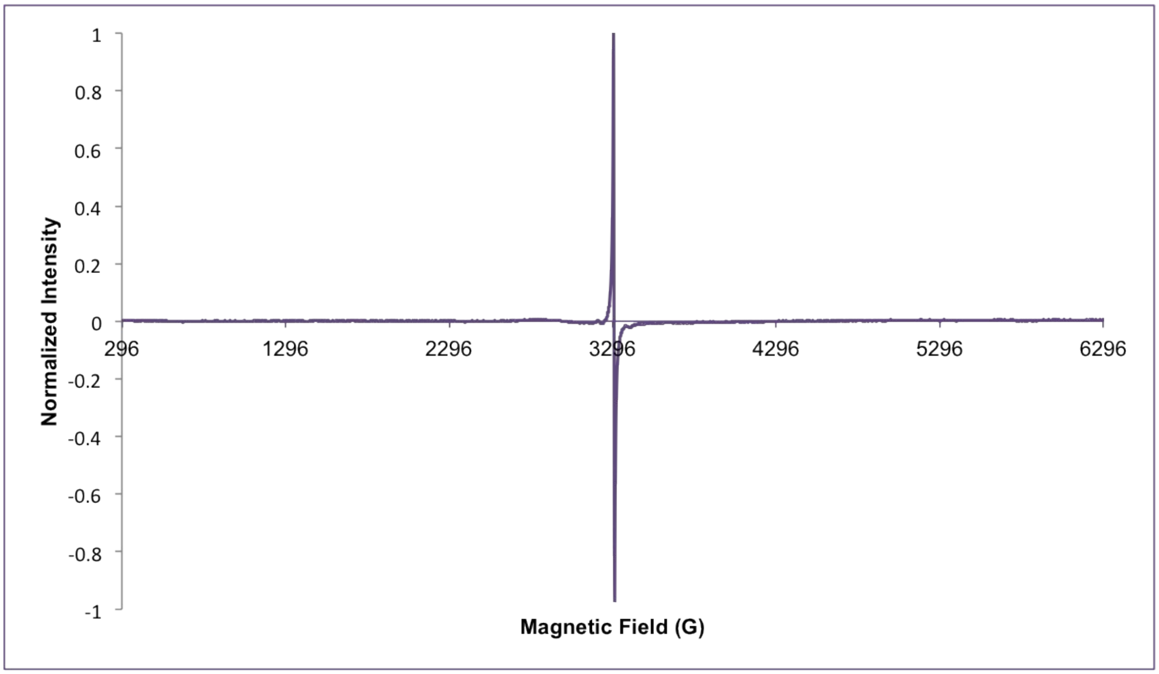

Figure S47. Complex **2**

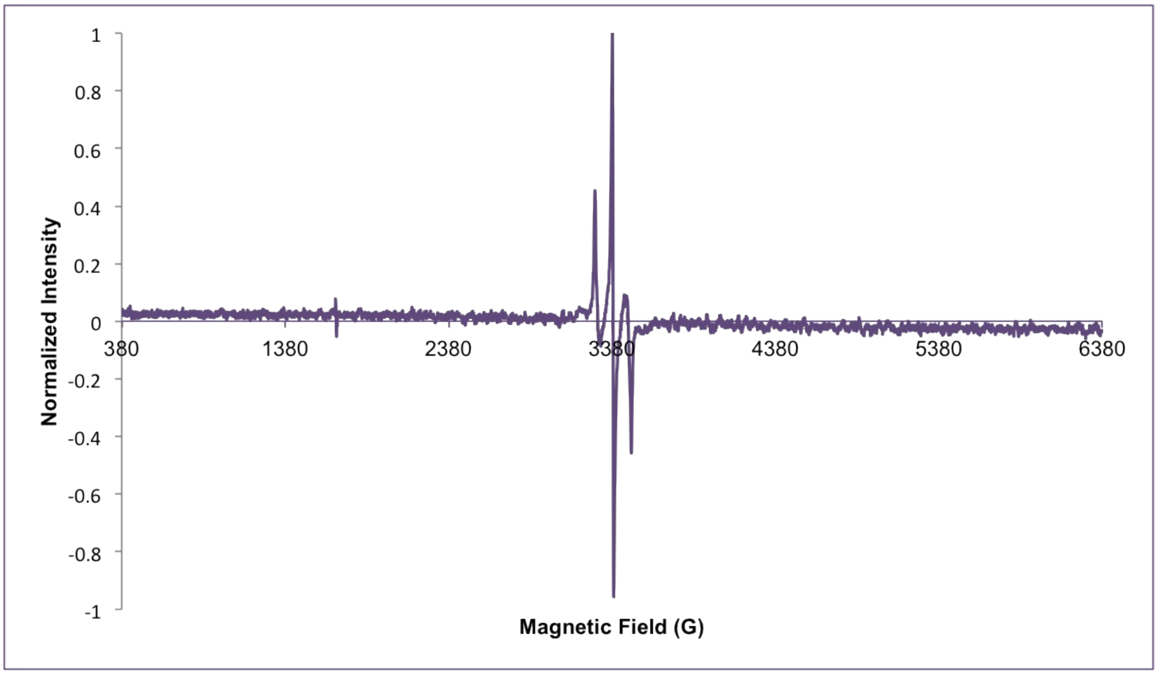

Figure S48. Complex **3**

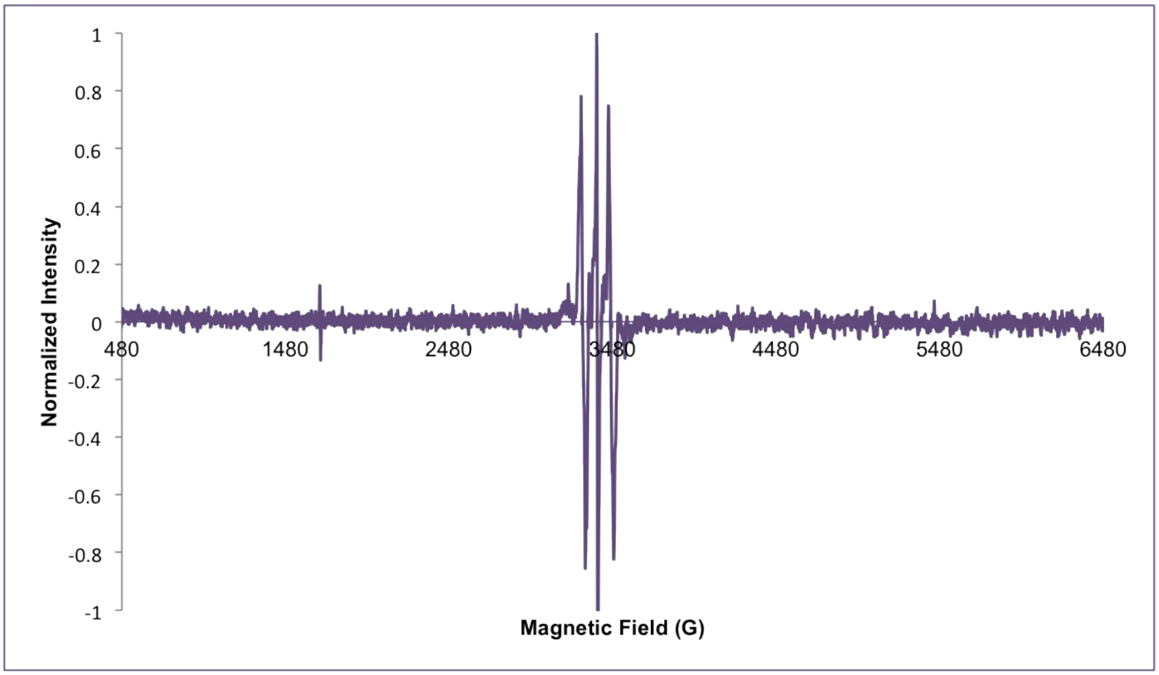

Figure S49. Complex **4**

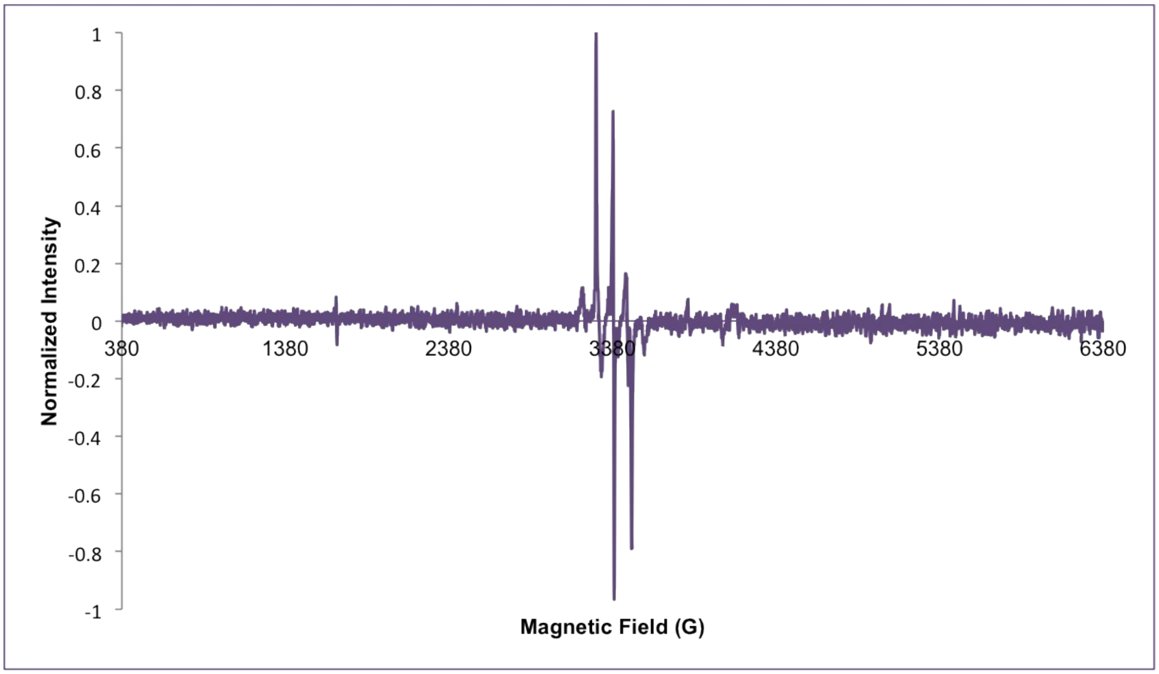

Figure S50. Complex **5**

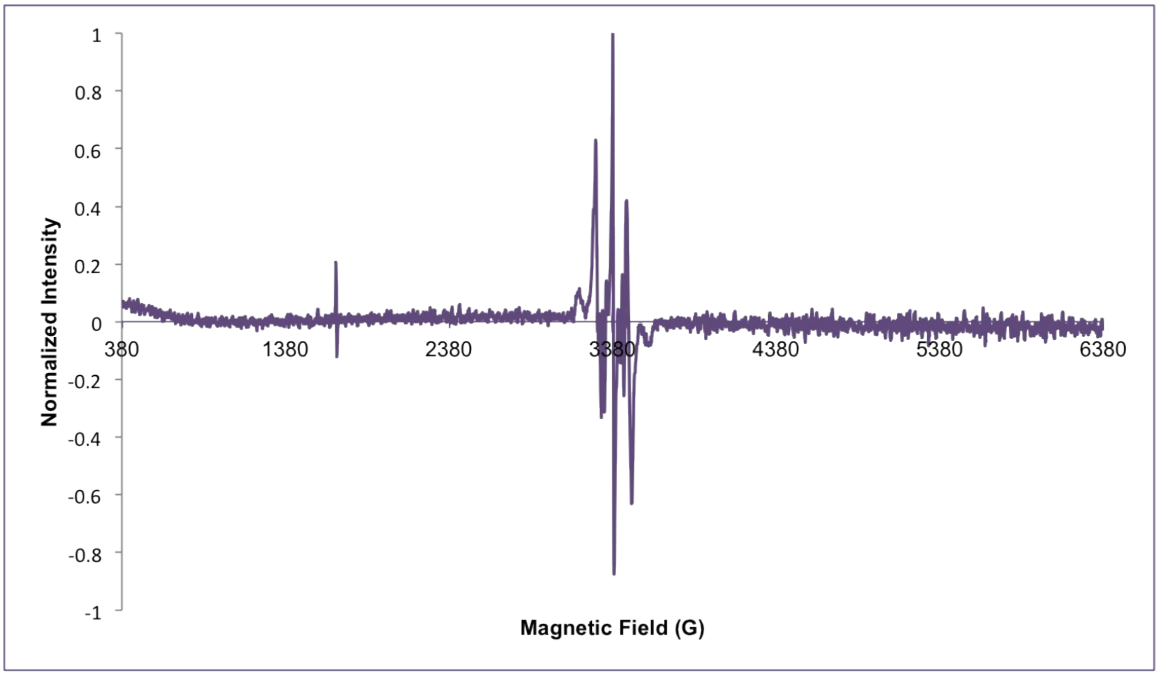

Figure S51. Complex **6**

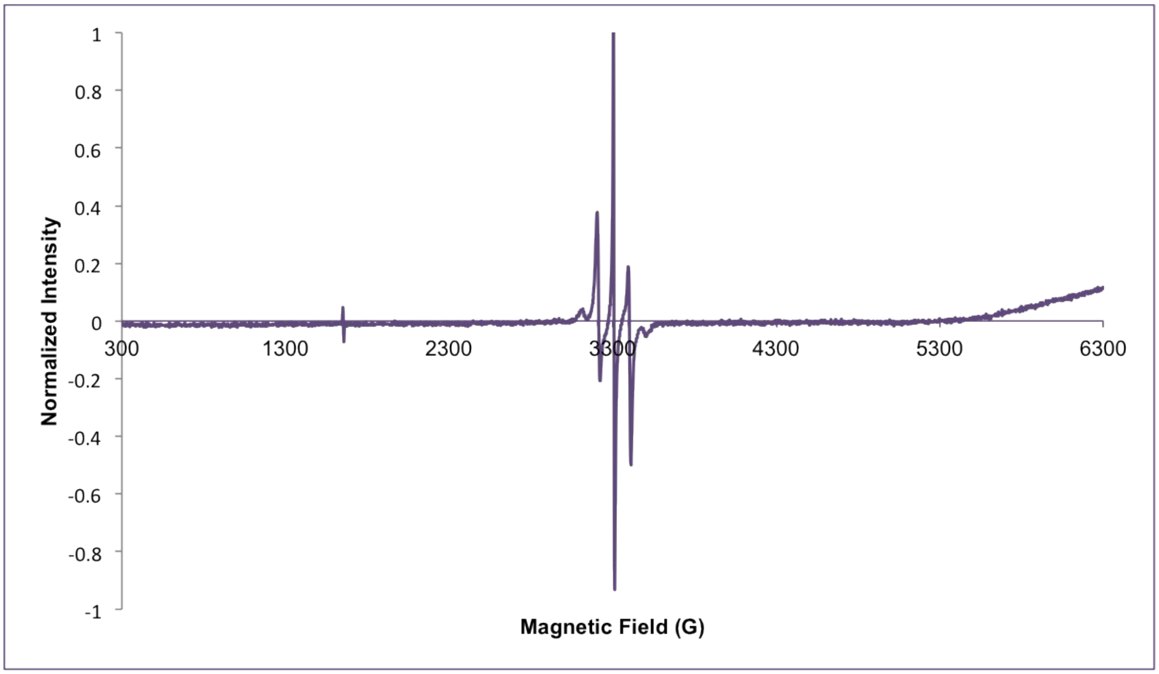

Figure S52**.** Complex **7**

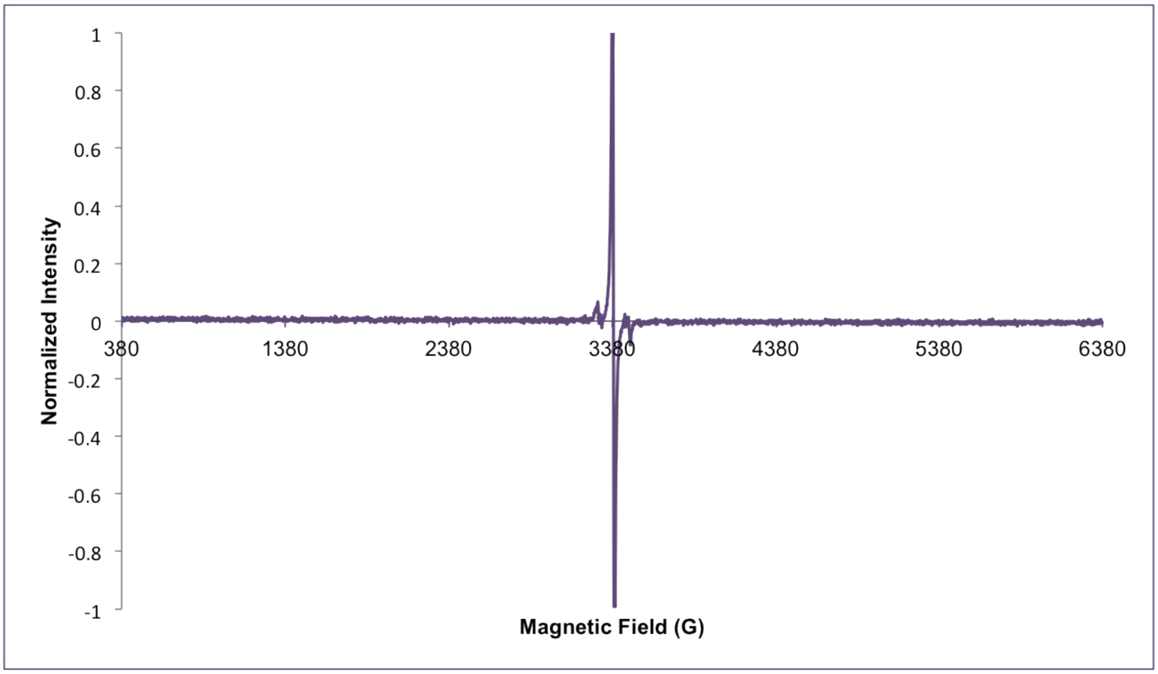

Figure S53. Complex **9**

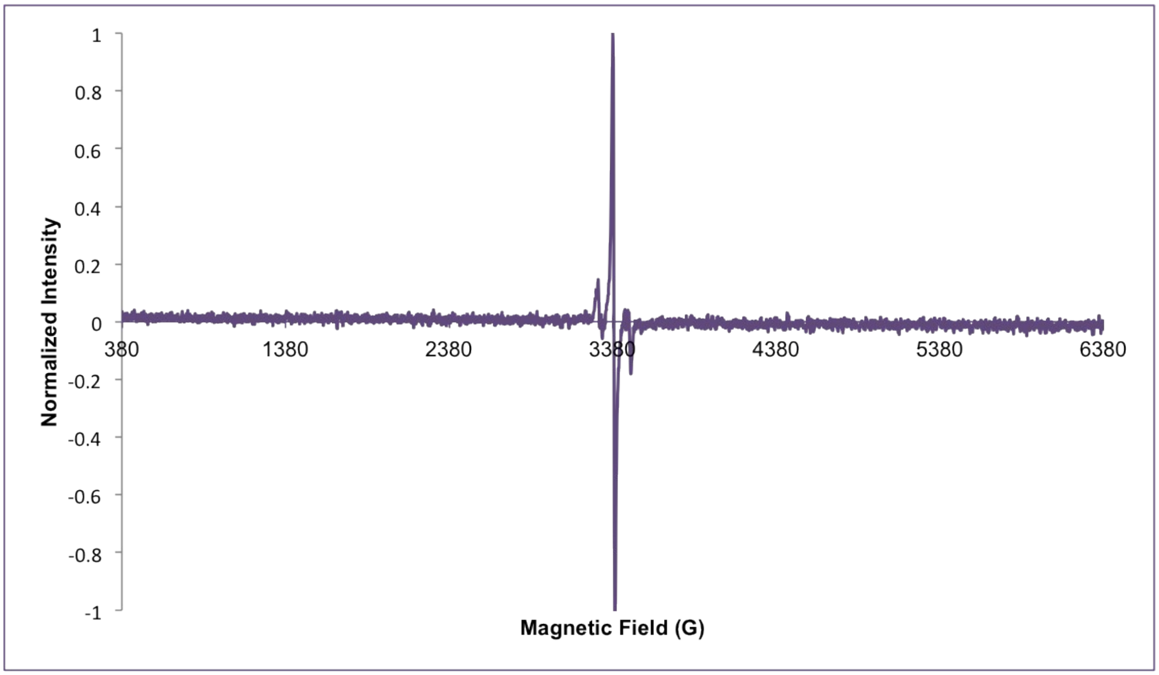

Figure S54. Complex **10**

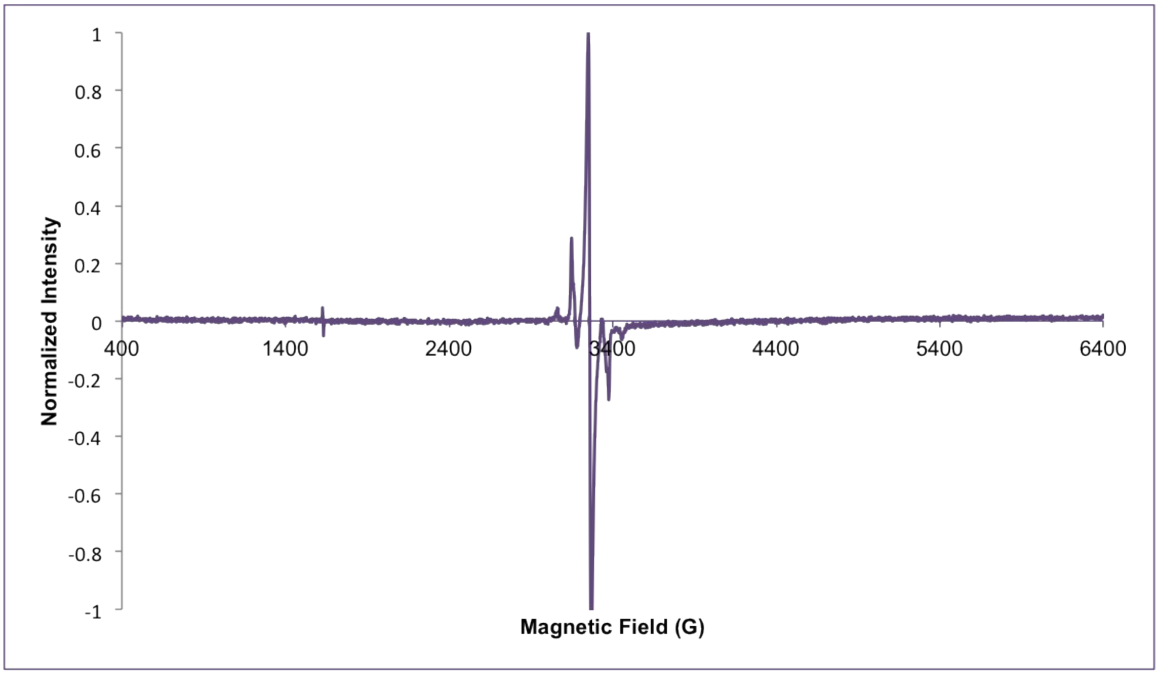

Figure S55. Complex **11**

6. TGA thermograms

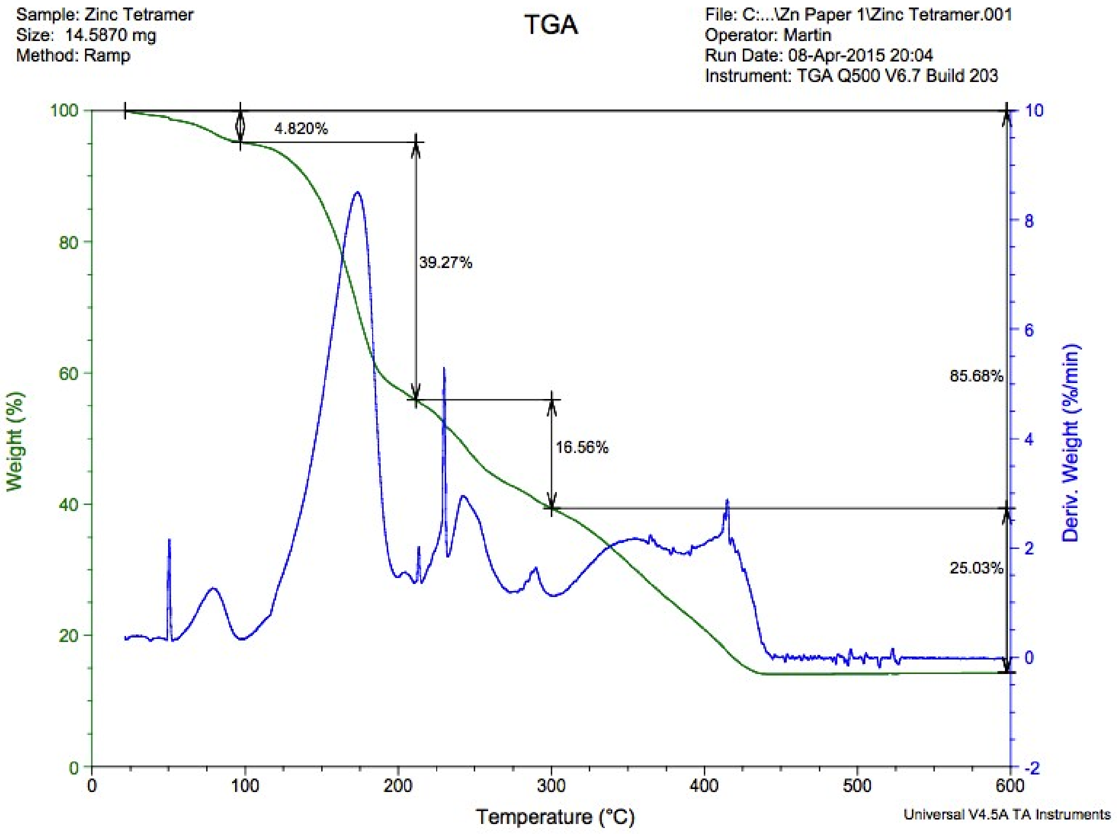

Figure S56. TGA thermogram of [Zn(3,5-dtbsq)$_2$]$_4$-(toluene) tetranuclear complex **2** Theoretical % remaining 15.4%; Experimental % remaining 14.32%.

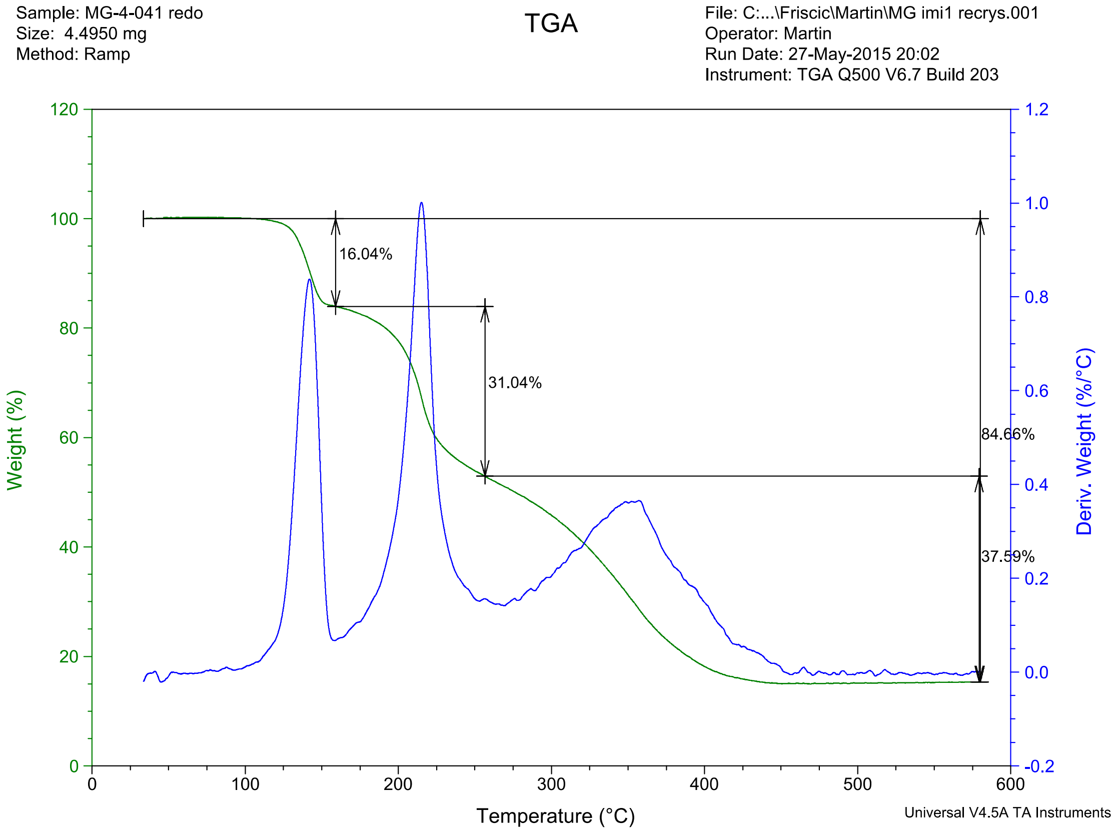

Figure S57. TGA thermogram of Zn_2(3,5-dtbsq)$_4$(pyridine)$_2$ binuclear complex **3**. Theoretical % remaining 13.9%; Experimental % remaining 15.3%

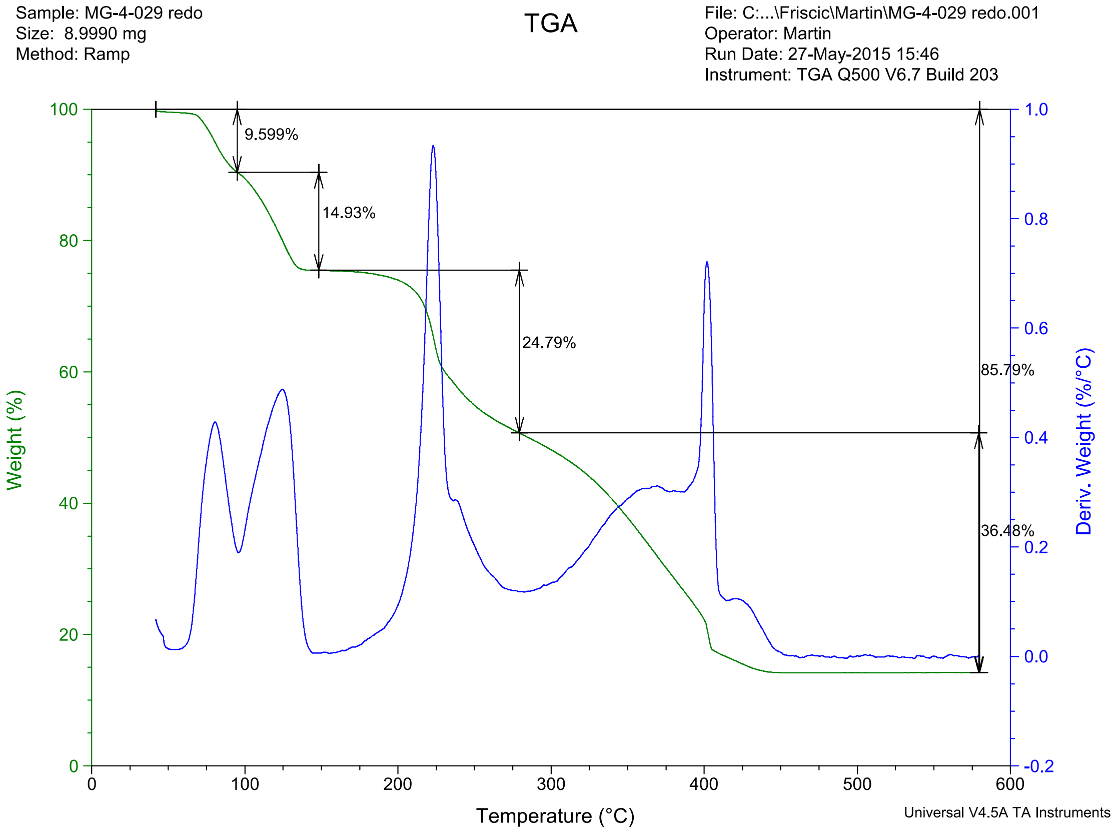

Figure S58. TGA thermogram of Zn(3,5-dtbsq)$_2$(pyridine)$_2$ mononuclear complex **4**. Theoretical % remaining 12.3%; Experimental % remaining 14.2%

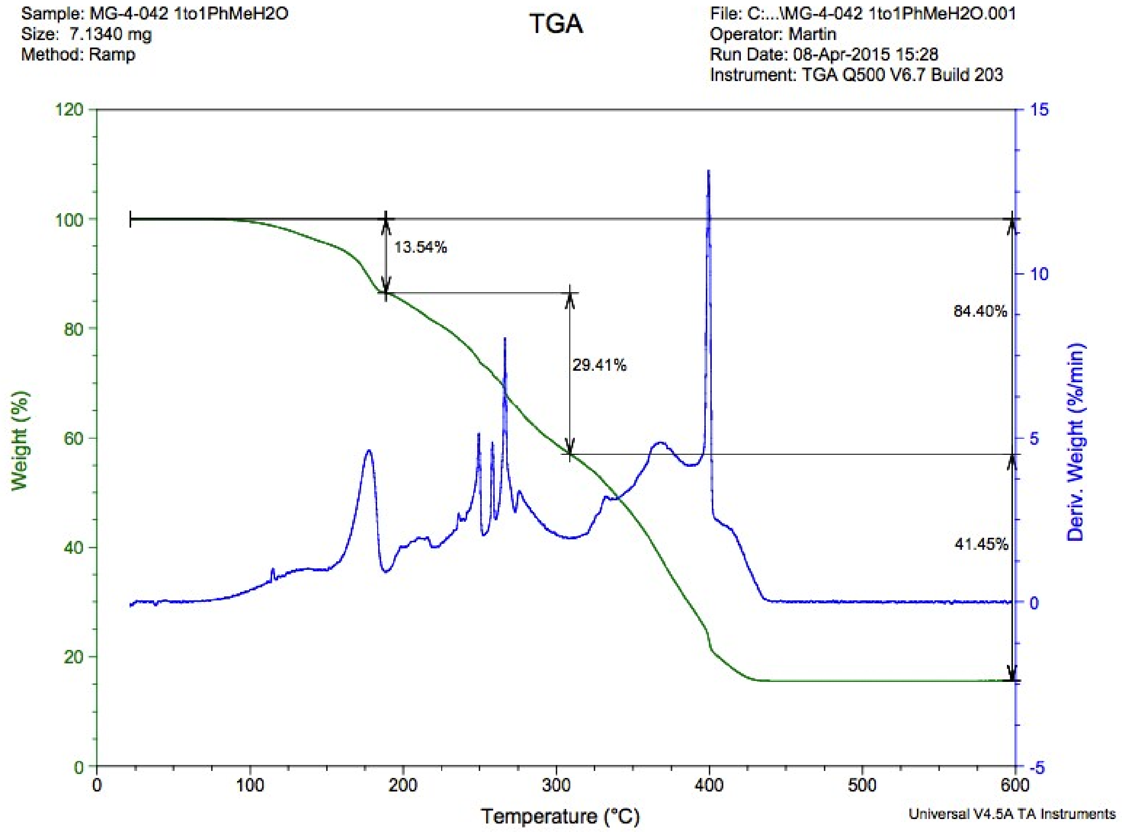

Figure S59. TGA thermogram of Zn(3,5-dtbsq)$_2$(N-Methyl Imidazole)$_1$ mononuclear complex **5***.* Theoretical % remaining 13.8%; Experimental % remaining 15.6%

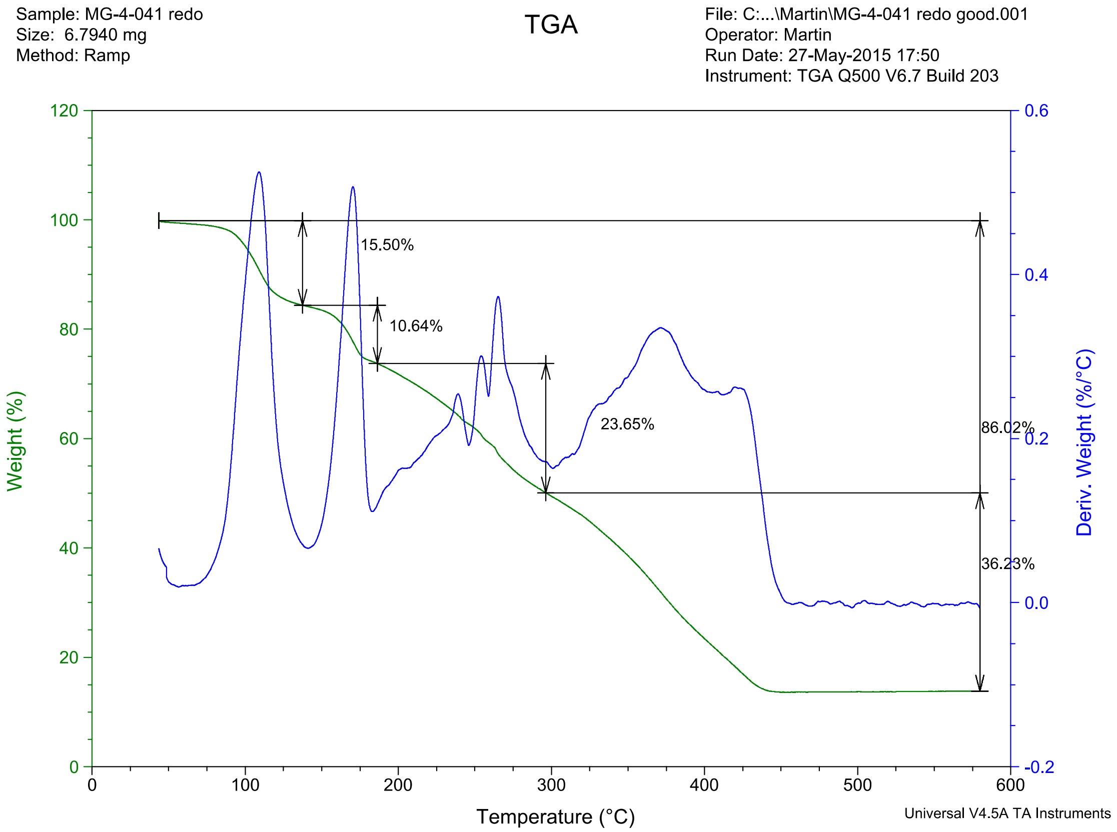

Figure S60. TGA thermogram of Zn(3,5-dtbsq)$_2$(N-Methyl Imidazole)$_2$ mononuclear complex **6**.Theoretical % remaining 12.1%; Experimental % remaining 14.0%

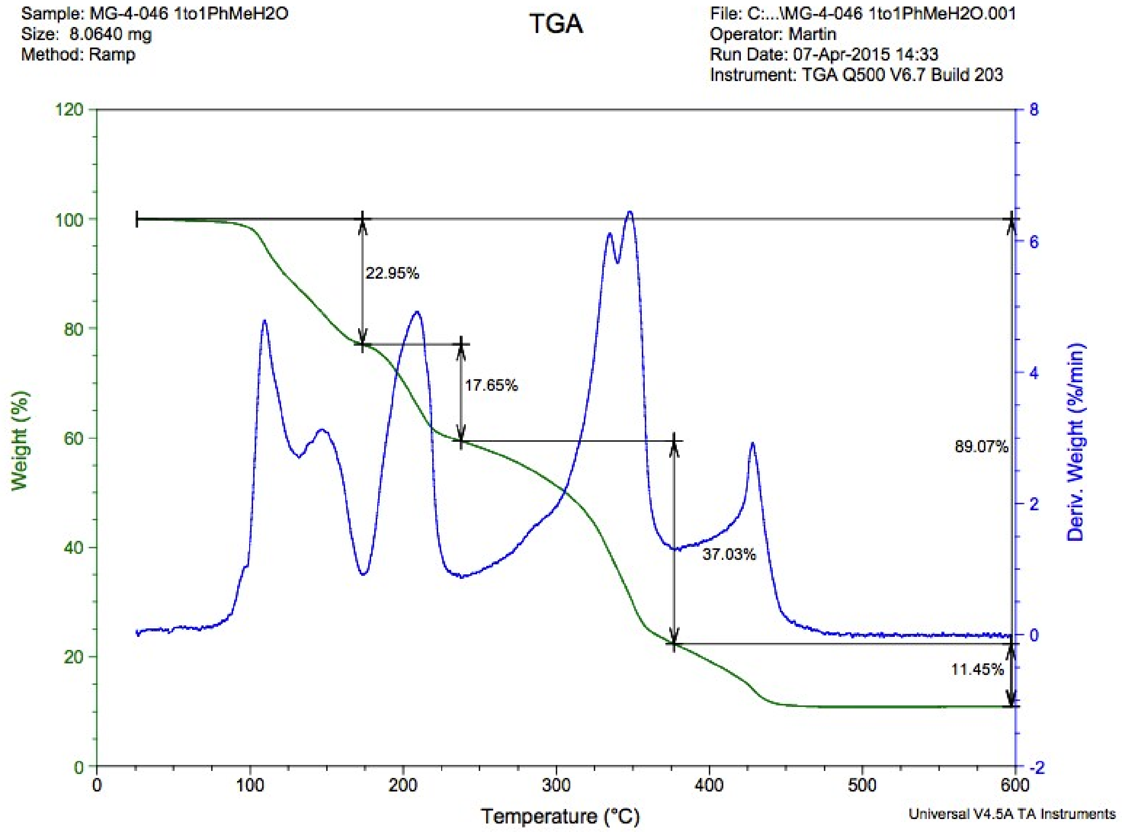

Figure S61. TGA thermogram of Zn(3,5-dtbsq)$_2$(1,10-phenanthroline)-(toluene) mononuclear complex **7**. Theoretical % remaining 10.2%; Experimental % remaining 10.9%

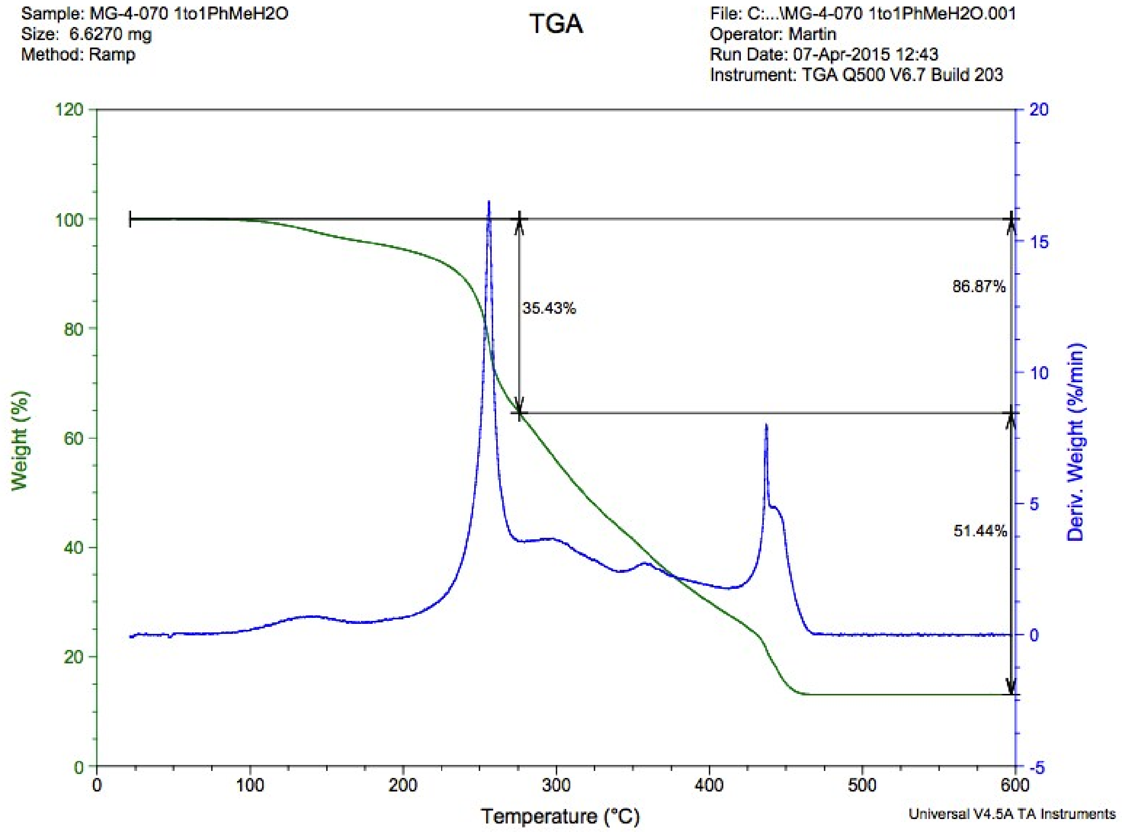

***Figure S62*.** TGA thermogram of Zn_2(3,5-dtbsq)$_4$(1,4-bis(2-(pyridin-4-yl)vinyl)benzene) binuclear complex **9**. Theoretical % remaining 12.6%; Experimental % remaining 13.1%

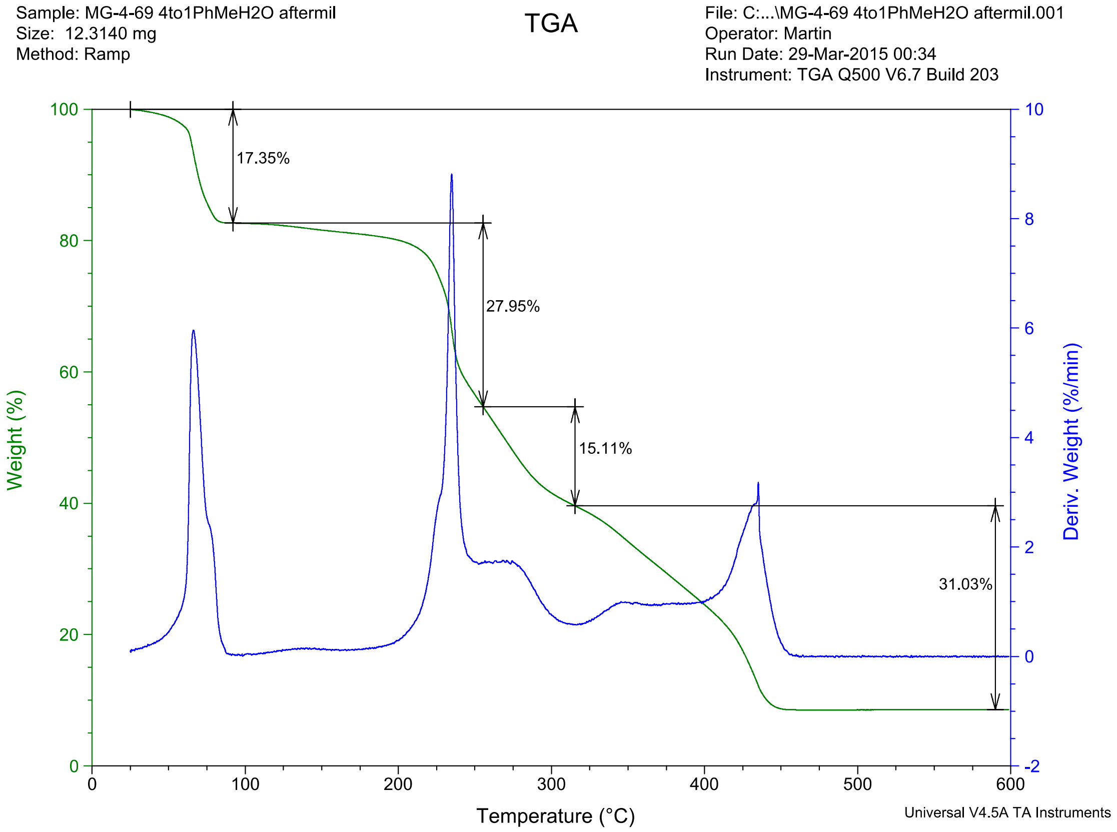

***Figure S63*.** TGA thermogram of Zn(3,5-dtbsq)$_2$(1,4-bis(2-(pyridin-4-yl)vinyl)benzene)-(toluene)$_2$ coordination polymer **10**. Theoretical % remaining 8.4%; Experimental % remaining 8.56%

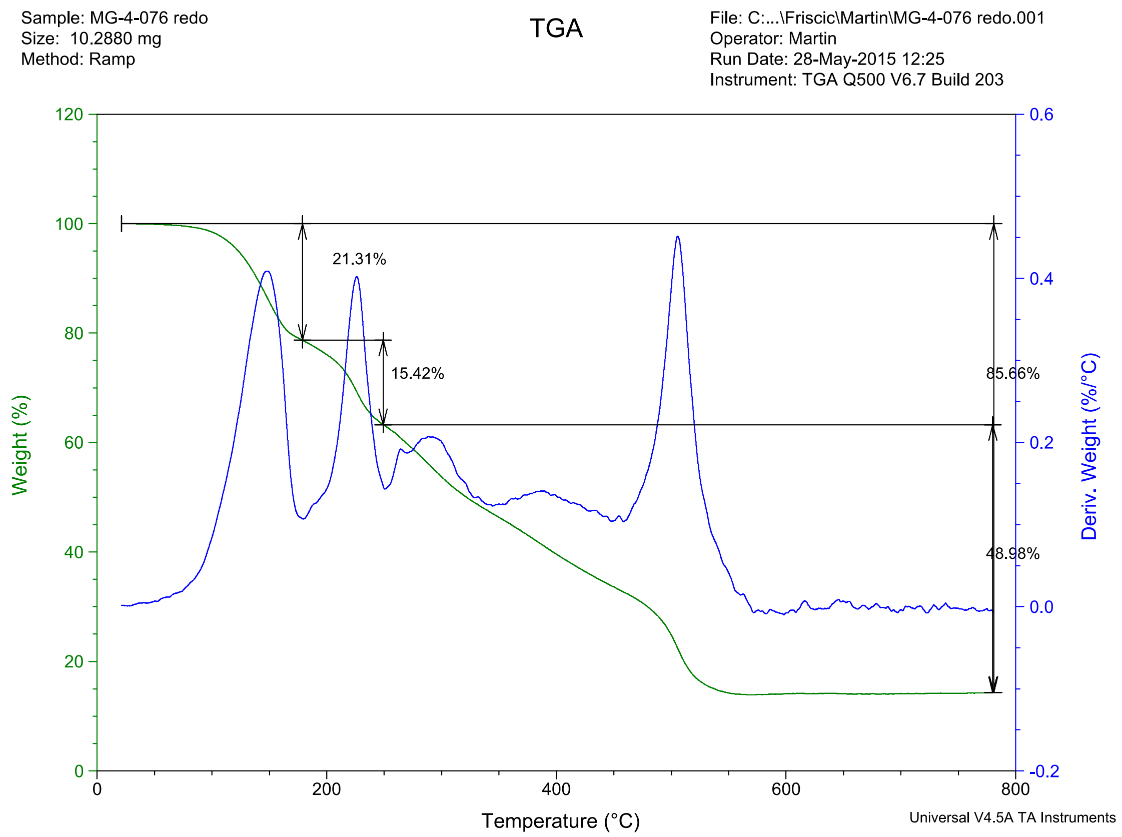

Figure S64. TGA thermogram of Zn_3(3,5-dtbsq)$_6$(1,3,5-tris(2-(pyridin-4-yl)vinyl)benzene) trinuclear complex **11**. Theoretical % remaining 12.8%; Experimental % remaining 14.3%
